# The effects of challenge and threat states on performance outcomes: An updated review and meta-analysis of recent findings

**DOI:** 10.17179/excli2024-7995

**Published:** 2025-01-16

**Authors:** Adrian Hase, Maximilian Nietschke, Maciej Kloskowski, Kacper Szymanski, Lee Moore, Jeremy P. Jamieson, Maciej Behnke

**Affiliations:** 1 Medicine Section, University of Fribourg, Fribourg, Switzerland; 2 Institute of Philosophy, University of Bern, Bern, Switzerland; 3 Adam Mickiewicz University, Poznan, Poland; 4 University of Bath, Bath, United Kingdom; 5 University of Rochester, Rochester, USA

**Keywords:** challenge-threat index, demand resource evaluations, motivated performance, biopsychosocial, cognitive appraisal, psychophysiology

## Abstract

The biopsychosocial model (BPSM) of challenge and threat provides a framework for understanding stress responses in motivated performance situations, including how stress relates to performance. In this model, experiences of challenge - characterized by evaluations of personal coping resources matching or exceeding situational demands - elicit approach-oriented patterns of physiological responding and tend to facilitate performance, whereas threat - characterized by demands exceeding resources - elicit avoidance-oriented patterns of physiological responding and tend to impair performance. Extant systematic reviews and meta-analyses support the idea that challenge facilitates performance relative to threat (Behnke and Kaczmarek, 2018[8]; Hase et al., 2019[50]). The present systematic review and meta-analysis builds on this evidence base by examining whether conclusions replicate in recent research (post-2017), which is important given seismic cultural shifts tied to a worldwide pandemic, civil unrest, and skyrocketing mental health problems tied to stress. The analysis included 62 studies published between 2017 and 2024 (total *N* = 7,418 participants). The meta-analytic findings indicate that individuals in a challenge state achieve better performance outcomes than those in a threat state across multiple domains (e.g., education, sport). While effect sizes were small, the risk of bias was generally low. These results reaffirm the utility of the BPSM and emphasize the importance of stress responses in influencing performance outcomes. These data also have the potential to inform future research on this topic by shedding light on expectable effect sizes and highlighting potential influences of publication bias and replicability issues.

## The Effects of Challenge and Threat States on Performance Outcomes: An Updated Review and Meta-Analysis of Recent Findings

Psychophysiological stress responses are crucial for determining performance outcomes across myriad high-pressure contexts, including athletics, education, and healthcare domains, to name a few (Hase et al., 2019[50]). Understanding the processes that undergird individual psychophysiological stress responses in such contexts can help prevent underperformance and optimize outcomes (e.g., mental and physical health; McLoughlin et al., 2024[79]). The Biopsychosocial Model (BPSM) of Challenge and Threat (CAT) (e.g., Blascovich and Tomaka, 1996[16]) integrated concepts from Lazarus and Folkman's (1984[70]) appraisal theory of stress as well as Dienstbier's (1989[33]) theory of physiological toughness to offer a comprehensive framework of stress responses in motivated performance situations, or situations that are goal-relevant, evaluative, and potentially stressful. The BPSM has been applied across a range of fields, including sports (Moore et al., 2012[90], 2013[87][91], 2015[89], 2018[92]), education (Chalabaev et al., 2012[27]; Jamieson et al., 2016[59]; Schneider, 2004[109]), and medicine (Moore et al., 2014[88]; Vine et al., 2013[130]). Here, we aimed to summarize the current findings on the role of CAT states in the performance context, updating our previous systematic review (Hase et al., 2019[50]) and a meta-analysis (Behnke and Kaczmarek, 2018[8]).

### Biopsychosocial model of challenge and threat

The BPSM presents the role of psychophysiological stress responses in motivated performance situations that require instrumental cognitive responses and often involve social evaluation (Blascovich and Mendes, 2010[14]). A fundamental principle is the concept that cognitive evaluations of situational demands and personal coping resources operating in an integrated manner determine challenge- and threat-type stress responses in motivated performance contexts (Blascovich and Mendes, 2010[14]; Jamieson, 2017[55]; Mendes and Park, 2014[82]). The BPSM conceptualizes “demands” and “resources” as multidimensional constructs. 

Demand evaluations may include uncertainty, danger, and effort, which can be independent or intertwined. Similarly, resource evaluations encompass factors such as familiarity, knowledge, skills, dispositional traits, and social support, which may also overlap. Importantly, resource and demand evaluations can fluctuate independently (i.e., be ontologically distinct) or function as bipolar factors affecting both processes (Seery, 2011[111]). For instance, familiarity and uncertainty evaluations can simultaneously influence perceptions of both resources and demands (Blascovich, 2008[13]; Seery, 2011[111]). These evaluations determine whether an individual experiences a challenge- or threat-type response in a performance situation. A challenge response occurs when coping resources are appraised as matching or exceeding situational demands, whereas a threat response emerges when demands are perceived to outweigh coping resources.

While CAT states are often described as distinct, they are better understood as endpoints on a continuum of potential responses rather than as binary states (Seery, 2011[111]). According to the BPSM, patterns of CAT cognitive evaluations determine physiological responses to stressors in performance contexts (see Mendes and Park, 2014[82], for a review). These stress responses are derived from activation of the sympathetic-adrenal-medullary (SAM) and hypothalamic-pituitary-adrenal (HPA) axes. Both CAT states are accompanied by SAM activation, leading to the synthesis and secretion of catecholamines like epinephrine and norepinephrine, which increase ventricular contractility (i.e., speeding up heart rate) and dilate blood vessels (Brownley et al., 2000[22]). Both challenge- and threat-type stress responses are characterized by high sympathetic arousal. Thus, SAM-dominated challenge-type physiological responses are characterized by increased cardiac output - a volumetric measure of blood pumped by the heart across time - and decreased resistance in the peripheral vasculature (termed total peripheral resistance; Blascovich, 2008[13]). These changes allow for the effective delivery of oxygenated blood flow to the brain and peripheral sites (e.g., muscles). Challenge-type physiological responses also allow for a rapid onset and offset of stress responses: resources are mobilized rapidly for performance, and individuals return to homeostasis quickly after performance is completed (Seery, 2011[111]. Threat-type responses, however, not only elicit SAM activation but also activate the HPA axis, which produces a more prolonged stress response due to the longer half-life of cortisol (the hormonal product of HPA activation). HPA activation tempers SAM effects and results in reduced (or little change in) cardiac output and increased resistance in the peripheral vasculature to concentrate blood in the core of the body in preparation for anticipated damage or defeat (Blascovich and Mendes, 2010[14]; Jamieson, 2017[55]). 

More downstream, CAT responses have important consequences for decisions, behavior, and performance (Blascovich, 2008[13]; Seery, 2013[112]). For instance, CAT states exhibit differential patterns of motivational orientation, much like other affective states (e.g., anxiety and avoidance or anger and approach; Blascovich, 2008[13]). In performance situations, a challenge state is generally associated with approach motivation and a threat state with avoidance motivation (e.g., Behnke et al., 2022[7]; Jamieson and Mendes, 2016[58]; Jamieson et al., 2014[60], 2022[56]). Moreover, whereas a challenge state is typically associated with positive behavioral and performance outcomes (see Behnke and Kaczmarek, 2018[8]; Hase et al., 2019[50], for reviews), a threat state often impairs decision-making in the short-term and in the long-term is associated with accelerated “brain aging,” cognitive decline, poorer mental health (e.g., more symptoms of anxiety and depression) and cardiovascular disease (Jefferson et al., 2010[61]; McLoughlin et al., 2024[79]; Matthews et al., 1997[75]; Yeager et al., 2022[135]).

### BPSM applications

The application of the BPSM to different research settings has led to support for its conceptualization of psychophysiological stress responses in motivated performance settings as well as the predictive utility of CAT responses for understanding performance outcomes. Precisely, a systematic review (Hase et al., 2019[50]) and a meta-analysis (Behnke and Kaczmarek, 2018[8]) both found a challenge state to generally result in better performance than a threat state. The reviewed studies took place in diverse performance contexts, including sport, medicine, academia, and aviation (e.g., Blascovich et al., 2004[15]; Moore et al., 2013[91], 2014[88]; Seery et al., 2010[113]; Vine et al., 2015[132]). They relied on various research designs and included both CAT cognitive evaluations and cardiovascular responses. However, there were some inconsistencies in the reviewed research (e.g., the inclusion of studies until 2017 or 2018 and the differences between included studies), and the only meta-analysis to date restricted its focus to CAT cardiovascular responses and did not synthesize results relating to cognitive CAT evaluations and performance outcomes (Behnke and Kaczmarek, 2018[8]). Moreover, societal changes tied to the COVID-19 pandemic and its aftermath warrant a new assessment of how psychophysiological stress responses map onto performance. Notably, stress processes related to anxiety, burnout, and political polarization have contributed to the growing “mental health pandemic” in the wake of COVID-19 (Daniali et al., 2023[32]; Sacco, 2023[102]). Thus, we argue that a new meta-analysis of both CAT cognitive evaluations and cardiovascular responses is much needed, especially given the numerous new studies that have been published since the last review by Hase and colleagues published in 2019[50] (e.g., Behnke et al., 2022[7], 2024[10]; Jewiss et al., 2023[63], 2024[62]; Sharpe et al., 2024[115]).

These new studies used established and novel approaches to examine CAT states from a BPSM perspective. For example, one study used an observational study to extend CAT research into the esports context, finding that CAT cognitive evaluations predicted goal ratio in the soccer game FIFA 19 (Behnke et al., 2022[7]). Another study involved naturalistic observation of cardiovascular CAT responses in a pressurized performance context (i.e., competitive football or soccer match), which were then used to divide an elite-level sample into three cardiovascular reactivity groups: challenge, threat, and blunted reactivity (Dixon et al., 2020[34]). The blunted reactivity group underperformed compared to the other two groups, which highlights the importance of examining boundary conditions in BPSM-based CAT research (Hase et al., 2020[47]). Finally, replication studies have been conducted, with Jewiss et al. (2023[63]) finding similar benefits (i.e., of a challenge over a threat state) on cricket batting performance and Jewiss et al. (2024[62]) failing to replicate the seminal findings of Blascovich et al. (2004[15]) when connecting CAT states with season-long cricket batting performance. 

Beyond these observational studies, several experiments have attempted to manipulate CAT states directly or indirectly and thereby improve performance. Indeed, in one study (McGreary et al., 2020[76]), elite-level junior cricket batsmen were manipulated into a challenge or a threat state using established CAT instructions (from Moore et al., 2012[90]). In the challenge group, all participants completed a subsequent cricket task successfully, whereas only one participant in the threat group completed the task successfully (McGreary et al., 2020[76]). Another experiment tested a nutritional supplement-based manipulation of CAT states using the catecholamine precursor l-tyrosine (Hase et al., 2019[48]). Although acute l-tyrosine intake did not affect CAT states directly, it benefited individuals with relatively more threatened cardiovascular responses in maintaining optimal performance. Another study, which was a preregistered report, introduced a synergistic mindsets intervention inspired by past work (Yeager et al., 2022[135]) to change esports players' CAT cognitive evaluations and cardiovascular responses (Behnke et al., 2024[10]). After two weeks of training and applying the intervention, the authors did not find a relationship between CAT states and performance, which is notable due to the methodological strength of this study (i.e., among others, a relatively large sample size and preregistration). 

In addition to the ongoing research and application of the BPSM in various performance contexts (e.g., education; Jamieson et al., 2021[57]), the theoretical interest in understanding CAT states has spurred the development of new models that build on the BPSM. For instance, Meijen et al. (2020[80]) proposed a revised conceptual model that built on the theory of challenge and threat states (TCTSA) by Jones et al. (2009[64]) and addressed CAT states in athletes (e.g., proposing new physiological markers such as neuropeptide Y and oxytocin). Similarly, Uphill et al. (2019[126]) critically reviewed existing literature and suggested an alternative framework for understanding CAT states (e.g., that performers can be challenged, threatened, both, or neither in motivated performance situations). Finally, Hase et al. (2020[47]) proposed adding blunted cardiovascular reactivity to the model to represent deficient task engagement and, more generally, motivational disengagement due to threat states. While these new models offer valuable theoretical advancements, they do not directly provide additional insights into the specific cognitive and cardiovascular pathways examined in the present review and are, therefore, not considered in detail for this meta-analysis. 

Given the continued research interest in the BPSM to explain performance outcomes (e.g., Kelley et al., 2019[65]), this meta-analytic review synthesizes the research published since 2017 to update the results following an uncertain time for society (e.g., due to COVID-19) and to build upon and scrutinize the conclusions of the previous reviews (i.e., Behnke and Kaczmarek, 2018[8]; Hase et al., 2019[50]). Specifically, it aims to clarify the relationship between different CAT cognitive evaluations and cardiovascular responses and various performance outcomes across different research designs (e.g., experimental to observational). The focal hypothesis is that a challenge state would be associated with better performance outcomes than a threat state. We also performed exploratory analyses to test for potential moderators of the hypothesized effect, as well as examine associations among different measures of CAT (cognitive and physiological) to better understand the coherence among the different measure modalities. In doing so, this novel review provided information about how methodological choices (e.g., measuring cardiovascular responses before vs. during performance or manipulating vs. observing CAT states) affect the CAT-performance relationship.

## Method

### Study selection

**Figure 1:**
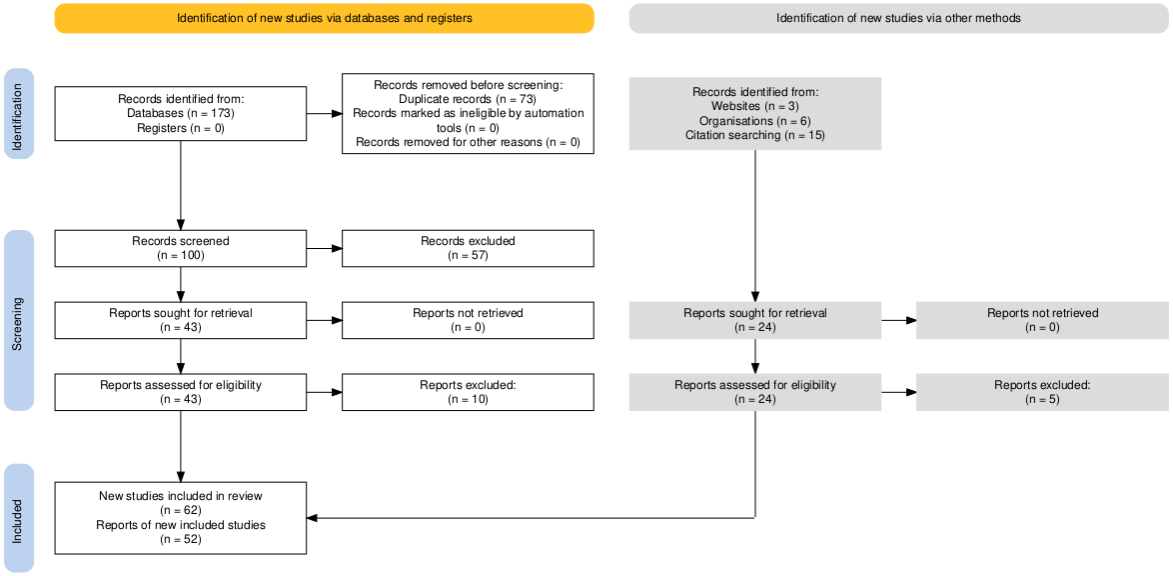
PRISMA flowchart

We used the PRISMA2020 application to graphically summarize the study selection process in a PRISMA flowchart (Haddaway et al., 2022[45]; Figure 1(Fig. 1)). After the initial literature search (conducted in March 2024), the primary study selection process included three steps, which followed the procedure of Hase et al. (2019[50]), that is: screening based on title, followed by abstract, and finally full text. Three independent assessors (AH, MN, and MB) conducted the literature search, compared their results, and resolved any discrepancies by discussion (initial agreement in steps 1, 2, and 3 = 50.0 %, 56.8 %, and 81.7 %, respectively). The included databases were: MedLine, PsycINFO (combined in one search), SPORTDiscus, and Web of Science (in separate searches). The search terms were (“challenge and threat” AND “performance”). We then scanned the reference lists of the included articles for other articles that were potentially not picked up in the systematic search process. Furthermore, we included three studies (Moore et al., 2018[92]; Sammy et al., 2017[103]; Scheepers, 2017[104]) from a previous systematic review (Hase et al., 2019[50]), as the previous meta-analysis (Behnke and Kaczmarek, 2018[8]) did not include these studies. Finally, we conducted a forward search to evaluate studies that cited those already identified as relevant to this project, by using the “cited by” option in Google Scholar. We excluded studies that: (1) did not measure performance outcomes objectively; (2) did not measure CAT cognitive evaluations (i.e., demands and resources) and/or cardiovascular indexes (i.e., CO, TPR, CTI); and (3) did not provide the effect sizes for the associations between CAT states and performance outcomes. We did not pre-register this meta-analysis.

### Risk of bias analysis

To assess methodological quality, we used the Risk of Bias Assessment Tool for Nonrandomized Studies (RoBANS 2; Kim et al., 2013[68]) and the revised Cochrane tool for assessing risk of bias in randomized trials (Sterne et al., 2019[121]). We used the RoBANS 2 tool for the risk of bias assessment in observational studies that provided tests of associations between CAT states (cognitive evaluations and/or cardiovascular responses) and performance outcomes. The RoBANS 2 tool provides a domain-specific assessment of six risk of bias domains: participant selection, confounding variables, measurement of exposure, blinding of outcome assessment, incomplete outcome data, and selective reporting. Possible ratings are low, unclear, or high risk of bias for each domain and the overall study assessment.

The only exceptions were studies that randomized participants into challenge versus threat groups, intentionally manipulated CAT states (e.g., with a set of instructions or nutritional supplement), and subsequently confirmed the successful manipulation of CAT states with a check using established cognitive or physiological CAT measures (e.g., Behnke et al., 2020[9]). For these randomized studies, we used the revised Cochrane tool for assessing the risk of bias (ROB-2; Sterne et al., 2019[121]). The ROB-2 comprises evaluations of bias in five distinct domains: bias due to issues with the randomization process, deviations from intended interventions, missing outcome data, outcome measurement, and selection of the reported result. Possible ratings are “low risk of bias”, “some concerns”, and “high risk of bias”. In both the ROBANS 2 and the ROB-2 tool, the highest individual domain rating also produced the overall risk of bias rating. To visualize the results of the risk of bias assessments, we created traffic light plots using *robvis *R package (McGuinness and Higgins, 2021[78]). 

**Coding*.*** Based on our previous reviews of the CAT literature (Behnke and Kaczmarek, 2018[8]; Hase et al., 2019[50]), the following potential moderators were coded: (1) the timing of cardiovascular measures (before/during performance); (2) age of the sampled participants (sample mean in years); (3) gender of the sampled participants (percentage of females), (4) publication year, and (5) risk of bias. 

When coding for the correlations with performance outcomes, we multiplied the effects by -1 for the types of tasks where lower numbers indicated better performance (e.g., time, error); thus, for all correlations, the higher the positive correlation coefficient, the more strongly the CAT cognitive evaluations and cardiovascular responses correlated with better performance. Similarly, for the CAT cognitive evaluations, we multiplied the effects related to evaluated demands by -1; thus, for all CAT cognitive evaluations, the higher the positive correlation coefficient, the more strongly the challenge evaluations (i.e., personal coping resources match or exceed situational demands) correlated with better performance.

**Effect size extraction*.*** For the meta-analysis, we used the correlation coefficients between CAT cognitive evaluations and/or cardiovascular responses and performance outcomes as a measure of the effect size. For most studies, the authors reported the correlation coefficients. For studies reporting other metrics (e.g., between-group differences), we sent requests to authors to provide the correlation coefficients. Of the 47 authors we contacted, 37 responded to our inquiry (79 %), and of these, 29 sent us the requested data (78 %). No authors denied us access to the requested data. The list of authors who provided and did not provide the data is to be found in Supplementary data (“*Data.xlsx* file, “*mails*” sheet).

### Meta-analytic procedures

We conducted meta-analyses using R (R Core Team, 2023[100]) and the *metafor* package (Viechtbauer, 2010[129]), adhering to established guidelines (Assink and Wibbelink, 2016[5]; Viechtbauer, 2010[129]). Given the anticipated high heterogeneity of effects (as in Behnke and Kaczmarek, 2018[8]), we used a random-effects model. Random-effects models are recommended for meta-analyses because they facilitate the generalization of corrected effect sizes to the broader population (Field and Gillett, 2010[41]; Schmidt and Hunter, 2015[107]). Additionally, simulation studies have shown that using separate three-level models for different outcome types is the most reliable method for obtaining accurate standard error estimates (Fernández-Castilla et al., 2021[39]). Following prior research (e.g., Behnke et al., 2022[7]; Behnke et al., 2023[11]), we applied restricted maximum likelihood estimation to compute pooled mean effect sizes. Several of the included studies reported multiple effect sizes. To address the dependency among these effect sizes, we used a three-level meta-analytic approach (Assink and Wibbelink, 2016[5]; Viechtbauer, 2010[129]). This method accounts for three distinct sources of variance: (1) between-study variance, which captures differences in effect sizes across studies (level three); (2) within-study variance, arising from multiple effect sizes reported in the same study (e.g., using two cardiovascular measures; level two); and (3) sampling variance of the extracted effect sizes (level one) (Cheung, 2014[29]; Van den Noortgate et al., 2013[127]).

**Magnitude and consistency of the effects*.*** We aimed to examine a pooled mean relationship between CAT cognitive evaluations and/or cardiovascular responses and performance outcomes. We interpret the results considering two parameters, namely, relationship magnitude (size of the mean effect sizes) and consistency (no/low heterogeneity vs. high heterogeneity). Heterogeneous effect sizes may indicate that: (a) the average relationship is not consistent for CAT states and performance outcomes; (b) the average relationship between CAT states and performance outcomes is moderated by different types of characteristics (e.g., timing); or (c) the size of the effect reflects real, contextual changes in the relationship between CAT states and performance outcomes. 

We assessed the homogeneity of the calculated mean effect sizes using *I*^2^-statistic. A rejection of the null hypothesis suggests the presence of methodological or population differences that contribute to variability between studies. Additionally, the *I^2^*-statistic facilitates comparisons across meta-analyses (Higgins et al., 2003[51]). It measures the proportion of variance attributable to within-study effects (level two of the meta-analytic model) and between-study effects (level three). The total *I^2^* value represents the overall variability, where 0 % indicates no dispersion and higher values signify varying degrees of heterogeneity: 0-40 % might be negligible, 30-60 % may indicate moderate heterogeneity, 50-90 % may reflect substantial heterogeneity, and 75-100 % might represent considerable heterogeneity (Higgins et al., 2019 53). To test the significance of variance components at levels two and three, we conducted separate one-tailed log-likelihood ratio tests. These compared the deviance of the full model to reduced models that excluded one variance parameter. We then calculated the prediction intervals for the mean correlations to address the heterogeneity of the pooled effect sizes (Borenstein et al., 2017[18]; Higgins et al., 2009[52]; Riley et al., 2011[101]). The prediction intervals present where the true effect size in 95 % of all comparable populations would fall. We intended to present the pooled effect sizes of the relationship between CAT states (cognitive evaluations and/or cardiovascular responses) and performance outcomes, as well as the prediction intervals, as correlation coefficients.

**Publication bias.** We conducted five publication bias analyses to determine whether null or weak results might have been systematically underrepresented in the literature (Schmidt and Hunter, 2015[107]). Investigating potential bias in synthesized effect sizes is crucial, as studies reporting non-significant or negative findings are less likely to appear in peer-reviewed journals (Borenstein et al., 2009[17]). Our strategy included five complementary methods: (1) a visual inspection of the funnel plot, (2) a rank correlation test, (3) Egger's regression test (Egger et al., 1997[38]), (4) the trim-and-fill analysis (Duval and Tweedie, 2000[37]), and (5) a moderator test in which the publication year of studies was tested as a moderator of the overall effect. This approach aligns with similar strategies employed in previous research (e.g., Assink et al., 2018[3], 2019[4]; Behnke et al., 2022[7], 2023[11]). In our strategy, we used some standard methods for two-level meta-analysis because their counterparts for three-level meta-analyses have not yet been developed and tested (Fernández-Castilla et al., 2020[40]).

First, we visually inspected a funnel plot in which effect sizes are plotted against their standard error around an estimated summary effect (Egger et al., 1997[38]). In contrast to large studies, studies using small samples tend to produce effect sizes of different magnitudes due to increased variability in their sampling errors. Thus, effect sizes from smaller studies are expected to scatter widely at the bottom of the funnel plot. In contrast, effect sizes from larger studies are expected to be more concentrated at the top of the plot. As null or weak results are likely to be systematically suppressed from publication, an asymmetric distribution of effect sizes may be expected in the sense that effect sizes may be missing at the (bottom) left of the estimated summary effect in the funnel plot. In contrast, a symmetric effect size distribution with effect sizes equally distributed to the left and right of a summary effect would suggest the absence of publication bias. Second and third, we applied an adapted version of Egger's regression test and Begg and Mazumdar's rank-order correlation test (Assink et al., 2018[3], 2019[4]). In the adapted Egger's test, we regressed effect sizes on their standard errors within a three-level meta-analytic framework, accounting for the dependency among effect sizes derived from the same primary studies. Unlike the classical Egger's test, this adaptation addresses effect size dependency. In Egger's regression test, a significant slope suggests the presence of publication bias, while in Begg and Mazumdar's rank-order test, a significant rank association indicates bias (Assink et al., 2018[3], 2019[4]).

Fourth, we used the trim-and-fill method (Duval and Tweedie, 2000[37]) to examine bias-corrected effect sizes. This method addresses funnel plot asymmetry by imputing effect sizes from "missing" studies to restore symmetry in the plot. Fifth, we tested for a potential "decline effect" (Schooler, 2011[110]) by examining whether effect sizes decreased over time. This was done by regressing the summary effect on the publication year of the primary studies. A tendency for effect sizes to diminish over time may reflect what Ioannidis (2005[54]) described as the law of initial results. To extend the range of publication years in this analysis, we included data from previous meta-analyses (Behnke and Kaczmarek, 2018[8]).

**Moderator analyses*.*** Finally, to examine potential moderators that may influence the relationship between CAT states and performance outcomes, we ran five types of separate moderator analyses. We tested whether the findings differ for different groups of studies: for example, studies that measured CAT cardiovascular responses before vs. during performance and studies with low, unclear, or high risk of bias. Furthermore, we explored whether the age of the sampled participants influenced the size of the effect sizes (sample mean in years), gender of the sampled participants (percentage of females), and publication year. We examined whether the relationship between CAT states and performance outcomes varied across (categories of) the factor by interpreting the results of an omnibus test. A significant *F* value in this test indicates differences across (categories of) the factor, serving as a potential moderator. To control for Type I error due to multiple comparisons, such as testing five different moderators within the same dataset - a common issue in meta-analyses (Cafri et al., 2010[24]) - we applied Bonferroni correction to adjust probability values (Abdi, 2007[1]). Following our previous meta-analyses (Behnke et al., 2022[7], 2023[11]), we considered the mean effect of a moderator category only when at least three studies supported it. 

## Results

### Descriptive analyses 

We identified 72 records in the first search (PsycINFO and Medline), 23 records in the second search (Sportdiscus, 22 duplicates), and 78 in the third search (Web of Science, 51 duplicates). Thus, 100 unique titles were evaluated in the first review step. Based on the initial evaluation, we selected 95 titles for inclusion in step 1 (50.0 % initial agreement between the three reviewers), 81 abstracts in step 2 (56.8 % agreement), and 43 full texts in step 3 (81.7 % agreement). We added three additional articles based on a reference list search, three studies from 2017, six sets of unpublished results or published articles sent by contacted authors, and 12 reports identified by forward search. Thus, the final number of articles coded for the meta-analysis was 67. 

After excluding papers that did not present the data needed for meta-analysis, we included 52 articles with 62 studies (Table 1(Tab. 1); References in Table 1: Arthur et al., 2019[2]; Baumgartner and Schneider, 2023[6]; Behnke et al., 2020[9], 2022[7], 2024[10]; Bosshard et al., 2023[19]; Brimmell et al., 2019[20]; Cabral et al., 2024[23]; Carenzo et al., 2020[25]; Conlon et al., 2022[30]; Crowe et al., 2020[31]; Dixon et al., 2020[34]; Gurera and Isaacowitz, 2022[43]; Hangen et al., 2019[46]; Hase et al., 2019[48][49], in preparation a, in preparation b; Jamieson et al., 2021[57], 2022[56]; Jewiss et al., 2023[63], 2024[62]; Journault et al., in preparation; Khalaf et al., 2020[67]; Laurin and Pellet, 2023[69]; Lee et al., 2019[71]; Malkoc et al., 2023[72]; Mansell, 2023[73]; Marr et al., 2021[74]; Miller et al., 2021[84][83]; Moe and Putwain, 2020[86]; Moore et al., 2018[92]; Mosley et al., 2018[93]; Mulvenna et al., 2023[94]; O'Brien et al., 2020[95]; Petzel and Casad, 2022[97]; Sammy et al., 2017[103]; Scheepers, 2017[104]; Scheepers and Keller, 2022[105]; Schickel et al., 2023[106]; Sharpe et al., 2024[115][114]; Simms, 2022[116]; Slater et al., 2018[118]; Smith et al., 2022[119]; Snijdewint and Scheepers, 2023[120]; Thornton et al., 2021[122]; Trotman et al., 2018[124]; Turner et al., 2021[125]; van Gog et al., 2024[128]; Wood et al., 2018[134]), presenting 336 effect sizes obtained from 7,418 participants (14,220 performances) with the mean ages ranging from 13.26 to 72.00 years (*M* = 21.70 years, *SD *= 7.66; Figure 1(Fig. 1)). Sample sizes ranged from 10 to 499, with a mean sample size of 119.65 participants (*SD* = 111.34). More than half of the participants (51 %) were male. Most samples contained both genders, but eight samples were all-male (Behnke et al., 2020[9], 2022[7], 2024[10]; Dixon et al., 2020[34]; Jewiss et al., 2023[63], 2024[62]; Sharpe et al., 2024[114]; Trotman et al., 2018[124]), and three samples were all-female (Petzel and Casad, 2022[97]; Scheepers, 2017[104]; Turner et al., 2021[125]). The included studies were published between 2017 and 2024 (median publication year = 2022). 

The predominantly studied type of performance was math (*n* = 9 studies; 15 %), followed by esports (8; 13 %), throwing (5; 8 %), academic exams (5; 8 %), darts (4; 6 %), problem-solving tasks (4; 6 %), soccer (3; 5 %), golf (3; 5 %), indoor cycling (2; 3 %), presentation or speech performance (2; 3 %), cricket (2; 3 %), memory task (2; 3 %), and communication task, cooperation computer game, medical test, muscular endurance, netball, pattern recognition task, psychomotor vigilance task, running, shooting, tennis, Tetris, trampoline, and visual search task (all 1; < 2 %). The most frequent cardiovascular measure was Challenge Threat Index (CTI) (73 effect sizes; 36 %), followed by cardiac output (CO) (68 effect sizes; 34 %), and total peripheral resistance (TPR) (61 effect sizes; 30 %). The most frequent CAT cognitive evaluation measure was different adaptations of the two-item cognitive appraisal ratio (Tomaka et al., 1993[123]) (n = 27 studies; 60 %), followed by the four-item measure developed by Mendes et al. (2007[81]) (4; 9 %), the two-item direct questions of challenge and threat (3; 7 %), the two-item appraisal measure developed by Skinner and Brewer (2002[117]) (2; 4 %), the eight-item version of the challenge and threat construal measure (McGregor and Elliot, 2002[77]) (2; 4 %), six affect adjectives (Folkman and Lazarus, 1985[42]), Challenge-Threat Scale (Drach-Zahavy and Erez, 2002[36]), four items created by Putwain et al. (2015[99], 2016[98]), a single item about being challenged-threatened (Thornton et al., 2021[122]), a single item related to resources (Lee et al., 2019[71]), and 11-item appraisal measure developed by Mendes et al. (2007[81]), and the Stressor Appraisal Scale (Schneider, 2008[108]; all 1; 2 %). 

### Risk of bias in individual studies

**Figure 2:**
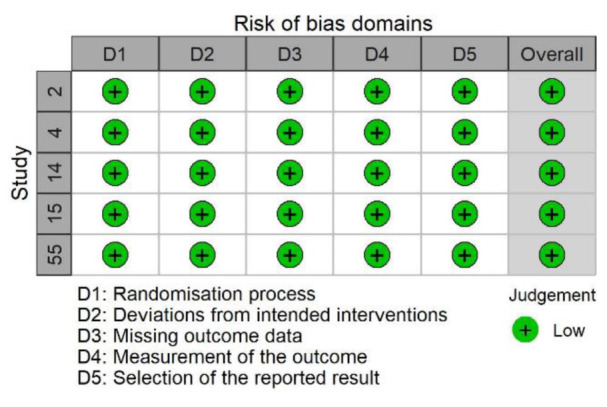
Traffic light plot for the ROB-2 assessment results. Note: Study ID relates to Table 1

**Figure 3:**
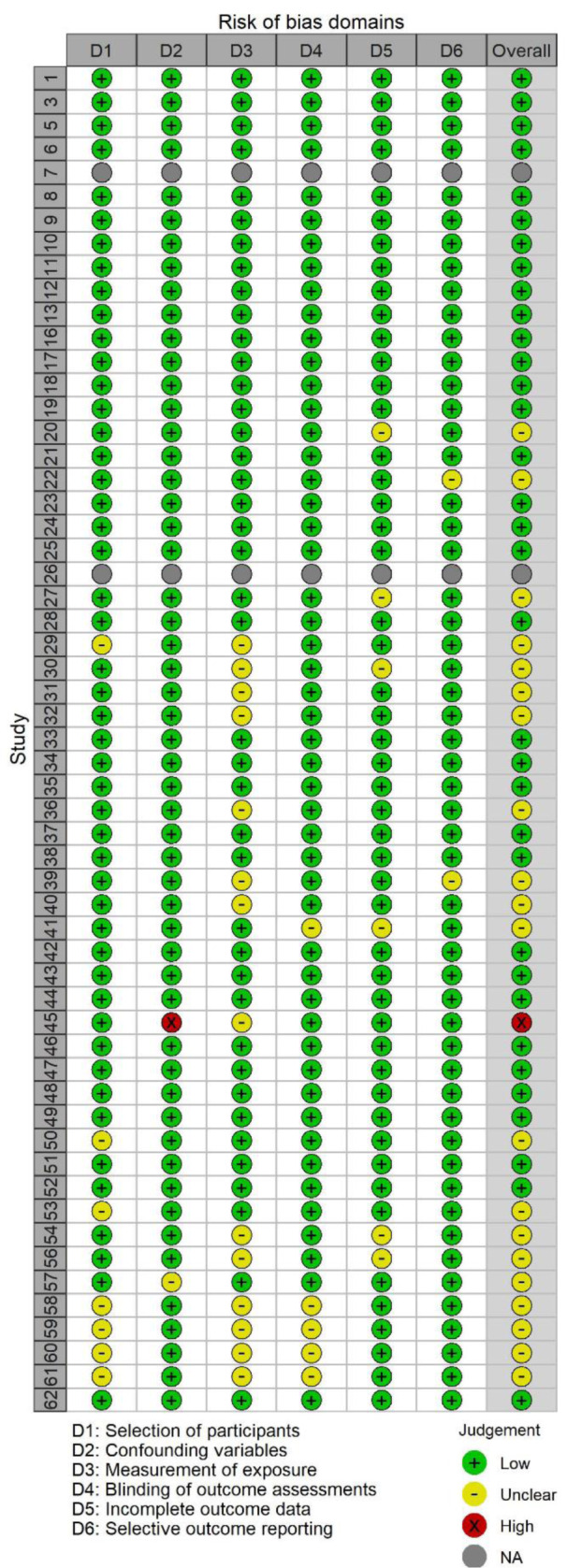
Traffic light plot for the ROBANS assessment results. Note: Study ID relates to Table1

We present risk of bias traffic light plots in Figure 2(Fig. 2) and Figure 3(Fig. 3). Most of the included studies (65.0 %) featured “low” study-level risk of bias ratings. Of the five direct-experimental studies, five (100 %) received a low risk of bias rating. Of the 57 studies that reported a correlational or indirect experimental result between CAT states and performance, 34 (61.8 %) received a low, 20 (36.4 %) an unclear, and one (1.8 %) a high risk of bias rating. We did not assess two unpublished studies. An unclear or high risk of bias rating was often associated with deviations from the typical BPSM-based measurement of CAT states and missing outcome data exceeding 20 %. The average initial interrater agreement across risk of bias domain ratings was 92.1 %.

### Relationship between CAT states and performance outcomes

**Figure 4:**
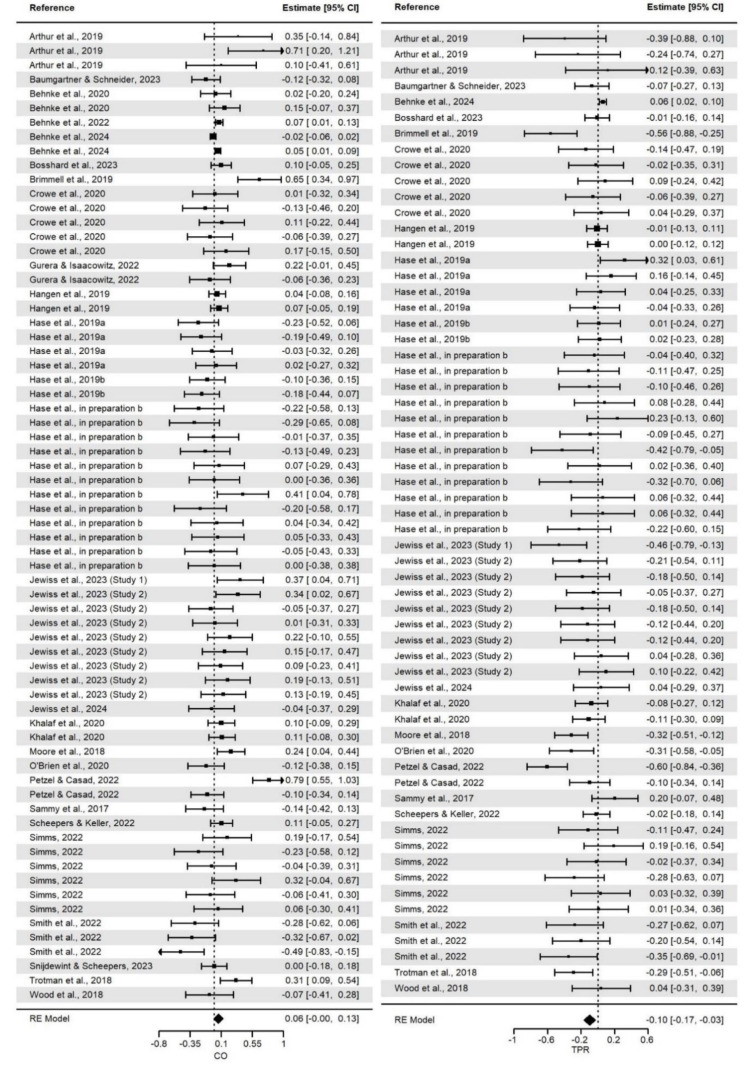
Forest plots for effect sizes for associations between cardiac output, total peripheral resistance and performance outcomes. *Note. *CO = Cardiac Output. TPR = Total Peripheral Resistance. The square boxes represent effect sizes and sample sizes (the larger the box, the larger the sample size, contributed more to the total effect size) in each study. The lines represent 95% confidence intervals. The diamonds represent the pooled effect size and the 95% confidence intervals.

**Figure 5:**
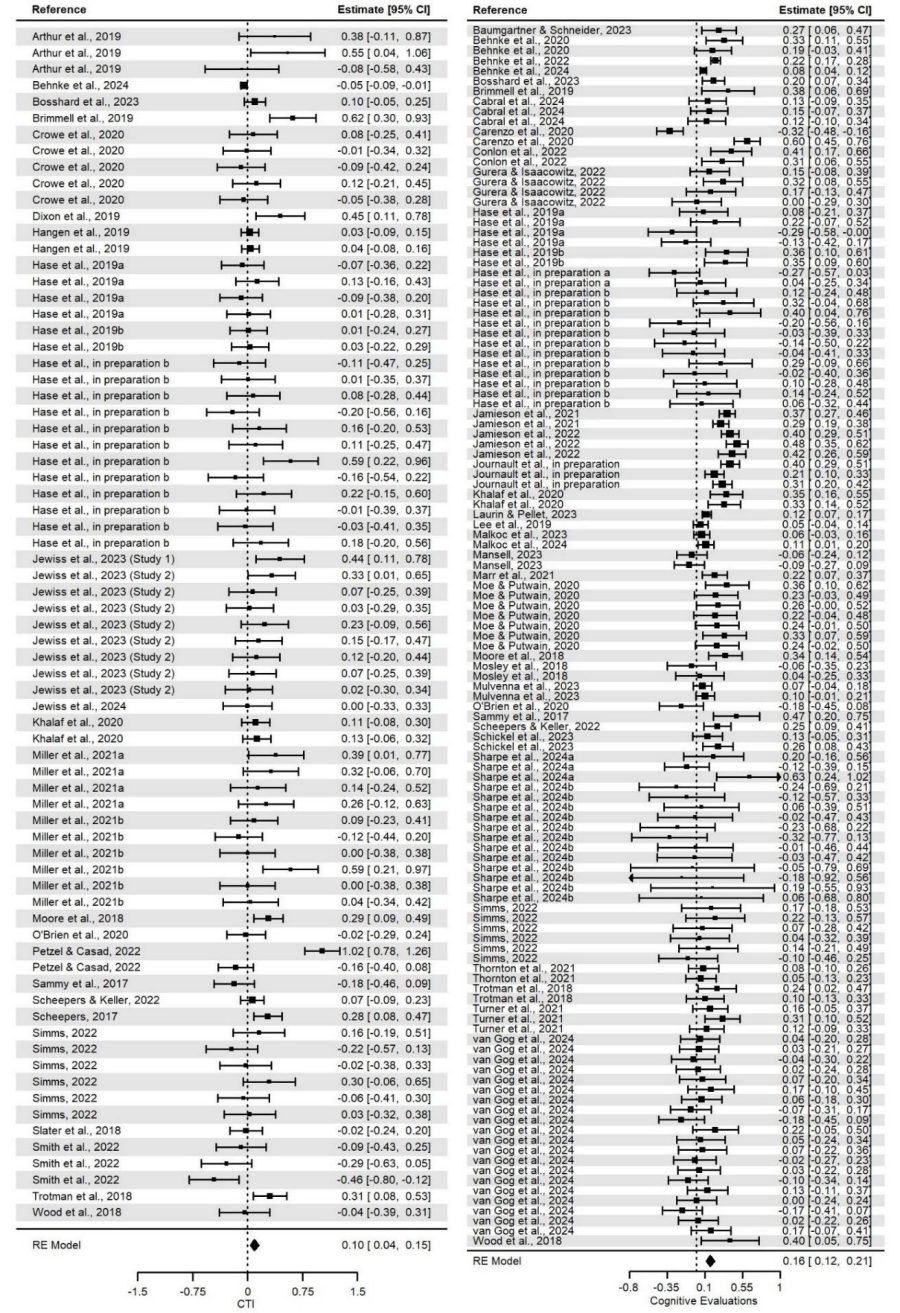
Forest plot for effect sizes for associations between challenge threat index, cognitive evaluations, and performance outcomes *Note. *CTI = Challenge Threat Index. The square boxes represent effect sizes and sample sizes (the larger the box, the larger the sample size, contributed more to the total effect size) in each study. The lines represent 95% confidence intervals. The diamond represents the pooled effect size and the 95% confidence intervals.

Overall, we found expected (positive or negative) correlations for the relationship between CAT cognitive evaluations and/or cardiovascular responses and the performance outcomes (Table 2(Tab. 2)). However, the relationship between CO reactivity and performance outcomes was not statistically significant. All mean effect sizes for the relationships should be interpreted as small. The distributions of effect sizes for the given measure are presented on Figure 4(Fig. 4) and 5(Fig. 5) (References in Figure 4 and 5: Arthur et al., 2019[2]; Baumgartner and Schneider, 2023[6]; Behnke et al., 2020[9], 2022[7], 2024[10]; Bosshard et al., 2023[19]; Brimmell et al., 2019[20]; Cabral et al., 2024[23]; Carenzo et al., 2020[25]; Conlon et al., 2022[30]; Crowe et al., 2020[31]; Dixon et al., 2020[34]; Gurera and Isaacowitz, 2022[43]; Hangen et al., 2019[46]; Hase et al., 2019[48][49], in preparation a, in preparation b; Jamieson et al., 2021[57], 2022[56]; Jewiss et al., 2023[63], 2024[62]; Journault et al., in preparation; Khalaf et al., 2020[67]; Laurin and Pellet, 2023[69]; Lee et al., 2019[71]; Malkoc et al., 2023[72]; Mansell, 2023[73]; Marr et al., 2021[74]; Miller et al., 2021[84][83]; Moe and Putwain, 2020[86]; Moore et al., 2018[92]; Mosley et al., 2018[93]; Mulvenna et al., 2023[94]; O'Brien et al., 2020[95]; Petzel and Casad, 2022[97]; Sammy et al., 2017[103]; Scheepers, 2017[104]; Scheepers and Keller, 2022[105]; Schickel et al., 2023[106]; Sharpe et al., 2024[115][114]; Simms, 2022[116]; Slater et al., 2018[118]; Smith et al., 2022[119]; Snijdewint and Scheepers, 2023[120]; Thornton et al., 2021[122]; Trotman et al., 2018[124]; Turner et al., 2021[125]; van Gog et al., 2024[128]; Wood et al., 2018[134]; for more detailed figures, see Supplementary information, “*CAT_Meta2024.pdf*” file).

Furthermore, as for heterogeneity in effect sizes in the models, we did not find significant within-study (level 2) or between-study (level 3) variance. A breakdown of the total variance into the variance distributed at the three levels of the model revealed that almost all could be attributed to sampling variance. Thus, we rejected the null hypothesis of effect size homogeneity and found that the true effect size was heterogeneous and was likely to vary from effect size to effect size. This indicates that the effect sizes should not be treated as estimates of one common effect size, and thus, moderator analyses are justified to search for variables that can explain the heterogeneity of the relationship between CAT states (cognitive evaluations and/or cardiovascular responses) and performance outcomes.

### Publication bias

We investigated outliers by calculating studentized residuals, which identify effect sizes that disproportionately contribute to the overall heterogeneity and the results. We identified two effect sizes for CO and TPR that stood out with particularly large values (both in Petzel and Casad, 2022[97]), with all *Zs* > 3.00. We double-checked the effects and decided to keep them as they were clearly presented in the manuscript.

**Figure 6:**
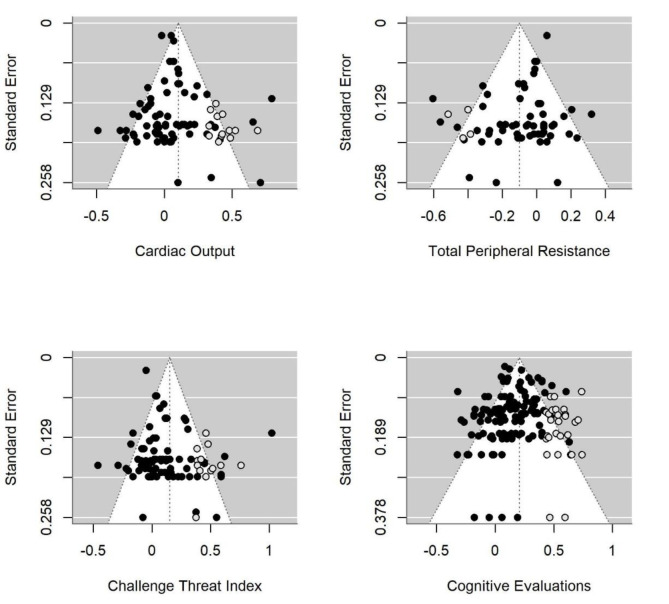
Funnel plots for associations between challenge and threat cardiovascular responses and cognitive evaluations and performance outcomes *Note.* Black dots indicate studies included in the meta-analysis. White dots indicate studies “filled” based on the trim-and-fill method estimation.

As for the publication bias assessment results, we found mixed evidence that the distribution of effect sizes was asymmetrical. First, for CO, TPR, and CTI, the trim-and-fill analysis imputed "missing effects", for the non-multilevel models (Figure 6(Fig. 6)). The analysis showed that our results might be underestimated due to the missing values and that the 'real' mean effect sizes might be bigger. The results of the rank-order correlation test and adapted Egger's regression test did not suggest an asymmetrical distribution of effects (except for CTI; see Table 2(Tab. 2)). Furthermore, we found no association between publication year and strength of CAT-performance relationship (for both CAT cardiovascular responses and cognitive evaluations), indicating that the magnitude of effect sizes did not decrease over time, meaning that more recent studies showed similar effect sizes. In sum, only two out of the five bias assessment methods applied suggested the presence of publication bias in the synthesized studies. Therefore, the evidence for publication bias affecting the results of this meta-analysis is relatively limited.

### Moderator analyses

The moderator analyses showed that under most conditions, the relationship between CAT states (cognitive evaluations and/or cardiovascular responses) and performance outcomes was not affected by most moderators. Table 3(Tab. 3) presents the results of the omnibus tests of the moderator analyses. After adjusting for Bonferroni correction, the significant moderators were the study design for the relationship between CTI and performance outcomes, and the risk of bias category for the relationship between TPR, CTI, and cognitive evaluations and performance outcomes. When CTI was measured before the performance, the association with performance outcomes was weaker, *r* = .08, 95 % CI [.02, .14], than when measured during performance, *r* = .16, 95 % CI [.03, .29]. Furthermore, when the risk of bias was low, the association between TPR and performance outcomes was weaker, *r* = -.05, 95 % CI [-.12, .01], than when the Risk of Bias was unclear, *r* = -.25, 95 % CI [-.38, .13]. For CAT cognitive evaluations, when the risk of bias was low, the association with performance outcomes was stronger, *r* = .18, 95 % CI [.12, .24], than when the Risk of Bias was unclear, *r* = .14, 95 % CI [.05, .21]. Finally, the difference between groups of studies for CTI (*r* = .10, vs. *r* = .10) was not interpretable. 

### Synthesis of evidence not included in the meta-analysis

Some studies could not be included in the meta-analysis for different reasons, like a lack of reported effect sizes and/or data for the computation of effect sizes or authors' nonresponses to our data requests. Overall, we identified seven studies that could not be included in the meta-analysis for these reasons. Of these studies, some still reported results relevant to a qualitative narrative review of the CAT-performance relationship. Indeed, two studies reported null results (Chadha et al., 2023[26]; Dixon et al., 2020[34]; study 2), although Dixon and colleagues found a significant association between cardiovascular CAT and performance when considering player-based performance ratings. In an experimental comparison of heat and moderate temperatures in a cycling ergometer endurance test, Donnan et al. (2022[35]) found that performance declined more in the hot condition, such that it was lower in the second half of the task. Moreover, the hot condition featured cognitive CAT evaluations more consistent with a threat state. In an experimental study, McGreary et al. (2020[76]) found that nine out of 10 participants in a threat group did not successfully complete a cricket task, whereas 10 out of 10 participants in the challenge group successfully completed the task. Although the finding could not be included in the meta-analysis results, a chi-squared test showed that successful performance was significantly more frequent in the challenge than threat group, X(1) = 12.93, *p* < .001. One study found that loss (vs. gain) framing led to increased threat cognitive evaluations (measured separately from challenge cognitive evaluations, which were unaffected by the manipulation) and, in turn, to worse working memory performance (Chen and Qu, 2023[28]). Interestingly, no study reported a significant association contrary to the BPSM's predictions (e.g., a threat state linked to better performance outcomes than a challenge state). Finally, one article described a study protocol which had not yet led to collected data that could have been included in the meta-analysis (Guyon et al., 2020[44]).

## Discussion

This systematic review and meta-analysis examined the relationship between CAT cognitive evaluations and cardiovascular responses, as conceptualized by the BPSM (Blascovich, 2008[13]; Seery, 2011[111]), and performance outcomes to compare the recent empirical research with that reviewed by Behnke and Kaczmarek (2018[8]) and Hase et al. (2019[50]). Consistent with the previous meta-analysis (Behnke and Kaczmarek, 2018[8]) and systematic review (Hase et al., 2019[50]), the present analysis established the superiority of a challenge state over a threat state on performance outcomes when using CAT cognitive evaluations (e.g., demand resource evaluation score) and cardiovascular responses (i.e., TPR and CTI). However, the meta-analytic effects for CO did not reach statistical significance. These results, by and large, support the predictions of the BPSM and provide a relatively reliable estimate of typical effect sizes in BPSM-based CAT research (i.e., small but robust effects).

Most of the included studies featured a low risk of bias. However, some studies were flagged as unclear or high risk owing to issues such as deviations from the CAT measures typically used in research using a BPSM-based perspective (e.g., Schickel et al., 2023[106]) or having more than 20 % missing CAT or performance outcome data in the relevant analyses (e.g., Hase et al., in prep. (b); Malkoc et al., 2023[72]; Trotman et al., 2018[124]). We therefore recommend that CAT researchers conduct more preregistered studies, publish the study protocols with pre-planned analyses, and publish the raw open-access data in advance to prevent publication bias and methodology-related replicability issues (see Behnke et al., 2024[10] for an example). However, it is worth mentioning that some of the recent studies have already implemented these recommendations; that is, they were preregistered and involved open data sharing (Bosshard et al., 2023[19]; Guyon et al., 2020[44]; Yeager et al., 2022[135]) or tried to replicate prior research (e.g., Jewiss et al., 2023[63], 2024[62]). Furthermore, we recommend that researchers include correlation tables in their manuscripts or supplementary materials and raw data that would allow researchers to extract data for future meta-analyses. The raw data would help researchers to run analyses using innovative approaches, including accounting for non-responders (i.e., a blunted cardiovascular response; Hase et al., 2020[47]) or using other cardiovascular measures (e.g., stroke volume instead of CO; Yeager et al., 2022[135]). 

We also examined publication bias and moderator analysis results for meta-level bias. These analyses showed mixed results. Indeed, Egger's test and Rank Correlation test did not, but the trim-and-fill method did suggest the occurrence of publication bias. Moreover, we did not find any association between publication year and reported effect size. Thus, the effect sizes did not decrease over time, and the data did not suggest any potential replication issues. Since the trim-and-fill method did not account for the multi-level structure of the data, the risk of publication bias appears relatively small. 

We also tested whether some methodological choices influenced the size of the effects. For instance, we found that measuring the CAT cardiovascular responses before or during performance was a significant moderator, but only for CTI. Unfortunately, there was a set of studies that included only CTI and did not provide data on CO and TPR separately (Dixon et al., 2020[34]; Miller et al., 2021[84][83]; Scheepers, 2017[104]; Slater et al., 2018[118]). Thus, we were unable to determine whether this methodological choice systematically influenced the strength of the association between CAT cardiovascular responses and performance outcomes or whether the additional studies including only CTI in their analyses drove this effect. Overall, the findings for CO and TPR are in line with a recent study, where researchers used multiverse analysis to examine the consistency of the effects when using different statistical and methodological approaches (Behnke et al., 2024[10]). Furthermore, similarly to the previous meta-analysis (Behnke and Kaczmarek, 2018[8]), age, gender, and publication year did not impact the size of the effects. Regarding publication year, the presence of the COVID-19 pandemic in the reviewed period makes the interpretation of publication year analyses more complicated when compared with previous meta-analyses (e.g., Behnke and Kaczmarek, 2018[8]). We had hoped to test if the results of studies conducted before and after COVID-19 differed but were unable to code exact data collection dates for 91 % of studies. Thus, we recommend adding detailed study metadata (e.g., date and year of collection) in future studies to better enable comparisons like this in future meta-analyses.

Our failed attempts to explain the heterogeneity of the mean effects with our moderator analyses suggest that there might be other factors influencing the strength of the association between CAT states and performance outcomes. To differentiate their impact on effect sizes and thereby elucidate the mechanisms of the CAT-performance relationship, future research could include attentional, emotional, and behavioral processes in the study of the CAT-performance relationship (Jones et al., 2009[64]; Vine et al., 2016[131]). Indeed, some of the studies included in this review and meta-analysis set out to test possible mechanisms underlying the CAT-performance relationship. For instance, Brimmell et al. (2019[20]) found that experienced soccer players who exhibited a challenge state displayed superior attentional control (e.g., longer quiet eye durations) to players who exhibited a threat state. Moreover, Wood and colleagues (2018[134]) found that participants in a challenge state reported more positive affect, lower perceived exertion, and less self-focused attention (e.g., on movements) during a pressurized Wingate test compared to participants in a threat state. As the exact processes behind the small but robust effects of CAT on performance outcomes remain unclear, far more research on how challenge states improve performance more than threat states is required to extend the findings of this review and meta-analysis. 

Our review has several practical implications, suggesting that people are less likely to succeed when they perceive their coping resources as insufficient to meet situational demands. The findings indicate that having strong confidence in one's coping resources before a motivated performance situation (e.g., exam, sporting competition) is linked to better outcomes, underscoring the importance of fostering a positive mindset about one's coping resources during training and pre-competition phases as part of comprehensive psychological preparation. Previous studies have shown that it is possible to promote challenge states through various strategies, such as encouraging task-focused instructions (Moore et al., 2012[90]), providing performance-related feedback (Behnke et al., 2020[9]), using self-talk (Hase et al., 2019[49]), reappraising arousal (Sammy et al., 2017[103]), applying imagery techniques (Williams et al., 2010[133]), or engaging in pressure training (Kent et al., 2022[66]). More recently, efforts have been made to develop interventions (e.g., arousal reappraisal; synergistic mindset) specifically designed to help individuals adopt more challenge-like mindsets (Behnke et al., 2024[10]; Sharpe et al., 2024[115]; Yeager et al., 2022[135]). We encourage researchers and practitioners to test existing interventions further and develop new ones (e.g., if-then planning; Bieleke et al., 2021[12]), and for a systematic review and meta-analysis to summarize and synthesize the research conducted to date on interventions designed to foster a challenge (vs. threat) state, highlighting which strategies are most effective.

### Strengths and limitations 

The key strengths of the present systematic review and meta-analysis include the update and quantitative synthesis of relevant BPSM-based CAT research results since 2017. Specifically, the application of a meta-analysis, as well as the inclusion of both CAT cognitive evaluations and cardiovascular responses, provide advances from the previous work by Behnke and Kaczmarek (2018[8]) and Hase et al. (2019[50]). 

The current meta-analysis is also limited in some ways. For example, some relevant research results could not be synthesized in the meta-analysis because the publications did not report the necessary statistics, or the authors did not respond to our inquiries. Although this is not unheard of in meta-analytical research (e.g., Behnke and Kaczmarek, 2018[8]; Behnke et al., 2022[7], 2023[11]), the absence of these findings naturally implies room for improvement in the present paper's results. The absence of this data introduces a potential risk of bias in our findings, as the studies for which data could not be obtained may differ systematically from those included in the analysis (Page et al., 2022[96]). To address this limitation in future reviews and meta-analyses, we recommend openly sharing the code and data from the analysis. This approach would enhance transparency and allow for more comprehensive and unbiased synthesis of the evidence. Next, we observed some improvement in the range of participants' age from previous meta-analyses (e.g., Gurera and Isaacowitz, 2022[43]; Mulvenna et al., 2023[94]; Turner et al., 2021[125]), still most studies have been conducted with samples of young adults, limiting the generalizability of our findings beyond this age group. To better understand the robustness of the BPSM across different stages of life, more research is needed, as cardiovascular efficiency varies with age (Mitchell et al., 2004[85]). As a result, there is little knowledge about whether the physiological benefits associated with a challenge state also translate into improved outcomes in older adults. 

Finally, the scope of the present review was restricted to a BPSM-based view of CAT states. Some research focusing on separate and independent CAT subjective measures may therefore not have been included (e.g., work underpinned by the theory of Lazarus and Folkman, 1984[70]). Thus, it remains for future research to synthesize the findings from studies that conceptualize CAT in distinct ways and for this work to consider outcomes that are not solely performance-related (e.g., health, well-being). Indeed, research suggests that threat states, when frequently experienced, may be linked with poorer health and well-being (e.g., Brown et al., 2021[21]; McLoughlin et al., 2024[79]; Yeager et al., 2022[135]). 

## Conclusion

Given the results of this systematic review and meta-analysis, we conclude that the published literature largely supports the predictions of the BPSM, with a challenge state associated with superior performance outcomes than a threat state. However, we also conclude that the field would benefit from more empirical research to test the robustness of the observed effects, especially for the separate components of CAT cardiovascular response, where the aggregate effects did not reach statistical significance (e.g., for CO reactivity), despite being in the expected direction and the single cardiovascular CAT index reaching significance. We also conclude that the methodological quality of the studies was generally high, and the risk of publication bias was relatively small. We encourage researchers to continue investigating the predictions of the BPSM using robust methods that effectively address these publication bias and replicability issues, for example, using pre-registered studies with pre-defined analysis plans and open-access data. Given that three reviews now support the performance benefits of a challenge state relative to a threat state, further research is needed to explore the possible underlying mechanisms driving these effects (e.g., attentional, behavioral, emotional, physiological). Future work also needs to identify optimal intervention strategies for fostering a challenge state and, in turn, improving performance.

## Declaration

### Declaration of Generative AI and AI-assisted technologies in the writing process

During the preparation of this work, the authors used Grammarly and ChatGPT4.0 to improve the readability and language of parts of the manuscript (e.g., Introduction and Discussion). After using these services, the authors reviewed and edited the content as needed and take full responsibility for the content.

### Acknowledgments

The National Science Centre in Poland supported preparing this article with a research grant (UMO-2020/39/B/HS6/00685) awarded to MB. The funder had no role in research design, analysis, publishing decisions, or manuscript preparation.

### Author contributions

AH: conceptualization, data curation, formal analysis, funding acquisition, investigation, methodology, project administration, supervision, visualization, writing - original draft, writing - review and editing.

MN: conceptualization, data curation, investigation, writing - original draft, writing - review and editing.

MK: data curation, investigation, writing - original draft, writing - review and editing. 

KS: data curation, investigation, writing - original draft, writing - review and editing. 

LM: supervision, writing - original draft, writing - review and editing. 

JPJ: supervision, writing - original draft, writing - review and editing. 

MB: conceptualization, data curation, formal analysis, funding acquisition, investingation, methodology, project administration, supervision, visualization, writing - original draft, writing - review and editing. 

All authors gave final approval for publication and agreed to be held accountable for the work performed therein.

### Data accessibility

All data and analysis code used for analysis and its output are available on the *Open Science Framework (OSF)* website: https://osf.io/e5kz8. 

## Supplementary Material

Supplementary data

Supplementary information

## Figures and Tables

**Table 1:**
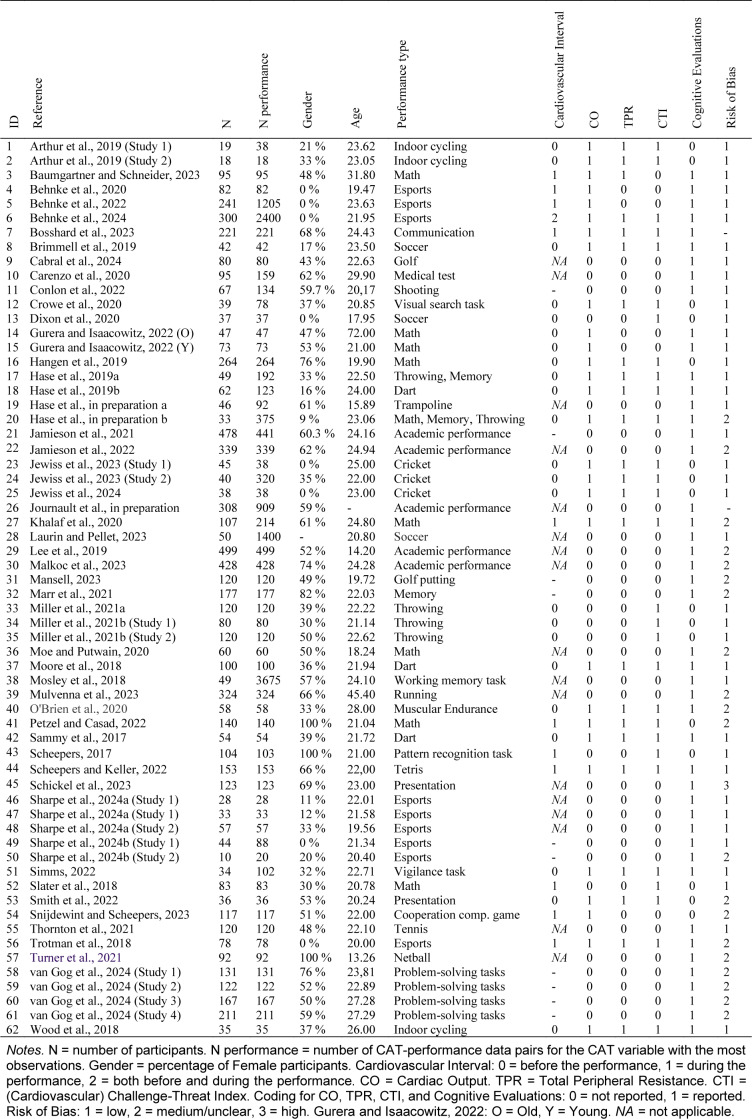
List of studies included in the meta-analysis

**Table 2:**
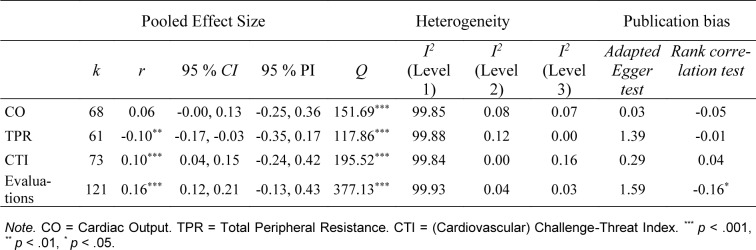
Summary of meta-analytic review of the association between challenge and threat cardiovascular responses and/or cognitive evaluations and performance outcomes

**Table 3:**
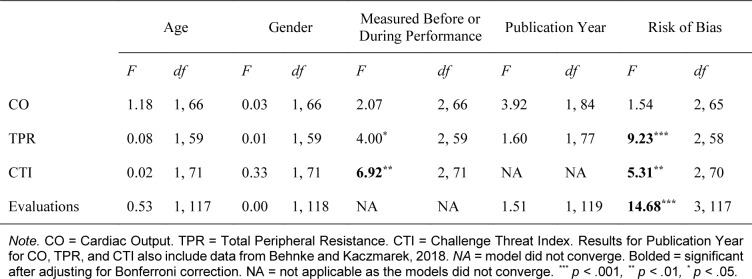
Results of the moderator analyses for associations between challenge and threat cardiovascular responses and cognitive evaluations and performance outcomes
