# Supplementary material for: The effects of challenge and threat states on performance outcomes: An updated review and meta-analysis of recent findings
Source: EXCLI J. 2025 Jan 16;24:151–76. doi: 10.17179/excli2024-7995 (PMC11869992; doi:10.17179/excli2024-7995)
Supplement: Supplementary information [file EXCLI-24-151-s-002.pdf]

1. Loading and installing all needed packages
2. Loading data
3. Risk of bias
4. Preparing the data
5. Cardiac Output
6. Total Peripheral Resistance
7. Cardiovascular Challenge and Threat Index
8. Cognitive Challenge and Threat Index
9. Results table
10. Figures
11 Moderator Analysis

The effects of challenge and threat states on performance: An updated review and meta-analysis of recent findings

Maciej Behnke & Adrian Hase

2024-03-28

This is code for and the results of analysis included in the manuscript:

Link to OSF:

1. Loading and installing all needed packages

```
# Clear the environment
rm(list = ls())

# Function to check, install (if necessary), and load the required packages
load_required_packages <- function(packages) {

  # Function to install and load a package
  install_and_load <- function(package) {
    if (!require(package, character.only = TRUE)) {
      install.packages(package, dependencies = TRUE)
      library(package, character.only = TRUE)
    }
  }

  # Iterate through the list of packages and install/load them
  sapply(packages, install_and_load)
}

# List of required packages
packages <- c("metafor"
,"robumeta"
,"ggplot2"
,"readxl"
,"clubSandwich",
"esc",
"robvis")



# Call the function to install and load the required packages
load_required_packages(packages)
```

```
## Ładowanie wymaganego pakietu: metafor
```

```
## Warning: pakiet 'metafor' został zbudowany w wersji R 4.3.3
```

```
## Ładowanie wymaganego pakietu: Matrix
```

```
## Warning: pakiet 'Matrix' został zbudowany w wersji R 4.3.3
```

```
## Ładowanie wymaganego pakietu: metadat
```

```
## Ładowanie wymaganego pakietu: numDeriv
```

```
##
## Loading the 'metafor' package (version 4.6-0). For an
## introduction to the package please type: help(metafor)
```

```
## Ładowanie wymaganego pakietu: robumeta
```

```
## Warning: pakiet 'robumeta' został zbudowany w wersji R 4.3.3
```

```
## Ładowanie wymaganego pakietu: ggplot2
```

```
## Warning: pakiet 'ggplot2' został zbudowany w wersji R 4.3.3
```

```
## Ładowanie wymaganego pakietu: readxl
```

```
## Warning: pakiet 'readxl' został zbudowany w wersji R 4.3.3
```

```
## Ładowanie wymaganego pakietu: clubSandwich
```

```
## Warning: pakiet 'clubSandwich' został zbudowany w wersji R 4.3.3
```

```
## Registered S3 method overwritten by 'clubSandwich':
##   method    from
##   bread.mlm sandwich
```

```
## Ładowanie wymaganego pakietu: esc
```

```
## Ładowanie wymaganego pakietu: robvis
```

```
## Warning: pakiet 'robvis' został zbudowany w wersji R 4.3.3
```

```
## $metafor
## NULL
##
## $robumeta
## NULL
##
## $ggplot2
## NULL
##
## $readxl
## NULL
##
## $clubSandwich
## NULL
##
## $esc
## NULL
##
## $robvis
## NULL
```

2. Loading data

```
### set working directory
setwd("C:/Users/macbe/OneDrive/Behnke Dropbox/MA CHT")

#import and store data from excel file
Data <- read_excel("C:/Users/macbe/OneDrive/Behnke Dropbox/MA CHT/Data.xlsx", sheet =
"coding")
```

```
## New names:
## • `title` -> `title...5`
## • `title` -> `title...8`
## • `` -> `...31`
```

```
#View(Data)

Data <- as.data.frame(Data)

#drop non numeric data
#Data <- Data2 %>% select(-c(Date_Lab1,
 #
  #                                  ))
```

3. Risk of bias

```
## New names:
## • `` -> `...1`
```

```
## # A tibble: 5 × 7
##   Study `Randomisation process` Deviations from intende…¹ `Missing outcome data`
##   <chr> <chr>                   <chr>                     <chr>
## 1 2     Low                     Low                       Low
## 2 4     Low                     Low                       Low
## 3 14    Low                     Low                       Low
## 4 15    Low                     Low                       Low
## 5 55    Low                     Low                       Low
## # ℹ abbreviated name: ¹`Deviations from intended interventions`
## # ℹ 3 more variables: `Measurement of the outcome` <chr>,
## #   `Selection of the reported result` <chr>, Overall <chr>
```

```
## Warning in ggplot2::geom_point(shape = 1, colour = "black", size = psize, : All aest
hetics have length 1, but the data has 30 rows.
## ℹ Please consider using `annotate()` or provide this layer with data containing
##   a single row.
```

```
## png
##   2
```

```
## New names:
## • `` -> `...1`
```

```
## # A tibble: 6 × 8
##   Study Selection of participant…¹ Confounding variable…² Measurement of expos…³
##   <chr> <chr>                      <chr>                  <chr>
## 1 1     Low                        Low                    Low
## 2 3     Low                        Low                    Low
## 3 5     Low                        Low                    Low
## 4 6     Low                        Low                    Low
## 5 7     <NA>                       <NA>                   <NA>
## 6 8     Low                        Low                    Low
## # ℹ abbreviated names: ¹`Selection of participants`, ²`Confounding variables`,
## #   ³`Measurement of exposure`
## # ℹ 4 more variables: `Blinding of outcome assessments` <chr>,
## #   `Incomplete outcome data` <chr>, `Selective outcome reporting` <chr>,
## #   Overall <chr>
```

```
## Warning in ggplot2::geom_point(shape = 1, colour = "black", size = psize, : All aest
hetics have length 1, but the data has 399 rows.
## ℹ Please consider using `annotate()` or provide this layer with data containing
##   a single row.
```

```
## Warning: Removed 14 rows containing missing values or values outside the scale range
## (`geom_point()`).
## Removed 14 rows containing missing values or values outside the scale range
## (`geom_point()`).
```

```
## png
##   2
```

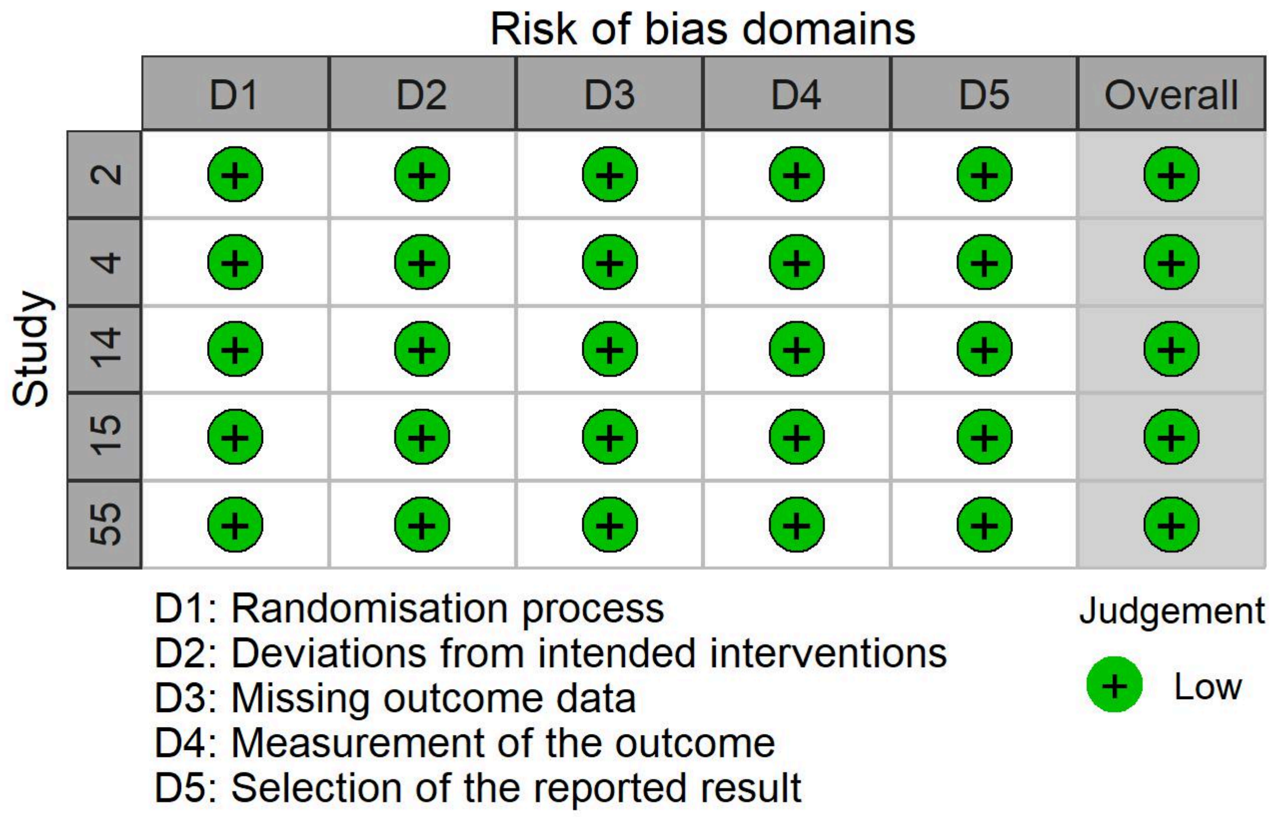
Risk of bias domains
D1
D2
D3
D4
D5
Overall
Study
2
4
14
15
55
D1: Randomisation process
D2: Deviations from intended interventions
D3: Missing outcome data
D4: Measurement of the outcome
D5: Selection of the reported result
Judgement
+
Low

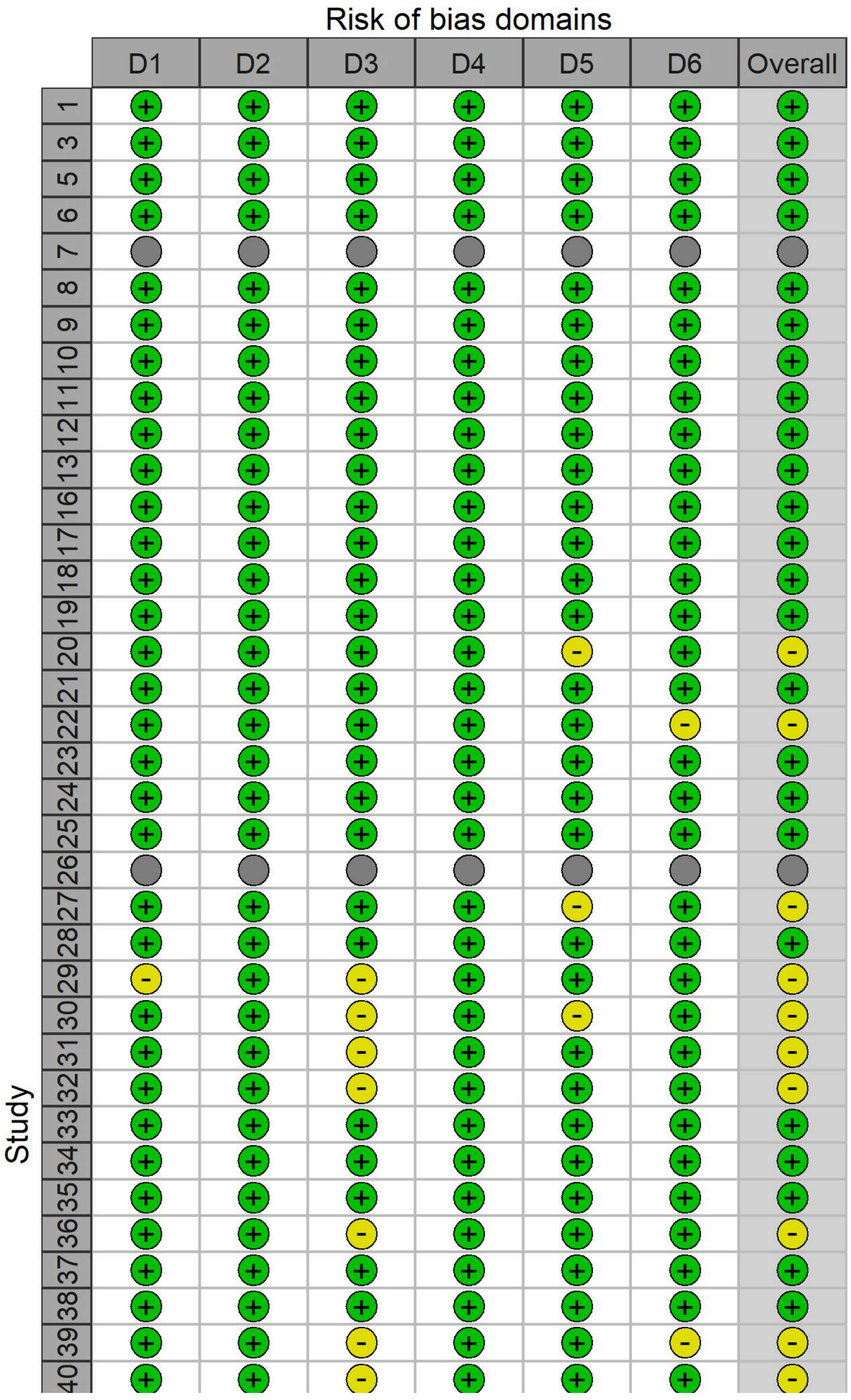
Risk of bias domains
D1
D2
D3
D4
D5
D6
Overall
Study
1
3
5
6
7
8
9
10
11
12
13
16
17
18
19
20
21
22
23
24
25
26
27
28
29
30
31
32
33
34
35
36
37
38
39
40

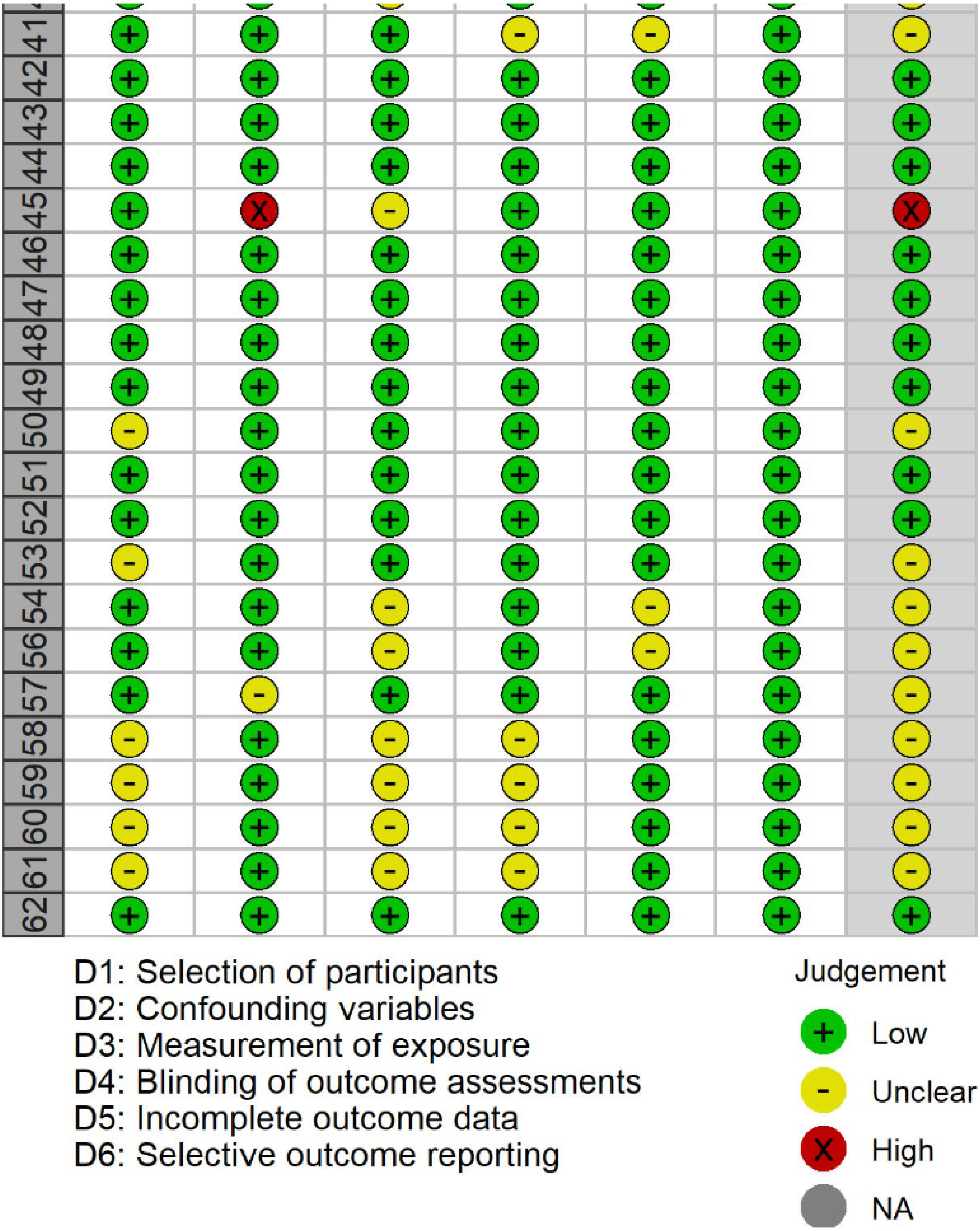
41
42
43
44
45
46
47
48
49
50
51
52
53
54
56
57
58
59
60
61
62
D1: Selection of participants
D2: Confounding variables
D3: Measurement of exposure
D4: Blinding of outcome assessments
D5: Incomplete outcome data
D6: Selective outcome reporting
Judgement
Low
Unclear
High
NA

4. Preparing the data

```
#z-transform correlation coefficients
Data$CO_cor_Z <- .5 * log((1+Data$CO_cor)/(1-Data$CO_cor))
Data$TPR_cor_Z <- .5 * log((1+Data$TPR_cor)/(1-Data$TPR_cor))
Data$CTI_cor_Z <- .5 * log((1+Data$CTI_cor)/(1-Data$CTI_cor))
Data$Cogni_cor_Z <- .5 * log((1+Data$Cogni_cor)/(1-Data$Cogni_cor))


# calculate z variance
Data$CO_cor_Z_var <- ifelse(is.na(Data$CO_cor), NA, 1 / (Data$n_performance - 3))
Data$TPR_cor_Z_var <- ifelse(is.na(Data$TPR_cor), NA, 1 / (Data$n_performance - 3))
Data$CTI_cor_Z_var <- ifelse(is.na(Data$CTI_cor), NA, 1 / (Data$n_performance - 3))
Data$Cogni_cor_Z_var <- ifelse(is.na(Data$Cogni_cor), NA, 1 / (Data$n_performance - 3))

# calculate z variance squared
Data$CO_cor_Z_var_Sq <- ifelse(is.na(Data$CO_cor), NA, (1 / (Data$n_performance - 3))^
2)
Data$TPR_cor_Z_var_Sq <- ifelse(is.na(Data$TPR_cor), NA, (1 / (Data$n_performance - 3))
^2)
Data$CTI_cor_Z_var_Sq <- ifelse(is.na(Data$CTI_cor), NA, (1 / (Data$n_performance - 3))
^2)
Data$Cogni_cor_Z_var_Sq <- ifelse(is.na(Data$Cogni_cor), NA, (1 / (Data$n_performance -
3))^2)
```

5. Cardiac Output

5.1 Identyfing outliers

```
# model for outlier analyses and funnel plots
model_fun_CO <- rma(yi = CO_cor_Z, vi= CO_cor_Z_var, data = Data, slab = Ref_APA)
```

```
## Warning: 94 studies with NAs omitted from model fitting.
```

```
model_fun_CO
```

```
##
## Random-Effects Model (k = 68; tau^2 estimator: REML)
##
## tau^2 (estimated amount of total heterogeneity): 0.0216 (SE = 0.0070)
## tau (square root of estimated tau^2 value):      0.1470
## I^2 (total heterogeneity / total variability):   72.40%
## H^2 (total variability / sampling variability):  3.62
##
## Test for Heterogeneity:
## Q(df = 67) = 151.6929, p-val < .0001
##
## Model Results:
##
## estimate      se    zval    pval    ci.lb   ci.ub
##   0.0473  0.0251  1.8842  0.0595  -0.0019  0.0964  .
##
## ---
## Signif. codes:  0 '***' 0.001 '**' 0.01 '*' 0.05 '.' 0.1 ' ' 1
```

```
# outliers diagnostics  - outlier rstudent > 3.0 #
Out_model_fun_CO <- influence(model_fun_CO)
Out_model_fun_CO
```

```
##
##                                 rstudent  dffits cook.d  cov.r tau2.del
## Arthur et al., 2019.1            1.0347  0.0898 0.0081 1.0076   0.0216
## Arthur et al., 2019.2            2.2513  0.1821 0.0327 0.9729   0.0203
## Arthur et al., 2019.3            0.1791  0.0165 0.0003 1.0164   0.0219
## Baumgartner & Schneider, 2023   -0.9394 -0.1317 0.0174 1.0218   0.0217
## Behnke et al., 2020.1           -0.1463 -0.0169 0.0003 1.0421   0.0225
## Behnke et al., 2020.2            0.5616  0.0789 0.0063 1.0355   0.0222
## Behnke et al., 2022              0.1511  0.0295 0.0009 1.0638   0.0229
## Behnke et al., 2024.1           -0.4481 -0.0716 0.0054 1.0577   0.0227
## Behnke et al., 2024.2            0.0193  0.0079 0.0001 1.0656   0.0230
## Brimmell et al., 2019            2.9532  0.3154 0.0902 0.8960   0.0172
## Crowe et al., 2020.1            -0.1674 -0.0169 0.0003 1.0291   0.0222
## Crowe et al., 2020.2            -0.8044 -0.0907 0.0083 1.0183   0.0218
## Crowe et al., 2020.3             0.2848  0.0345 0.0012 1.0285   0.0222
## Crowe et al., 2020.4            -0.4836 -0.0534 0.0029 1.0255   0.0221
## Crowe et al., 2020.5             0.5611  0.0655 0.0043 1.0248   0.0220
## Gurera & Isaacowitz, 2022.1      0.9151  0.1228 0.0151 1.0222   0.0218
## Gurera & Isaacowitz, 2022.3     -0.5247 -0.0613 0.0038 1.0276   0.0221
## Hangen et al., 2019.1           -0.0442 -0.0028 0.0000 1.0571   0.0228
## Hangen et al., 2019.2            0.1425  0.0264 0.0007 1.0567   0.0228
## Hase et al., 2019.1             -1.3587 -0.1669 0.0275 0.9981   0.0210
## Hase et al., 2019.2             -1.1428 -0.1379 0.0189 1.0081   0.0214
## Hase et al., 2019.3             -0.3883 -0.0451 0.0021 1.0307   0.0222
## Hase et al., 2019.4             -0.1056 -0.0102 0.0001 1.0328   0.0223
## Hase et al., 2019.5             -0.7552 -0.0961 0.0093 1.0252   0.0219
## Hase et al., 2019.6             -1.1732 -0.1523 0.0230 1.0078   0.0213
## Hase et al., in preparation.3   -1.1650 -0.1260 0.0158 1.0057   0.0214
## Hase et al., in preparation.4   -1.4301 -0.1537 0.0234 0.9957   0.0210
## Hase et al., in preparation.5   -0.2418 -0.0240 0.0006 1.0251   0.0221
## Hase et al., in preparation.6   -0.7622 -0.0814 0.0066 1.0175   0.0218
## Hase et al., in preparation.7    0.0968  0.0123 0.0002 1.0261   0.0222
## Hase et al., in preparation.8   -0.1995 -0.0194 0.0004 1.0255   0.0221
## Hase et al., in preparation.9    1.5401  0.1587 0.0249 0.9932   0.0209
## Hase et al., in preparation.10  -1.0385 -0.1083 0.0117 1.0093   0.0215
## Hase et al., in preparation.11  -0.0297 -0.0012 0.0000 1.0250   0.0221
## Hase et al., in preparation.12   0.0117  0.0031 0.0000 1.0250   0.0221
## Hase et al., in preparation.13  -0.4023 -0.0405 0.0017 1.0225   0.0220
## Hase et al., in preparation.14  -0.1952 -0.0186 0.0003 1.0244   0.0221
## Jewiss et al., 2023 (Study 1)    1.4804  0.1636 0.0264 0.9948   0.0209
## Jewiss et al., 2023 (Study 2).1  1.3558  0.1530 0.0232 1.0003   0.0211
## Jewiss et al., 2023 (Study 2).2 -0.4416 -0.0489 0.0024 1.0265   0.0221
## Jewiss et al., 2023 (Study 2).3 -0.1687 -0.0172 0.0003 1.0296   0.0222
## Jewiss et al., 2023 (Study 2).4  0.8033  0.0930 0.0087 1.0197   0.0218
## Jewiss et al., 2023 (Study 2).5  0.4719  0.0560 0.0032 1.0267   0.0221
## Jewiss et al., 2023 (Study 2).6  0.1952  0.0246 0.0006 1.0297   0.0222
## Jewiss et al., 2023 (Study 2).7  0.6598  0.0770 0.0060 1.0232   0.0220
## Jewiss et al., 2023 (Study 2).8  0.3791  0.0455 0.0021 1.0280   0.0222
## Jewiss et al., 2024             -0.3899 -0.0422 0.0018 1.0265   0.0221
## Khalaf et al., 2020.1            0.2996  0.0459 0.0022 1.0447   0.0225
## Khalaf et al., 2020.2            0.3568  0.0539 0.0030 1.0438   0.0225
## Moore et al., 2017               1.0957  0.1549 0.0239 1.0157   0.0214
## O'Brien et al., 2022            -0.8356 -0.1052 0.0111 1.0217   0.0218
## Petzel & Casad, 2022.1           5.0892  0.5536 0.1822 0.5913   0.0068
## Petzel & Casad, 2022.2          -0.7755 -0.1015 0.0104 1.0258   0.0219
```

```
## Sammy et al., 2017                -0.9385 -0.1166 0.0136 1.0172   0.0217
## Smith et al., 2022.1              -1.4556 -0.1630 0.0262 0.9942   0.0209
## Smith et al., 2022.2              -1.6612 -0.1866 0.0341 0.9845   0.0205
## Smith et al., 2022.3              -2.4387 -0.2758 0.0720 0.9385   0.0188
## Snijdewint & Scheepers, 2023      -0.2693 -0.0357 0.0013 1.0459   0.0225
## Trotman et al., 2018.2             1.4593  0.1946 0.0368 0.9934   0.0207
## Wood et al., 2018                 -0.4982 -0.0533 0.0029 1.0235   0.0220
## Scheepers & Keller, 2022           0.3503  0.0557 0.0032 1.0483   0.0226
## Bosshard et al., 2023.2            0.3202  0.0523 0.0028 1.0508   0.0226
## Simms, 2022.1                      0.5999  0.0668 0.0045 1.0221   0.0220
## Simms, 2022.2                     -1.1956 -0.1307 0.0170 1.0048   0.0213
## Simms, 2022.3                     -0.3677 -0.0384 0.0015 1.0249   0.0221
## Simms, 2022.4                      1.1624  0.1256 0.0157 1.0074   0.0214
## Simms, 2022.5                     -0.4457 -0.0470 0.0022 1.0239   0.0221
## Simms, 2022.6                      0.0382  0.0062 0.0000 1.0273   0.0222
##                                    QE.del    hat weight    dfbs inf
## Arthur et al., 2019.1            150.1654 0.0075 0.7478  0.0898
## Arthur et al., 2019.2            144.9198 0.0071 0.7125  0.1839
## Arthur et al., 2019.3            151.6335 0.0071 0.7125  0.0165
## Baumgartner & Schneider, 2023    149.3719 0.0194 1.9370 -0.1317
## Behnke et al., 2020.1            151.6685 0.0184 1.8358 -0.0169
## Behnke et al., 2020.2            150.6638 0.0184 1.8358  0.0790
## Behnke et al., 2022              150.2281 0.0280 2.8039  0.0299
## Behnke et al., 2024.1            141.1102 0.0286 2.8567 -0.0723
## Behnke et al., 2024.2            151.1877 0.0286 2.8567  0.0080
## Brimmell et al., 2019            136.7619 0.0133 1.3313  0.3193
## Crowe et al., 2020.1             151.6656 0.0127 1.2737 -0.0169
## Crowe et al., 2020.2             150.6703 0.0127 1.2737 -0.0907
## Crowe et al., 2020.3             151.5005 0.0127 1.2737  0.0345
## Crowe et al., 2020.4             151.3490 0.0127 1.2737 -0.0533
## Crowe et al., 2020.5             151.0422 0.0127 1.2737  0.0654
## Gurera & Isaacowitz, 2022.1      149.3577 0.0175 1.7526  0.1228
## Gurera & Isaacowitz, 2022.3      151.2458 0.0142 1.4188 -0.0612
## Hangen et al., 2019.1            151.6911 0.0247 2.4731 -0.0028
## Hangen et al., 2019.2            151.4071 0.0247 2.4731  0.0266
## Hase et al., 2019.1              148.3503 0.0145 1.4511 -0.1671
## Hase et al., 2019.2              149.3862 0.0142 1.4188 -0.1379
## Hase et al., 2019.3              151.4593 0.0145 1.4511 -0.0450
## Hase et al., 2019.4              151.6859 0.0142 1.4188 -0.0102
## Hase et al., 2019.5              150.5634 0.0163 1.6315 -0.0961
## Hase et al., 2019.6              148.8804 0.0163 1.6315 -0.1523
## Hase et al., in preparation.3    149.6406 0.0114 1.1448 -0.1261
## Hase et al., in preparation.4    148.6173 0.0112 1.1214 -0.1541
## Hase et al., in preparation.5    151.6273 0.0112 1.1214 -0.0240
## Hase et al., in preparation.6    150.8412 0.0114 1.1448 -0.0813
## Hase et al., in preparation.7    151.6619 0.0112 1.1214  0.0123
## Hase et al., in preparation.8    151.6520 0.0112 1.1214 -0.0194
## Hase et al., in preparation.9    147.7581 0.0110 1.0973  0.1592
## Hase et al., in preparation.10   150.1305 0.0107 1.0725 -0.1083
## Hase et al., in preparation.11   151.6927 0.0107 1.0725 -0.0012
## Hase et al., in preparation.12   151.6886 0.0107 1.0725  0.0031
## Hase et al., in preparation.13   151.4854 0.0107 1.0725 -0.0404
## Hase et al., in preparation.14   151.6548 0.0107 1.0725 -0.0186
## Jewiss et al., 2023 (Study 1)    147.7007 0.0125 1.2535  0.1639
## Jewiss et al., 2023 (Study 2).1 148.2297 0.0129 1.2933  0.1532
## Jewiss et al., 2023 (Study 2).2 151.4083 0.0129 1.2933 -0.0488
```

```
## Jewiss et al., 2023 (Study 2).3 151.6648 0.0129 1.2933 -0.0171
## Jewiss et al., 2023 (Study 2).4 150.4054 0.0129 1.2933  0.0929
## Jewiss et al., 2023 (Study 2).5 151.2130 0.0129 1.2933  0.0559
## Jewiss et al., 2023 (Study 2).6 151.5895 0.0129 1.2933  0.0246
## Jewiss et al., 2023 (Study 2).7 150.8021 0.0129 1.2933  0.0770
## Jewiss et al., 2023 (Study 2).8 151.3698 0.0129 1.2933  0.0454
## Jewiss et al., 2024             151.4819 0.0125 1.2535 -0.0422
## Khalaf et al., 2020.1           151.2775 0.0201 2.0148  0.0460
## Khalaf et al., 2020.2           151.1331 0.0201 2.0148  0.0541
## Moore et al., 2017              147.6524 0.0197 1.9710  0.1548
## O'Brien et al., 2022            150.3460 0.0158 1.5809 -0.1052
## Petzel & Casad, 2022.1          113.1970 0.0172 1.7219  0.5598   *
## Petzel & Casad, 2022.2          150.4113 0.0172 1.7219 -0.1015
## Sammy et al., 2017              150.0421 0.0153 1.5262 -0.1166
## Smith et al., 2022.1            148.3529 0.0121 1.2117 -0.1634
## Smith et al., 2022.2            147.3424 0.0121 1.2117 -0.1873
## Smith et al., 2022.3            142.4886 0.0121 1.2117 -0.2787
## Snijdewint & Scheepers, 2023    151.5308 0.0207 2.0708 -0.0358
## Trotman et al., 2018.2          145.9160 0.0180 1.8004  0.1943
## Wood et al., 2018               151.3417 0.0119 1.1900 -0.0532
## Scheepers & Keller, 2022        150.9690 0.0223 2.2250  0.0560
## Bosshard et al., 2023.2         150.9722 0.0231 2.3134  0.0526
## Simms, 2022.1                   151.0057 0.0117 1.1677  0.0667
## Simms, 2022.2                   149.5031 0.0117 1.1677 -0.1308
## Simms, 2022.3                   151.5156 0.0117 1.1677 -0.0383
## Simms, 2022.4                   149.2972 0.0117 1.1677  0.1257
## Simms, 2022.5                   151.4207 0.0117 1.1677 -0.0469
## Simms, 2022.6                   151.6821 0.0117 1.1677  0.0062
```

```
rank_corr_test_CO <- ranktest(model_fun_CO)
```

```
## Warning in cor.test.default(yi.star, vi, method = "kendall", exact = exact):
## nie można obliczyć dokładnej wartości prawdopodobieństwa z powtórzonymi
## wartościami
```

```
rank_corr_test_CO
```

```
##
## Rank Correlation Test for Funnel Plot Asymmetry
##
## Kendall's tau = -0.0541, p = 0.5210
```

```
# leave one out analysis #

leave1out(model_fun_CO)
```

```
## 
##                                  estimate     se   zval   pval   ci.lb  ci.ub
## Arthur et al., 2019.1              0.0450 0.0252 1.7877 0.0738 -0.0043 0.0943
## Arthur et al., 2019.2              0.0427 0.0247 1.7270 0.0842 -0.0058 0.0912
## Arthur et al., 2019.3              0.0468 0.0253 1.8526 0.0639 -0.0027 0.0964
## Baumgartner & Schneider, 2023      0.0506 0.0253 1.9945 0.0461  0.0009 0.1002
## Behnke et al., 2020.1              0.0477 0.0256 1.8625 0.0625 -0.0025 0.0979
## Behnke et al., 2020.2              0.0453 0.0255 1.7734 0.0762 -0.0048 0.0953
## Behnke et al., 2022                0.0465 0.0259 1.7974 0.0723 -0.0042 0.0972
## Behnke et al., 2024.1              0.0491 0.0258 1.9034 0.0570 -0.0015 0.0996
## Behnke et al., 2024.2              0.0470 0.0259 1.8174 0.0692 -0.0037 0.0978
## Brimmell et al., 2019              0.0397 0.0237 1.6733 0.0943 -0.0068 0.0862
## Crowe et al., 2020.1               0.0477 0.0254 1.8741 0.0609 -0.0022 0.0975
## Crowe et al., 2020.2               0.0495 0.0253 1.9573 0.0503 -0.0001 0.0991
## Crowe et al., 2020.3               0.0464 0.0254 1.8236 0.0682 -0.0035 0.0962
## Crowe et al., 2020.4               0.0486 0.0254 1.9137 0.0557 -0.0012 0.0984
## Crowe et al., 2020.5               0.0456 0.0254 1.7963 0.0724 -0.0042 0.0954
## Gurera & Isaacowitz, 2022.1        0.0442 0.0254 1.7419 0.0815 -0.0055 0.0939
## Gurera & Isaacowitz, 2022.3        0.0488 0.0254 1.9196 0.0549 -0.0010 0.0986
## Hangen et al., 2019.1              0.0473 0.0258 1.8354 0.0664 -0.0032 0.0979
## Hangen et al., 2019.2              0.0466 0.0258 1.8067 0.0708 -0.0039 0.0971
## Hase et al., 2019.1                0.0514 0.0251 2.0519 0.0402  0.0023 0.1005
## Hase et al., 2019.2                0.0507 0.0252 2.0136 0.0441  0.0014 0.1000
## Hase et al., 2019.3                0.0484 0.0255 1.9007 0.0573 -0.0015 0.0983
## Hase et al., 2019.4                0.0475 0.0255 1.8642 0.0623 -0.0024 0.0975
## Hase et al., 2019.5                0.0497 0.0254 1.9563 0.0504 -0.0001 0.0994
## Hase et al., 2019.6                0.0511 0.0252 2.0280 0.0426  0.0017 0.1004
## Hase et al., in preparation.3      0.0504 0.0251 2.0042 0.0450  0.0011 0.0997
## Hase et al., in preparation.4      0.0511 0.0250 2.0416 0.0412  0.0020 0.1001
## Hase et al., in preparation.5      0.0479 0.0254 1.8848 0.0595 -0.0019 0.0976
## Hase et al., in preparation.6      0.0493 0.0253 1.9488 0.0513 -0.0003 0.0989
## Hase et al., in preparation.7      0.0469 0.0254 1.8479 0.0646 -0.0028 0.0967
## Hase et al., in preparation.8      0.0477 0.0254 1.8800 0.0601 -0.0020 0.0975
## Hase et al., in preparation.9      0.0433 0.0250 1.7324 0.0832 -0.0057 0.0923
## Hase et al., in preparation.10     0.0500 0.0252 1.9833 0.0473  0.0006 0.0993
## Hase et al., in preparation.11     0.0473 0.0254 1.8623 0.0626 -0.0025 0.0970
## Hase et al., in preparation.12     0.0472 0.0254 1.8580 0.0632 -0.0026 0.0969
## Hase et al., in preparation.13     0.0483 0.0254 1.9036 0.0570 -0.0014 0.0980
## Hase et al., in preparation.14     0.0477 0.0254 1.8802 0.0601 -0.0020 0.0975
## Jewiss et al., 2023 (Study 1)      0.0432 0.0250 1.7262 0.0843 -0.0058 0.0922
## Jewiss et al., 2023 (Study 2).1    0.0434 0.0251 1.7318 0.0833 -0.0057 0.0926
## Jewiss et al., 2023 (Study 2).2    0.0485 0.0254 1.9082 0.0564 -0.0013 0.0983
## Jewiss et al., 2023 (Study 2).3    0.0477 0.0254 1.8740 0.0609 -0.0022 0.0976
## Jewiss et al., 2023 (Study 2).4    0.0449 0.0253 1.7736 0.0761 -0.0047 0.0945
## Jewiss et al., 2023 (Study 2).5    0.0458 0.0254 1.8041 0.0712 -0.0040 0.0956
## Jewiss et al., 2023 (Study 2).6    0.0466 0.0254 1.8325 0.0669 -0.0032 0.0965
## Jewiss et al., 2023 (Study 2).7    0.0453 0.0254 1.7863 0.0740 -0.0044 0.0950
## Jewiss et al., 2023 (Study 2).8    0.0461 0.0254 1.8133 0.0698 -0.0037 0.0959
## Jewiss et al., 2024                0.0483 0.0254 1.9017 0.0572 -0.0015 0.0981
## Khalaf et al., 2020.1              0.0461 0.0256 1.7979 0.0722 -0.0042 0.0963
## Khalaf et al., 2020.2              0.0459 0.0256 1.7908 0.0733 -0.0043 0.0961
## Moore et al., 2017                 0.0434 0.0253 1.7163 0.0861 -0.0062 0.0929
## O'Brien et al., 2022               0.0499 0.0253 1.9685 0.0490  0.0002 0.0996
## Petzel & Casad, 2022.1             0.0365 0.0193 1.8954 0.0580 -0.0012 0.0743
## Petzel & Casad, 2022.2             0.0498 0.0254 1.9610 0.0499  0.0000 0.0996
```

```
## Sammy et al., 2017               0.0502 0.0253 1.9839 0.0473  0.0006 0.0997
## Smith et al., 2022.1             0.0513 0.0250 2.0521 0.0402  0.0023 0.1003
## Smith et al., 2022.2             0.0519 0.0249 2.0852 0.0371  0.0031 0.1006
## Smith et al., 2022.3             0.0540 0.0243 2.2220 0.0263  0.0064 0.1016
## Snijdewint & Scheepers, 2023     0.0482 0.0256 1.8778 0.0604 -0.0021 0.0984
## Trotman et al., 2018.2           0.0424 0.0250 1.6980 0.0895 -0.0065 0.0914
## Wood et al., 2018                0.0486 0.0254 1.9153 0.0554 -0.0011 0.0983
## Scheepers & Keller, 2022         0.0458 0.0257 1.7850 0.0743 -0.0045 0.0962
## Bosshard et al., 2023.2          0.0459 0.0257 1.7862 0.0741 -0.0045 0.0963
## Simms, 2022.1                    0.0456 0.0254 1.7975 0.0723 -0.0041 0.0953
## Simms, 2022.2                    0.0505 0.0251 2.0098 0.0445  0.0013 0.0998
## Simms, 2022.3                    0.0482 0.0254 1.8993 0.0575 -0.0015 0.0980
## Simms, 2022.4                    0.0441 0.0252 1.7524 0.0797 -0.0052 0.0934
## Simms, 2022.5                    0.0484 0.0254 1.9088 0.0563 -0.0013 0.0982
## Simms, 2022.6                    0.0471 0.0254 1.8529 0.0639 -0.0027 0.0969
##                                         Q     Qp   tau2      I2     H2
## Arthur et al., 2019.1          150.1654 0.0000 0.0216 72.6522 3.6566
## Arthur et al., 2019.2          144.9198 0.0000 0.0203 71.4010 3.4966
## Arthur et al., 2019.3          151.6335 0.0000 0.0219 72.9742 3.7002
## Baumgartner & Schneider, 2023   149.3719 0.0000 0.0217 72.5101 3.6377
## Behnke et al., 2020.1          151.6685 0.0000 0.0225 73.2766 3.7420
## Behnke et al., 2020.2          150.6638 0.0000 0.0222 73.0548 3.7112
## Behnke et al., 2022            150.2281 0.0000 0.0229 70.5962 3.4009
## Behnke et al., 2024.1          141.1102 0.0000 0.0227 67.6273 3.0890
## Behnke et al., 2024.2          151.1877 0.0000 0.0230 67.9185 3.1171
## Brimmell et al., 2019          136.7619 0.0000 0.0172 67.8293 3.1084
## Crowe et al., 2020.1           151.6656 0.0000 0.0222 73.1543 3.7250
## Crowe et al., 2020.2           150.6703 0.0000 0.0218 72.7846 3.6744
## Crowe et al., 2020.3           151.5005 0.0000 0.0222 73.1341 3.7222
## Crowe et al., 2020.4           151.3490 0.0000 0.0221 73.0294 3.7077
## Crowe et al., 2020.5           151.0422 0.0000 0.0220 73.0065 3.7046
## Gurera & Isaacowitz, 2022.1    149.3577 0.0000 0.0218 72.6528 3.6567
## Gurera & Isaacowitz, 2022.3    151.2458 0.0000 0.0221 73.0279 3.7075
## Hangen et al., 2019.1          151.6911 0.0000 0.0228 73.0550 3.7113
## Hangen et al., 2019.2          151.4071 0.0000 0.0228 73.0407 3.7093
## Hase et al., 2019.1            148.3503 0.0000 0.0210 71.9739 3.5681
## Hase et al., 2019.2            149.3862 0.0000 0.0214 72.3501 3.6166
## Hase et al., 2019.3            151.4593 0.0000 0.0222 73.1168 3.7198
## Hase et al., 2019.4            151.6859 0.0000 0.0223 73.2059 3.7322
## Hase et al., 2019.5            150.5634 0.0000 0.0219 72.8294 3.6804
## Hase et al., 2019.6            148.8804 0.0000 0.0213 72.2188 3.5996
## Hase et al., in preparation.3   149.6406 0.0000 0.0214 72.4063 3.6240
## Hase et al., in preparation.4   148.6173 0.0000 0.0210 72.0565 3.5787
## Hase et al., in preparation.5   151.6273 0.0000 0.0221 73.0915 3.7163
## Hase et al., in preparation.6   150.8412 0.0000 0.0218 72.8177 3.6789
## Hase et al., in preparation.7   151.6619 0.0000 0.0222 73.1234 3.7207
## Hase et al., in preparation.8   151.6520 0.0000 0.0221 73.1019 3.7177
## Hase et al., in preparation.9   147.7581 0.0000 0.0209 71.9754 3.5683
## Hase et al., in preparation.10  150.1305 0.0000 0.0215 72.5660 3.6451
## Hase et al., in preparation.11  151.6927 0.0000 0.0221 73.1089 3.7187
## Hase et al., in preparation.12  151.6886 0.0000 0.0221 73.1101 3.7189
## Hase et al., in preparation.13  151.4854 0.0000 0.0220 73.0235 3.7069
## Hase et al., in preparation.14  151.6548 0.0000 0.0221 73.0875 3.7158
## Jewiss et al., 2023 (Study 1)   147.7007 0.0000 0.0209 71.9587 3.5662
## Jewiss et al., 2023 (Study 2).1 148.2297 0.0000 0.0211 72.1366 3.5889
## Jewiss et al., 2023 (Study 2).2 151.4083 0.0000 0.0221 73.0567 3.7115
```

```
## Jewiss et al., 2023 (Study 2).3 151.6648 0.0000 0.0222 73.1596 3.7257
## Jewiss et al., 2023 (Study 2).4 150.4054 0.0000 0.0218 72.8229 3.6796
## Jewiss et al., 2023 (Study 2).5 151.2130 0.0000 0.0221 73.0613 3.7121
## Jewiss et al., 2023 (Study 2).6 151.5895 0.0000 0.0222 73.1622 3.7261
## Jewiss et al., 2023 (Study 2).7 150.8021 0.0000 0.0220 72.9419 3.6957
## Jewiss et al., 2023 (Study 2).8 151.3698 0.0000 0.0222 73.1051 3.7182
## Jewiss et al., 2024             151.4819 0.0000 0.0221 73.0737 3.7138
## Khalaf et al., 2020.1           151.2775 0.0000 0.0225 73.2321 3.7358
## Khalaf et al., 2020.2           151.1331 0.0000 0.0225 73.2002 3.7314
## Moore et al., 2017              147.6524 0.0000 0.0214 72.2692 3.6061
## O'Brien et al., 2022            150.3460 0.0000 0.0218 72.7381 3.6681
## Petzel & Casad, 2022.1          113.1970 0.0003 0.0068 45.3167 1.8287
## Petzel & Casad, 2022.2          150.4113 0.0000 0.0219 72.7981 3.6762
## Sammy et al., 2017              150.0421 0.0000 0.0217 72.6139 3.6515
## Smith et al., 2022.1            148.3529 0.0000 0.0209 71.9580 3.5661
## Smith et al., 2022.2            147.3424 0.0000 0.0205 71.5968 3.5207
## Smith et al., 2022.3            142.4886 0.0000 0.0188 69.7777 3.3088
## Snijdewint & Scheepers, 2023    151.5308 0.0000 0.0225 73.2258 3.7349
## Trotman et al., 2018.2          145.9160 0.0000 0.0207 71.5844 3.5192
## Wood et al., 2018               151.3417 0.0000 0.0220 73.0045 3.7043
## Scheepers & Keller, 2022        150.9690 0.0000 0.0226 73.1537 3.7249
## Bosshard et al., 2023.2         150.9722 0.0000 0.0226 73.1250 3.7209
## Simms, 2022.1                   151.0057 0.0000 0.0220 72.9651 3.6989
## Simms, 2022.2                   149.5031 0.0000 0.0213 72.3604 3.6180
## Simms, 2022.3                   151.5156 0.0000 0.0221 73.0622 3.7123
## Simms, 2022.4                   149.2972 0.0000 0.0214 72.4551 3.6304
## Simms, 2022.5                   151.4207 0.0000 0.0221 73.0272 3.7074
## Simms, 2022.6                   151.6821 0.0000 0.0222 73.1412 3.7232
```

```
# Egger#
model_fun_CO_OutEgger <- rma.mv(yi = CO_cor_Z, V = CO_cor_Z_var, random = ~ 1 | paper_id/effect_size_id, mod = ~sqrt(CO_cor_Z_var), tdist=TRUE, data = Data)
```

```
## Warning: 94 rows with NAs omitted from model fitting.
```

```
model_fun_CO_OutEgger
```

```
##
## Multivariate Meta-Analysis Model (k = 68; method: REML)
##
## Variance Components:
##
##             estim    sqrt  nlvls  fixed                   factor
## sigma^2.1  0.0154  0.1241     28     no                 paper_id
## sigma^2.2  0.0113  0.1064     68     no  paper_id/effect_size_id
##
## Test for Residual Heterogeneity:
## QE(df = 66) = 150.9737, p-val < .0001
##
## Test of Moderators (coefficient 2):
## F(df1 = 1, df2 = 66) = 0.0342, p-val = 0.8539
##
## Model Results:
##
##                     estimate      se    tval  df    pval    ci.lb   ci.ub
## intrcpt               0.0472  0.0872  0.5410  66  0.5904  -0.1269  0.2213
## sqrt(CO_cor_Z_var)    0.1167  0.6310  0.1849  66  0.8539  -1.1432  1.3766
##
## ---
## Signif. codes:  0 '***' 0.001 '**' 0.01 '*' 0.05 '.' 0.1 ' ' 1
```

```
# Run trim-and-fill analysis for the right side
model_fun_CO_tf_right <- trimfill(model_fun_CO, side = "right")

# Run trim-and-fill analysis for the left side
model_fun_CO_tf_left <- trimfill(model_fun_CO, side = "left")

# Print the trim-and-fill model results
print(model_fun_CO_tf_right)
```

```
##
## Estimated number of missing studies on the right side: 13 (SE = 5.4346)
##
## Random-Effects Model (k = 81; tau^2 estimator: REML)
##
## tau^2 (estimated amount of total heterogeneity): 0.0342 (SE = 0.0088)
## tau (square root of estimated tau^2 value):      0.1848
## I^2 (total heterogeneity / total variability):   78.76%
## H^2 (total variability / sampling variability):  4.71
##
## Test for Heterogeneity:
## Q(df = 80) = 224.7476, p-val < .0001
##
## Model Results:
##
## estimate      se    zval    pval   ci.lb   ci.ub
##   0.1032  0.0266  3.8850  0.0001  0.0511  0.1553  ***
##
## ---
## Signif. codes:  0 '***' 0.001 '**' 0.01 '*' 0.05 '.' 0.1 ' ' 1
```

```r
print(model_fun_CO_tf_left)
```

```
##
## Estimated number of missing studies on the left side: 0 (SE = 4.4280)
##
## Random-Effects Model (k = 68; tau^2 estimator: REML)
##
## tau^2 (estimated amount of total heterogeneity): 0.0216 (SE = 0.0070)
## tau (square root of estimated tau^2 value):      0.1470
## I^2 (total heterogeneity / total variability):   72.40%
## H^2 (total variability / sampling variability):  3.62
##
## Test for Heterogeneity:
## Q(df = 67) = 151.6929, p-val < .0001
##
## Model Results:
##
## estimate      se    zval    pval    ci.lb   ci.ub
##   0.0473  0.0251  1.8842  0.0595  -0.0019  0.0964  .
##
## ---
## Signif. codes:  0 '***' 0.001 '**' 0.01 '*' 0.05 '.' 0.1 ' ' 1
```

```r
# Combine the number of studies imputed from both sides
total_imputed_studies <- model_fun_CO_tf_right$k0 + model_fun_CO_tf_left$k0
cat("Total number of imputed studies (both sides) for CO:", total_imputed_studies,
"\n")
```

```
## Total number of imputed studies (both sides) for CO: 13
```

```r
# Generate funnel plots only for right side

#par(mfrow=c(1, 3))
#funnel(model_fun_CO, main="Original Model")
funnel(model_fun_CO_tf_right, main="Trim-and-Fill Right", xlab = 'CO')
```

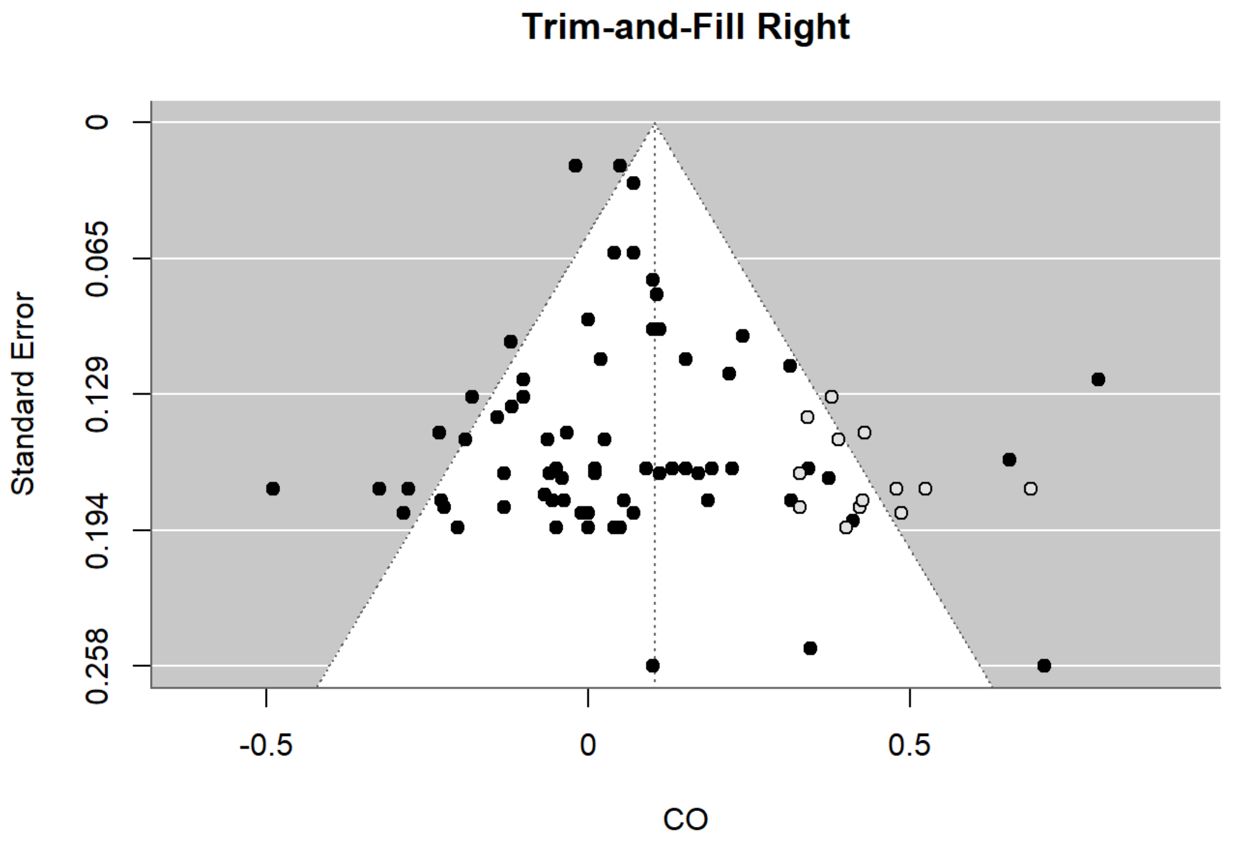


```
#funnel(model_fun_CO_tf_left, main="Trim-and-Fill Left")
```

5.2 Multilevel Model

```
model_CO_multilevel <- rma.mv(yi = CO_cor_Z, V = CO_cor_Z_var, slab = Ref_APA, data = D
ata, random = ~ 1 | paper_id/effect_size_id, test = "t", method = "REML")
```

```
## Warning: 94 rows with NAs omitted from model fitting.
```

```
model_CO_multilevel
```

```
##
## Multivariate Meta-Analysis Model (k = 68; method: REML)
##
## Variance Components:
##
##              estim    sqrt  nlvls  fixed                factor
## sigma^2.1  0.0134  0.1158     28     no              paper_id
## sigma^2.2  0.0114  0.1068     68     no  paper_id/effect_size_id
##
## Test for Heterogeneity:
## Q(df = 67) = 151.6929, p-val < .0001
##
## Model Results:
##
## estimate      se    tval  df    pval    ci.lb   ci.ub
##   0.0610  0.0325  1.8779  67  0.0647  -0.0038  0.1258  .
##
## ---
## Signif. codes:  0 '***' 0.001 '**' 0.01 '*' 0.05 '.' 0.1 ' ' 1
```

```
convert_z2r(0.0919)
```

```
## [1] 0.09164215
```

```
forest.rma(model_CO_multilevel, header = "CO",slab = Data$Ref_APA, alim=c(-0.8,1.5))
```

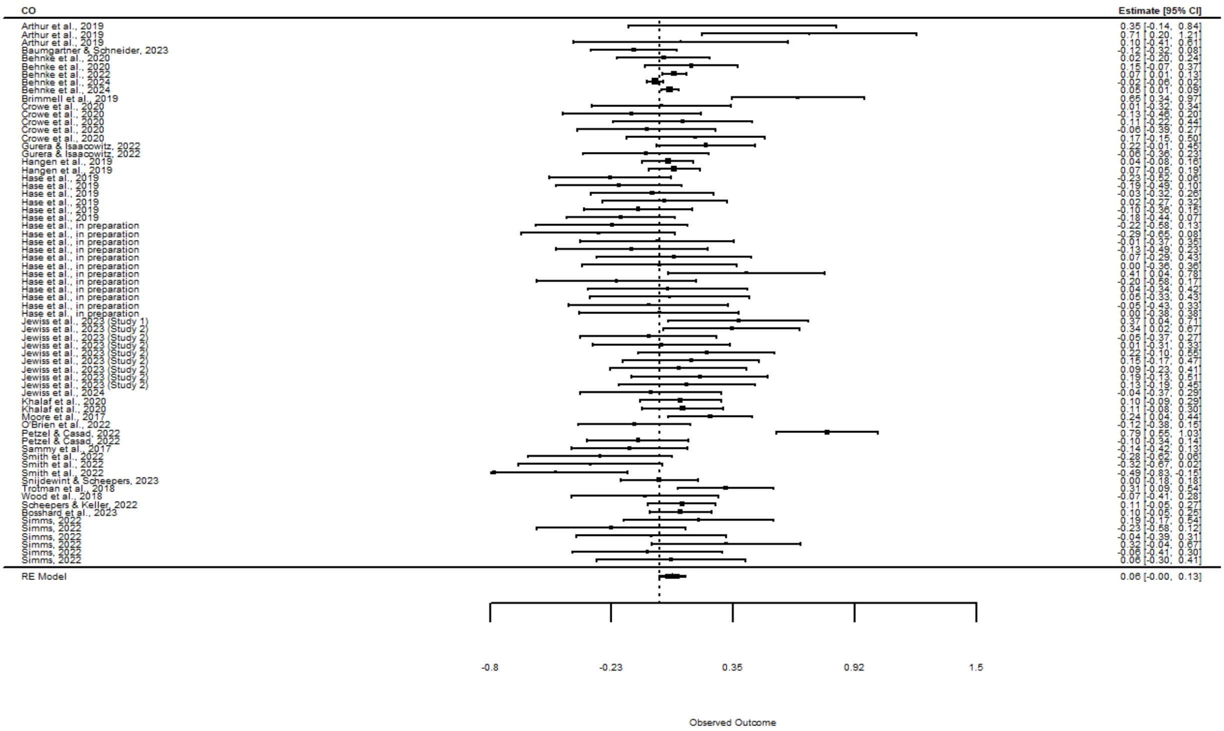

```
# Prediction Intervals

predict_CO <- predict(model_CO_multilevel, digits=3, transf=transf.ztor, level = 95)
predict_CO
```

```
##
##    pred  ci.lb ci.ub  pi.lb pi.ub
##   0.061 -0.004 0.125 -0.254 0.364
```

```
# Additional Parameters for the Multilevel Model


######## list 1 ########
list_CO <- Data$CO_cor_Z_var

############ sum 1#####################
sum_CO <- sum(list_CO, na.rm = TRUE)

###################### sum 2 #####################
sum2_CO <- (sum_CO)^2

####################### list 2 ##############
list_In_CO <- Data$CO_cor_Z_var_Sq

#################### sum 3 #######################
sum_In_CO<- sum(list_In_CO, na.rm = TRUE)

############### numerator ##############
numerator_CO<- (model_CO_multilevel$k-1)*sum_CO

############# denominator #############
denominator_CO<- sum2_CO - sum_In_CO

############## eps ################
EPS_CO<- numerator_CO / denominator_CO
EPS_CO
```

```
## [1] 39.89371
```

```
############### i2 1 level ##################
I2_1_CO <- (EPS_CO) / (model_CO_multilevel$sigma2[1] + model_CO_multilevel$sigma2[2] +
EPS_CO) *100
I2_1_CO
```

```
## [1] 99.93783
```

```
############## i2 2 level #################
I2_2_CO <- (model_CO_multilevel$sigma2[1]) / (model_CO_multilevel$sigma2[1] + model_CO_
multilevel$sigma2[2] + EPS_CO) *100
I2_2_CO
```

```
## [1] 0.03357787
```

```
########### I2 level 3
I2_3_CO <- (model_CO_multilevel$sigma2[2]) / (model_CO_multilevel$sigma2[1] + model_CO_
multilevel$sigma2[2] + EPS_CO) *100
I2_3_CO
```

```
## [1] 0.0285909
```

```
############### ML without level 2 ##########
model_CO_multilevel_2 <- rma.mv(yi = CO_cor_Z, V = CO_cor_Z_var, slab = Ref_APA, random
= ~ 1 | paper_id/effect_size_id, test = "t", method = "REML", sigma2=c(0,NA), tdist=TRU
E,data = Data)
```

```
## Warning: 94 rows with NAs omitted from model fitting.
```

```
############# ml without level 3 ###########
model_CO_multilevel_3 <- rma.mv(yi = CO_cor_Z, V = CO_cor_Z_var, slab = Ref_APA, random
= ~ 1 | paper_id/effect_size_id, test = "t", method = "REML", sigma2=c(NA,0), tdist=TRU
E,data = Data)
```

```
## Warning: 94 rows with NAs omitted from model fitting.
```

```
##################### sig level 2 #######################
anova12_CO <- anova(model_CO_multilevel,model_CO_multilevel_2)
anova12_CO
```

```
##
##         df      AIC     BIC     AICc logLik    LRT   pval       QE
## Full     3 -10.5180 -3.9039 -10.1371 8.2590               151.6929
## Reduced  2  -9.5398 -5.1304  -9.3523 6.7699 2.9782 0.0844 151.6929
```

```
###############sig level 3v #################
anova13_CO <- anova(model_CO_multilevel,model_CO_multilevel_3)
anova13_CO
```

```
##
##         df      AIC     BIC     AICc logLik    LRT   pval       QE
## Full     3 -10.5180 -3.9039 -10.1371 8.2590               151.6929
## Reduced  2  -4.7673 -0.3579  -4.5798 4.3836 7.7507 0.0054 151.6929
```

6. Total Peripheral Resistance

6.1 Identyfing outliers

```
model_fun_TPR <- rma(yi = TPR_cor_Z, vi= TPR_cor_Z_var, data = Data, slab = Ref_APA)
```

```
## Warning: 101 studies with NAs omitted from model fitting.
```

```
model_fun_TPR
```

```
##
## Random-Effects Model (k = 61; tau^2 estimator: REML)
##
## tau^2 (estimated amount of total heterogeneity): 0.0130 (SE = 0.0060)
## tau (square root of estimated tau^2 value):      0.1139
## I^2 (total heterogeneity / total variability):   49.04%
## H^2 (total variability / sampling variability):  1.96
##
## Test for Heterogeneity:
## Q(df = 60) = 117.8564, p-val < .0001
##
## Model Results:
##
## estimate      se     zval    pval    ci.lb    ci.ub
##  -0.0799  0.0240  -3.3272  0.0009  -0.1270  -0.0328  ***
##
## ---
## Signif. codes:  0 '***' 0.001 '**' 0.01 '*' 0.05 '.' 0.1 ' ' 1
```

```
# outliers diagnostics  - outlier rstudent > 3.0 #
Out_model_fun_TPR <- influence(model_fun_TPR)
Out_model_fun_TPR
```

```
##
##                                 rstudent  dffits cook.d  cov.r tau2.del
## Arthur et al., 2019.1            -1.1454 -0.1023 0.0104 1.0036   0.0128
## Arthur et al., 2019.2            -0.5555 -0.0455 0.0021 1.0117   0.0131
## Arthur et al., 2019.3             0.7127  0.0634 0.0040 1.0123   0.0131
## Baumgartner & Schneider, 2023     0.0651  0.0216 0.0005 1.0501   0.0137
## Behnke et al., 2024.1             1.2704  0.2657 0.0668 1.0211   0.0122
## Brimmell et al., 2019            -2.5624 -0.3573 0.1197 0.9319   0.0106
## Crowe et al., 2020.1             -0.3022 -0.0304 0.0009 1.0272   0.0133
## Crowe et al., 2020.2              0.2982  0.0425 0.0018 1.0286   0.0134
## Crowe et al., 2020.3              0.8475  0.1047 0.0110 1.0206   0.0131
## Crowe et al., 2020.4              0.0994  0.0189 0.0004 1.0294   0.0134
## Crowe et al., 2020.5              0.5967  0.0768 0.0059 1.0253   0.0133
## Hangen et al., 2019.1             0.5387  0.1113 0.0130 1.0616   0.0137
## Hangen et al., 2019.2             0.6168  0.1247 0.0162 1.0587   0.0137
## Hase et al., 2019.1               2.2108  0.2551 0.0620 0.9615   0.0114
## Hase et al., 2019.2               1.2757  0.1600 0.0254 1.0094   0.0128
## Hase et al., 2019.3               0.6282  0.0874 0.0077 1.0290   0.0133
## Hase et al., 2019.4               0.2224  0.0363 0.0013 1.0331   0.0135
## Hase et al., 2019.5               0.5259  0.0811 0.0067 1.0356   0.0134
## Hase et al., 2019.6               0.5942  0.0900 0.0082 1.0343   0.0134
## Hase et al., in preparation.3     0.1865  0.0271 0.0007 1.0257   0.0134
## Hase et al., in preparation.4    -0.1398 -0.0099 0.0001 1.0246   0.0133
## Hase et al., in preparation.5    -0.0933 -0.0046 0.0000 1.0248   0.0133
## Hase et al., in preparation.6     0.7473  0.0877 0.0077 1.0201   0.0132
## Hase et al., in preparation.7     1.4549  0.1558 0.0241 1.0020   0.0127
## Hase et al., in preparation.8    -0.0468  0.0007 0.0000 1.0250   0.0133
## Hase et al., in preparation.9    -1.5782 -0.1818 0.0326 0.9913   0.0124
## Hase et al., in preparation.10    0.4486  0.0536 0.0029 1.0223   0.0133
## Hase et al., in preparation.11   -1.0838 -0.1189 0.0141 1.0076   0.0128
## Hase et al., in preparation.12    0.6286  0.0722 0.0052 1.0203   0.0132
## Hase et al., in preparation.13    0.6286  0.0722 0.0052 1.0203   0.0132
## Hase et al., in preparation.14   -0.6451 -0.0671 0.0045 1.0175   0.0131
## Jewiss et al., 2023 (Study 1)    -1.9260 -0.2474 0.0594 0.9731   0.0118
## Jewiss et al., 2023 (Study 2).1  -0.6691 -0.0779 0.0061 1.0215   0.0132
## Jewiss et al., 2023 (Study 2).2  -0.5116 -0.0574 0.0033 1.0247   0.0133
## Jewiss et al., 2023 (Study 2).3   0.1505  0.0253 0.0006 1.0298   0.0134
## Jewiss et al., 2023 (Study 2).4  -0.5116 -0.0574 0.0033 1.0247   0.0133
## Jewiss et al., 2023 (Study 2).5  -0.2029 -0.0181 0.0003 1.0287   0.0134
## Jewiss et al., 2023 (Study 2).6  -0.2029 -0.0181 0.0003 1.0287   0.0134
## Jewiss et al., 2023 (Study 2).7   0.6023  0.0782 0.0062 1.0257   0.0133
## Jewiss et al., 2023 (Study 2).8   0.9066  0.1121 0.0126 1.0195   0.0131
## Jewiss et al., 2024               0.5910  0.0754 0.0057 1.0249   0.0133
## Khalaf et al., 2020.1             0.0270  0.0162 0.0003 1.0529   0.0138
## Khalaf et al., 2020.2            -0.1737 -0.0166 0.0003 1.0515   0.0137
## Moore et al., 2017               -1.6109 -0.2845 0.0763 0.9802   0.0116
## O'Brien et al., 2022             -1.3518 -0.1951 0.0374 0.9997   0.0124
## Petzel & Casad, 2022.1           -3.6025 -0.7086 0.3951 0.8075   0.0070
## Petzel & Casad, 2022.2           -0.1086 -0.0062 0.0000 1.0423   0.0136
## Sammy et al., 2017                1.6013  0.2029 0.0403 0.9948   0.0123
## Smith et al., 2022.1             -0.9429 -0.1098 0.0121 1.0131   0.0130
## Smith et al., 2022.2             -0.5772 -0.0635 0.0041 1.0216   0.0132
## Smith et al., 2022.3             -1.3001 -0.1565 0.0243 1.0015   0.0126
## Trotman et al., 2018.2           -1.3110 -0.2069 0.0419 1.0024   0.0124
## Wood et al., 2018                 0.5727  0.0710 0.0051 1.0237   0.0133
```

```
## Scheepers & Keller, 2022          0.4492  0.0885 0.0081 1.0557   0.0137
## Bosshard et al., 2023.2           0.5133  0.1017 0.0108 1.0569   0.0137
## Simms, 2022.1                    -0.1623 -0.0126 0.0002 1.0257   0.0133
## Simms, 2022.2                     1.2756  0.1423 0.0202 1.0078   0.0128
## Simms, 2022.3                     0.2972  0.0399 0.0016 1.0258   0.0133
## Simms, 2022.4                    -0.9424 -0.1073 0.0115 1.0125   0.0130
## Simms, 2022.5                     0.5332  0.0659 0.0044 1.0237   0.0133
## Simms, 2022.6                     0.4199  0.0535 0.0029 1.0249   0.0133
##                                   QE.del    hat weight    dfbs inf
## Arthur et al., 2019.1           115.6011 0.0076 0.7646 -0.1024
## Arthur et al., 2019.2           117.1407 0.0072 0.7246 -0.0454
## Arthur et al., 2019.3           117.5669 0.0072 0.7246  0.0633
## Baumgartner & Schneider, 2023   117.6039 0.0242 2.4209  0.0216
## Behnke et al., 2024.1            92.0135 0.0431 4.3115  0.2616
## Brimmell et al., 2019           106.2089 0.0149 1.4947 -0.3610
## Crowe et al., 2020.1            117.3103 0.0142 1.4163 -0.0303
## Crowe et al., 2020.2            117.8563 0.0142 1.4163  0.0424
## Crowe et al., 2020.3            117.4306 0.0142 1.4163  0.1046
## Crowe et al., 2020.4            117.7928 0.0142 1.4163  0.0188
## Crowe et al., 2020.5            117.7337 0.0142 1.4163  0.0767
## Hangen et al., 2019.1           117.8382 0.0344 3.4352  0.1124
## Hangen et al., 2019.2           117.7661 0.0344 3.4352  0.1258
## Hase et al., 2019.1             112.5643 0.0166 1.6627  0.2561
## Hase et al., 2019.2             116.4717 0.0162 1.6167  0.1600
## Hase et al., 2019.3             117.7173 0.0166 1.6627  0.0874
## Hase et al., 2019.4             117.8387 0.0162 1.6167  0.0362
## Hase et al., 2019.5             117.8068 0.0193 1.9289  0.0811
## Hase et al., 2019.6             117.7582 0.0193 1.9289  0.0900
## Hase et al., in preparation.3   117.8420 0.0125 1.2463  0.0271
## Hase et al., in preparation.4   117.6082 0.0122 1.2161 -0.0099
## Hase et al., in preparation.5   117.6596 0.0122 1.2161 -0.0046
## Hase et al., in preparation.6   117.5648 0.0125 1.2463  0.0876
## Hase et al., in preparation.7   115.9999 0.0122 1.2161  0.1561
## Hase et al., in preparation.8   117.7050 0.0122 1.2161  0.0007
## Hase et al., in preparation.9   113.2293 0.0119 1.1854 -0.1826
## Hase et al., in preparation.10  117.8169 0.0115 1.1540  0.0535
## Hase et al., in preparation.11  115.3756 0.0115 1.1540 -0.1190
## Hase et al., in preparation.12  117.6904 0.0115 1.1540  0.0721
## Hase et al., in preparation.13  117.6904 0.0115 1.1540  0.0721
## Hase et al., in preparation.14  116.7107 0.0115 1.1540 -0.0671
## Jewiss et al., 2023 (Study 1)   110.8684 0.0139 1.3892 -0.2488
## Jewiss et al., 2023 (Study 2).1 116.4399 0.0144 1.4429 -0.0778
## Jewiss et al., 2023 (Study 2).2 116.8568 0.0144 1.4429 -0.0573
## Jewiss et al., 2023 (Study 2).3 117.8186 0.0144 1.4429  0.0252
## Jewiss et al., 2023 (Study 2).4 116.8568 0.0144 1.4429 -0.0573
## Jewiss et al., 2023 (Study 2).5 117.4657 0.0144 1.4429 -0.0181
## Jewiss et al., 2023 (Study 2).6 117.4657 0.0144 1.4429 -0.0181
## Jewiss et al., 2023 (Study 2).7 117.7303 0.0144 1.4429  0.0781
## Jewiss et al., 2023 (Study 2).8 117.3334 0.0144 1.4429  0.1121
## Jewiss et al., 2024             117.7372 0.0139 1.3892  0.0753
## Khalaf et al., 2020.1           117.5001 0.0256 2.5553  0.0163
## Khalaf et al., 2020.2           117.0312 0.0256 2.5553 -0.0166
## Moore et al., 2017              109.1560 0.0248 2.4792 -0.2826
## O'Brien et al., 2022            112.9972 0.0185 1.8526 -0.1952
## Petzel & Casad, 2022.1           94.5681 0.0207 2.0689 -0.7069   *
## Petzel & Casad, 2022.2          117.4207 0.0207 2.0689 -0.0062
```

```
## Sammy et al., 2017             115.3210 0.0177 1.7715  0.2031
## Smith et al., 2022.1           115.6715 0.0133 1.3336 -0.1098
## Smith et al., 2022.2           116.7635 0.0133 1.3336 -0.0634
## Smith et al., 2022.3           114.2596 0.0133 1.3336 -0.1568
## Trotman et al., 2018.2         112.3381 0.0219 2.1941 -0.2066
## Wood et al., 2018              117.7475 0.0131 1.3051  0.0709
## Scheepers & Keller, 2022       117.8562 0.0294 2.9391  0.0891
## Bosshard et al., 2023.2        117.8441 0.0311 3.1102  0.1025
## Simms, 2022.1                  117.5671 0.0128 1.2760 -0.0126
## Simms, 2022.2                  116.5156 0.0128 1.2760  0.1424
## Simms, 2022.3                  117.8564 0.0128 1.2760  0.0398
## Simms, 2022.4                  115.7351 0.0128 1.2760 -0.1073
## Simms, 2022.5                  117.7748 0.0128 1.2760  0.0657
## Simms, 2022.6                  117.8334 0.0128 1.2760  0.0534
```

```
ranktest(model_fun_TPR)
```

```
## Warning in cor.test.default(yi.star, vi, method = "kendall", exact = exact):
## nie można obliczyć dokładnej wartości prawdopodobieństwa z powtórzonymi
## wartościami
```

```
##
## Rank Correlation Test for Funnel Plot Asymmetry
##
## Kendall's tau = -0.0117, p = 0.8957
```

```
# leave one out analysis #

leave1out(model_fun_TPR)
```

```
## 
##                                  estimate     se    zval   pval   ci.lb   ci.ub 
## Arthur et al., 2019.1             -0.0775 0.0241 -3.2192 0.0013 -0.1246 -0.0303 
## Arthur et al., 2019.2             -0.0788 0.0242 -3.2626 0.0011 -0.1262 -0.0315 
## Arthur et al., 2019.3             -0.0815 0.0242 -3.3701 0.0008 -0.1288 -0.0341 
## Baumgartner & Schneider, 2023     -0.0805 0.0246 -3.2681 0.0011 -0.1287 -0.0322 
## Behnke et al., 2024.1             -0.0861 0.0243 -3.5484 0.0004 -0.1337 -0.0386 
## Brimmell et al., 2019             -0.0716 0.0232 -3.0882 0.0020 -0.1171 -0.0262 
## Crowe et al., 2020.1              -0.0792 0.0243 -3.2527 0.0011 -0.1269 -0.0315 
## Crowe et al., 2020.2              -0.0810 0.0244 -3.3227 0.0009 -0.1287 -0.0332 
## Crowe et al., 2020.3              -0.0824 0.0243 -3.3973 0.0007 -0.1300 -0.0349 
## Crowe et al., 2020.4              -0.0804 0.0244 -3.2981 0.0010 -0.1281 -0.0326 
## Crowe et al., 2020.5              -0.0818 0.0243 -3.3620 0.0008 -0.1295 -0.0341 
## Hangen et al., 2019.1             -0.0827 0.0248 -3.3396 0.0008 -0.1312 -0.0341 
## Hangen et al., 2019.2             -0.0830 0.0247 -3.3574 0.0008 -0.1314 -0.0345 
## Hase et al., 2019.1               -0.0859 0.0236 -3.6472 0.0003 -0.1321 -0.0397 
## Hase et al., 2019.2               -0.0838 0.0241 -3.4704 0.0005 -0.1311 -0.0365 
## Hase et al., 2019.3               -0.0820 0.0244 -3.3666 0.0008 -0.1298 -0.0343 
## Hase et al., 2019.4               -0.0808 0.0244 -3.3093 0.0009 -0.1287 -0.0329 
## Hase et al., 2019.5               -0.0819 0.0244 -3.3497 0.0008 -0.1298 -0.0340 
## Hase et al., 2019.6               -0.0821 0.0244 -3.3608 0.0008 -0.1300 -0.0342 
## Hase et al., in preparation.3     -0.0806 0.0243 -3.3121 0.0009 -0.1283 -0.0329 
## Hase et al., in preparation.4     -0.0797 0.0243 -3.2772 0.0010 -0.1273 -0.0320 
## Hase et al., in preparation.5     -0.0798 0.0243 -3.2821 0.0010 -0.1275 -0.0322 
## Hase et al., in preparation.6     -0.0820 0.0243 -3.3813 0.0007 -0.1296 -0.0345 
## Hase et al., in preparation.7     -0.0837 0.0240 -3.4790 0.0005 -0.1308 -0.0365 
## Hase et al., in preparation.8     -0.0799 0.0243 -3.2870 0.0010 -0.1276 -0.0323 
## Hase et al., in preparation.9     -0.0756 0.0239 -3.1604 0.0016 -0.1225 -0.0287 
## Hase et al., in preparation.10    -0.0812 0.0243 -3.3439 0.0008 -0.1288 -0.0336 
## Hase et al., in preparation.11    -0.0771 0.0241 -3.1964 0.0014 -0.1243 -0.0298 
## Hase et al., in preparation.12    -0.0817 0.0243 -3.3655 0.0008 -0.1292 -0.0341 
## Hase et al., in preparation.13    -0.0817 0.0243 -3.3655 0.0008 -0.1292 -0.0341 
## Hase et al., in preparation.14    -0.0783 0.0242 -3.2318 0.0012 -0.1258 -0.0308 
## Jewiss et al., 2023 (Study 1)     -0.0741 0.0237 -3.1258 0.0018 -0.1205 -0.0276 
## Jewiss et al., 2023 (Study 2).1   -0.0780 0.0243 -3.2148 0.0013 -0.1256 -0.0305 
## Jewiss et al., 2023 (Study 2).2   -0.0785 0.0243 -3.2300 0.0012 -0.1262 -0.0309 
## Jewiss et al., 2023 (Study 2).3   -0.0805 0.0244 -3.3037 0.0010 -0.1283 -0.0328 
## Jewiss et al., 2023 (Study 2).4   -0.0785 0.0243 -3.2300 0.0012 -0.1262 -0.0309 
## Jewiss et al., 2023 (Study 2).5   -0.0795 0.0244 -3.2624 0.0011 -0.1272 -0.0317 
## Jewiss et al., 2023 (Study 2).6   -0.0795 0.0244 -3.2624 0.0011 -0.1272 -0.0317 
## Jewiss et al., 2023 (Study 2).7   -0.0818 0.0243 -3.3628 0.0008 -0.1295 -0.0341 
## Jewiss et al., 2023 (Study 2).8   -0.0826 0.0243 -3.4064 0.0007 -0.1302 -0.0351 
## Jewiss et al., 2024               -0.0817 0.0243 -3.3612 0.0008 -0.1294 -0.0341 
## Khalaf et al., 2020.1             -0.0803 0.0246 -3.2587 0.0011 -0.1286 -0.0320 
## Khalaf et al., 2020.2             -0.0795 0.0246 -3.2283 0.0012 -0.1278 -0.0312 
## Moore et al., 2017                -0.0733 0.0238 -3.0816 0.0021 -0.1199 -0.0267 
## O'Brien et al., 2022              -0.0753 0.0240 -3.1343 0.0017 -0.1224 -0.0282 
## Petzel & Casad, 2022.1            -0.0648 0.0216 -3.0031 0.0027 -0.1071 -0.0225 
## Petzel & Casad, 2022.2            -0.0798 0.0245 -3.2529 0.0011 -0.1278 -0.0317 
## Sammy et al., 2017                -0.0847 0.0240 -3.5372 0.0004 -0.1317 -0.0378 
## Smith et al., 2022.1              -0.0773 0.0242 -3.1965 0.0014 -0.1247 -0.0299 
## Smith et al., 2022.2              -0.0784 0.0243 -3.2288 0.0012 -0.1260 -0.0308 
## Smith et al., 2022.3              -0.0762 0.0240 -3.1690 0.0015 -0.1233 -0.0291 
## Trotman et al., 2018.2            -0.0750 0.0241 -3.1189 0.0018 -0.1221 -0.0279 
## Wood et al., 2018                 -0.0816 0.0243 -3.3588 0.0008 -0.1293 -0.0340 
```

```
## Scheepers & Keller, 2022        -0.0821 0.0247 -3.3261 0.0009 -0.1305 -0.0337
## Bosshard et al., 2023.2         -0.0824 0.0247 -3.3373 0.0008 -0.1308 -0.0340
## Simms, 2022.1                   -0.0796 0.0243 -3.2728 0.0011 -0.1273 -0.0319
## Simms, 2022.2                   -0.0833 0.0241 -3.4558 0.0005 -0.1306 -0.0361
## Simms, 2022.3                   -0.0809 0.0243 -3.3246 0.0009 -0.1286 -0.0332
## Simms, 2022.4                   -0.0773 0.0242 -3.1999 0.0014 -0.1247 -0.0300
## Simms, 2022.5                   -0.0815 0.0243 -3.3538 0.0008 -0.1291 -0.0339
## Simms, 2022.6                   -0.0812 0.0243 -3.3396 0.0008 -0.1289 -0.0336
##                                       Q     Qp   tau2      I2     H2
## Arthur et al., 2019.1          115.6011 0.0000 0.0128 49.1178 1.9653
## Arthur et al., 2019.2          117.1407 0.0000 0.0131 49.6122 1.9846
## Arthur et al., 2019.3          117.5669 0.0000 0.0131 49.6447 1.9859
## Baumgartner & Schneider, 2023   117.6039 0.0000 0.0137 50.2527 2.0102
## Behnke et al., 2024.1           92.0135 0.0038 0.0122 38.7534 1.6327
## Brimmell et al., 2019          106.2089 0.0002 0.0106 44.0855 1.7884
## Crowe et al., 2020.1           117.3103 0.0000 0.0133 49.9444 1.9978
## Crowe et al., 2020.2           117.8563 0.0000 0.0134 50.0218 2.0009
## Crowe et al., 2020.3           117.4306 0.0000 0.0131 49.5710 1.9830
## Crowe et al., 2020.4           117.7928 0.0000 0.0134 50.0627 2.0025
## Crowe et al., 2020.5           117.7337 0.0000 0.0133 49.8377 1.9935
## Hangen et al., 2019.1          117.8382 0.0000 0.0137 49.1716 1.9674
## Hangen et al., 2019.2          117.7661 0.0000 0.0137 49.0087 1.9611
## Hase et al., 2019.1            112.5643 0.0000 0.0114 45.8380 1.8463
## Hase et al., 2019.2            116.4717 0.0000 0.0128 48.7662 1.9518
## Hase et al., 2019.3            117.7173 0.0000 0.0133 49.8336 1.9934
## Hase et al., 2019.4            117.8387 0.0000 0.0135 50.1028 2.0041
## Hase et al., 2019.5            117.8068 0.0000 0.0134 49.9603 1.9984
## Hase et al., 2019.6            117.7582 0.0000 0.0134 49.8840 1.9954
## Hase et al., in preparation.3   117.8420 0.0000 0.0134 49.9966 1.9999
## Hase et al., in preparation.4   117.6082 0.0000 0.0133 49.9588 1.9984
## Hase et al., in preparation.5   117.6596 0.0000 0.0133 49.9720 1.9989
## Hase et al., in preparation.6   117.5648 0.0000 0.0132 49.6817 1.9873
## Hase et al., in preparation.7   115.9999 0.0000 0.0127 48.6767 1.9484
## Hase et al., in preparation.8   117.7050 0.0000 0.0133 49.9821 1.9993
## Hase et al., in preparation.9   113.2293 0.0000 0.0124 48.0697 1.9257
## Hase et al., in preparation.10  117.8169 0.0000 0.0133 49.8813 1.9953
## Hase et al., in preparation.11  115.3756 0.0000 0.0128 49.0463 1.9626
## Hase et al., in preparation.12  117.6904 0.0000 0.0132 49.7712 1.9909
## Hase et al., in preparation.13  117.6904 0.0000 0.0132 49.7712 1.9909
## Hase et al., in preparation.14  116.7107 0.0000 0.0131 49.6099 1.9845
## Jewiss et al., 2023 (Study 1)   110.8684 0.0001 0.0118 46.8025 1.8798
## Jewiss et al., 2023 (Study 2).1 116.4399 0.0000 0.0132 49.6015 1.9842
## Jewiss et al., 2023 (Study 2).2 116.8568 0.0000 0.0133 49.7797 1.9912
## Jewiss et al., 2023 (Study 2).3 117.8186 0.0000 0.0134 50.0669 2.0027
## Jewiss et al., 2023 (Study 2).4 116.8568 0.0000 0.0133 49.7797 1.9912
## Jewiss et al., 2023 (Study 2).5 117.4657 0.0000 0.0134 50.0065 2.0003
## Jewiss et al., 2023 (Study 2).6 117.4657 0.0000 0.0134 50.0065 2.0003
## Jewiss et al., 2023 (Study 2).7 117.7303 0.0000 0.0133 49.8365 1.9935
## Jewiss et al., 2023 (Study 2).8 117.3334 0.0000 0.0131 49.4903 1.9798
## Jewiss et al., 2024            117.7372 0.0000 0.0133 49.8387 1.9936
## Khalaf et al., 2020.1          117.5001 0.0000 0.0138 50.2441 2.0098
## Khalaf et al., 2020.2          117.0312 0.0000 0.0137 50.1680 2.0067
## Moore et al., 2017             109.1560 0.0001 0.0116 46.1263 1.8562
## O'Brien et al., 2022           112.9972 0.0000 0.0124 47.9867 1.9226
## Petzel & Casad, 2022.1          94.5681 0.0023 0.0070 34.2352 1.5206
## Petzel & Casad, 2022.2         117.4207 0.0000 0.0136 50.1916 2.0077
```

```
## Sammy et al., 2017            115.3210 0.0000 0.0123 47.7699 1.9146
## Smith et al., 2022.1          115.6715 0.0000 0.0130 49.2172 1.9692
## Smith et al., 2022.2          116.7635 0.0000 0.0132 49.6983 1.9880
## Smith et al., 2022.3          114.2596 0.0000 0.0126 48.5472 1.9435
## Trotman et al., 2018.2        112.3381 0.0000 0.0124 47.7982 1.9156
## Wood et al., 2018             117.7475 0.0000 0.0133 49.8392 1.9936
## Scheepers & Keller, 2022      117.8562 0.0000 0.0137 49.8700 1.9948
## Bosshard et al., 2023.2       117.8441 0.0000 0.0137 49.6472 1.9860
## Simms, 2022.1                 117.5671 0.0000 0.0133 49.9714 1.9989
## Simms, 2022.2                 116.5156 0.0000 0.0128 48.9619 1.9593
## Simms, 2022.3                 117.8564 0.0000 0.0133 49.9799 1.9992
## Simms, 2022.4                 115.7351 0.0000 0.0130 49.2307 1.9697
## Simms, 2022.5                 117.7748 0.0000 0.0133 49.8618 1.9945
## Simms, 2022.6                 117.8334 0.0000 0.0133 49.9288 1.9972
```

```
# Egger#
model_fun_TPR_OutEgger <- rma.mv(yi = TPR_cor_Z, V = TPR_cor_Z_var, random = ~ 1 | pape
r_id/effect_size_id, mod = ~sqrt(TPR_cor_Z_var), tdist=TRUE, data = Data)
```

```
## Warning: 101 rows with NAs omitted from model fitting.
```

```
model_fun_TPR_OutEgger
```

```
##
## Multivariate Meta-Analysis Model (k = 61; method: REML)
##
## Variance Components:
##
##             estim    sqrt  nlvls  fixed                  factor
## sigma^2.1  0.0169  0.1299     24     no                paper_id
## sigma^2.2  0.0000  0.0000     61     no  paper_id/effect_size_id
##
## Test for Residual Heterogeneity:
## QE(df = 59) = 94.1233, p-val = 0.0025
##
## Test of Moderators (coefficient 2):
## F(df1 = 1, df2 = 59) = 1.3928, p-val = 0.2427
##
## Model Results:
##
##                      estimate      se     tval  df    pval    ci.lb   ci.ub
## intrcpt                0.0011  0.0901   0.0119  59  0.9906  -0.1793  0.1815
## sqrt(TPR_cor_Z_var)   -0.7579  0.6422  -1.1802  59  0.2427  -2.0430  0.5272
##
## ---
## Signif. codes:  0 '***' 0.001 '**' 0.01 '*' 0.05 '.' 0.1 ' ' 1
```

```r
# Run trim-and-fill analysis for the right side
model_fun_TPR_tf_right <- trimfill(model_fun_TPR, side = "right")

# Run trim-and-fill analysis for the left side
model_fun_TPR_tf_left <- trimfill(model_fun_TPR, side = "left")

# Print the trim-and-fill model results
print(model_fun_TPR_tf_right)
```

```
##
## Estimated number of missing studies on the right side: 0 (SE = 4.4372)
##
## Random-Effects Model (k = 61; tau^2 estimator: REML)
##
## tau^2 (estimated amount of total heterogeneity): 0.0130 (SE = 0.0060)
## tau (square root of estimated tau^2 value):      0.1139
## I^2 (total heterogeneity / total variability):   49.04%
## H^2 (total variability / sampling variability):  1.96
##
## Test for Heterogeneity:
## Q(df = 60) = 117.8564, p-val < .0001
##
## Model Results:
##
## estimate      se     zval    pval    ci.lb    ci.ub
##  -0.0799  0.0240  -3.3272  0.0009  -0.1270  -0.0328  ***
##
## ---
## Signif. codes:  0 '***' 0.001 '**' 0.01 '*' 0.05 '.' 0.1 ' ' 1
```

```r
print(model_fun_TPR_tf_left)
```

```
##
## Estimated number of missing studies on the left side: 4 (SE = 4.9501)
##
## Random-Effects Model (k = 65; tau^2 estimator: REML)
##
## tau^2 (estimated amount of total heterogeneity): 0.0174 (SE = 0.0068)
## tau (square root of estimated tau^2 value):      0.1321
## I^2 (total heterogeneity / total variability):   55.84%
## H^2 (total variability / sampling variability):  2.26
##
## Test for Heterogeneity:
## Q(df = 64) = 145.0705, p-val < .0001
##
## Model Results:
##
## estimate      se     zval    pval    ci.lb    ci.ub
##  -0.1015  0.0250  -4.0679  <.0001  -0.1505  -0.0526  ***
##
## ---
## Signif. codes:  0 '***' 0.001 '**' 0.01 '*' 0.05 '.' 0.1 ' ' 1
```

```
# Combine the number of studies imputed from both sides
total_imputed_studies <- model_fun_TPR_tf_right$k0 + model_fun_TPR_tf_left$k0
cat("Total number of imputed studies (both sides) for TPR:", total_imputed_studies,
"\n")
```

```
## Total number of imputed studies (both sides) for TPR: 4
```

```
# Generate funnel plots only lef
#par(mfrow=c(1, 3))
#funnel(model_fun_TPR, main="Original Model")
#funnel(model_fun_TPR_tf_right, main="Trim-and-Fill Right")
funnel(model_fun_TPR_tf_left, main="Trim-and-Fill Left",  xlab = 'TPR')
```

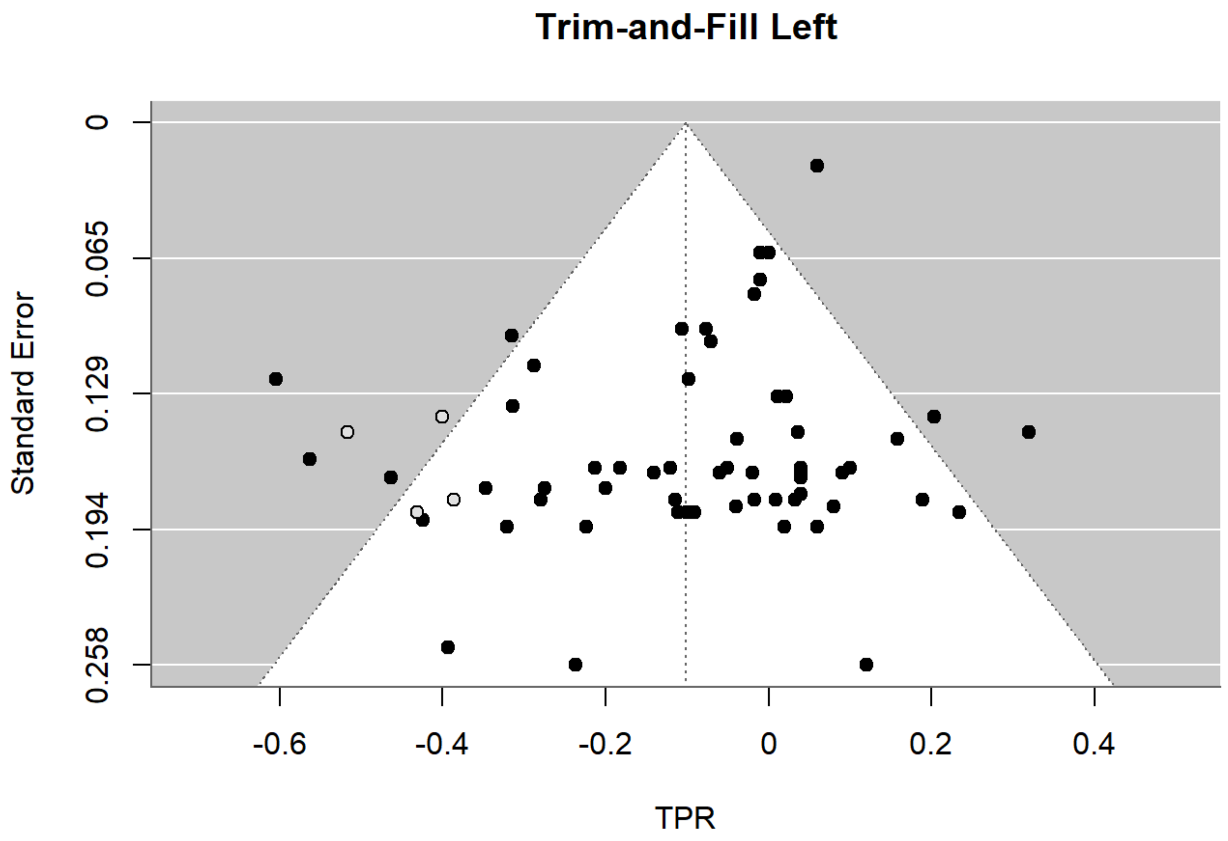


```
# Return to single plot layout
#par(mfrow=c(1, 1))
```

6.2 Multilevel Model

```
model_TPR_multilevel <- rma.mv(yi = TPR_cor_Z, V = TPR_cor_Z_var, slab = Ref_APA, data
= Data, random = ~ 1 | paper_id/effect_size_id, test = "t", method = "REML")
```

```
## Warning: 101 rows with NAs omitted from model fitting.
```

```
model_TPR_multilevel
```

```
##
## Multivariate Meta-Analysis Model (k = 61; method: REML)
##
## Variance Components:
##
##             estim    sqrt  nlvls  fixed                   factor
## sigma^2.1  0.0174  0.1319     24     no                 paper_id
## sigma^2.2  0.0000  0.0000     61     no  paper_id/effect_size_id
##
## Test for Heterogeneity:
## Q(df = 60) = 117.8564, p-val < .0001
##
## Model Results:
##
## estimate      se     tval  df    pval    ci.lb    ci.ub
##  -0.0980  0.0341  -2.8734  60  0.0056  -0.1662  -0.0298  **
##
## ---
## Signif. codes:  0 '***' 0.001 '**' 0.01 '*' 0.05 '.' 0.1 ' ' 1
```

```
convert_z2r(-0.1042)
```

```
## [1] -0.1038245
```

```
predict_TPR <- predict(model_TPR_multilevel, digits=3, transf=transf.ztor, level = 95)
predict_TPR
```

```
##
##    pred  ci.lb  ci.ub  pi.lb pi.ub
##  -0.098 -0.165 -0.030 -0.354 0.173
```

```
forest.rma(model_TPR_multilevel, header = "TPR",slab = Data$Ref_APA, alim=c(-1, 1))
```

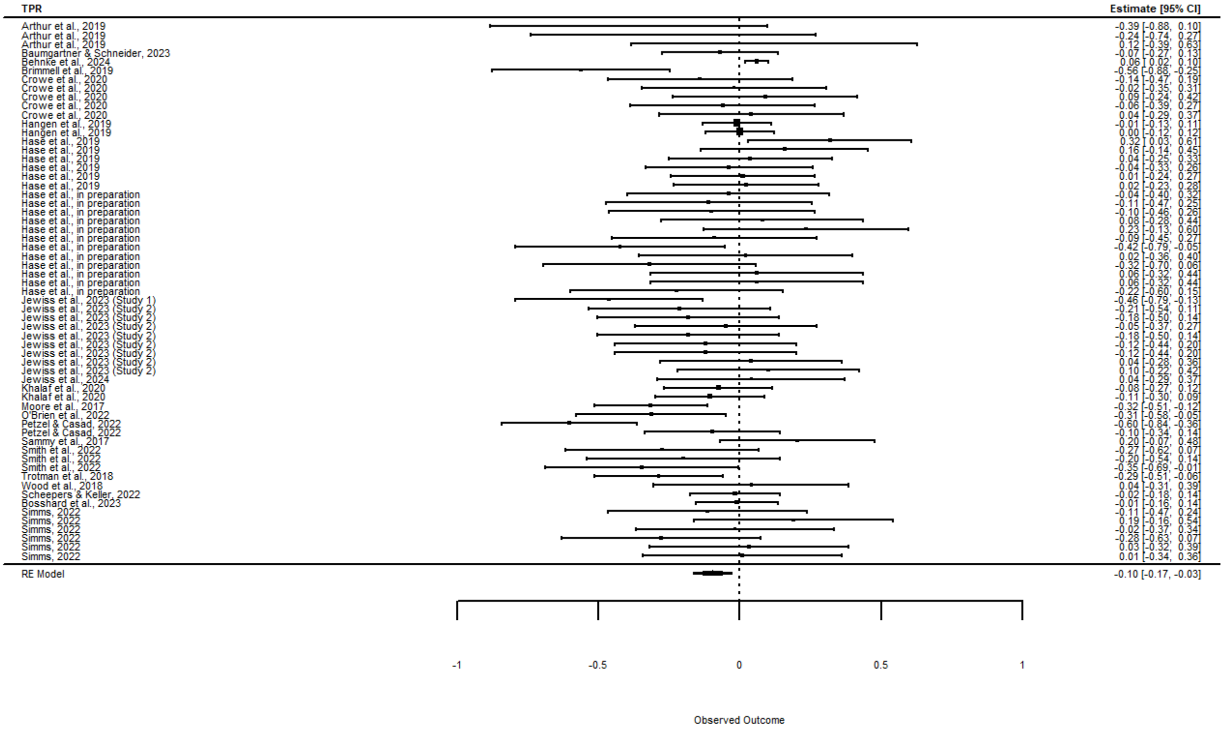

```
######## list 1 ########
list_TPR <- Data$TPR_cor_Z_var

############ sum 1######################
sum_TPR <- sum(list_TPR, na.rm = TRUE)

###################### sum 2 #####################
sum2_TPR <- (sum_TPR)^2

######################## list 2 ##############
list_In_TPR <- Data$TPR_cor_Z_var_Sq

#################### sum 3 ######################
sum_In_TPR<- sum(list_In_TPR, na.rm = TRUE)

################ numerator ##############
numerator_TPR<- (model_TPR_multilevel$k-1)*sum_TPR

############# denominator #############
denominator_TPR<- sum2_TPR - sum_In_TPR

############## eps ################
EPS_TPR<- numerator_TPR / denominator_TPR

############### i2 1 level ##################
I2_1_TPR <- (EPS_TPR) / (model_TPR_multilevel$sigma2[1] + model_TPR_multilevel$sigma2
[2] + EPS_TPR) *100
I2_1_TPR
```

```
## [1] 99.95343
```

```
############## i2 2 level #################
I2_2_TPR <- (model_TPR_multilevel$sigma2[1]) / (model_TPR_multilevel$sigma2[1] + model_
TPR_multilevel$sigma2[2] + EPS_TPR) *100
I2_2_TPR
```

```
## [1] 0.04656649
```

```
########### I2 level 3
I2_3_TPR <- (model_TPR_multilevel$sigma2[2]) / (model_TPR_multilevel$sigma2[1] + model_
TPR_multilevel$sigma2[2] + EPS_TPR) *100
I2_3_TPR
```

```
## [1] 6.142443e-11
```

```
############### ML without level 2 ##########
model_TPR_multilevel_2 <- rma.mv(yi = TPR_cor_Z, V = TPR_cor_Z_var, slab = Ref_APA, ran
dom = ~ 1 | paper_id/effect_size_id, test = "t", method = "REML", sigma2=c(0,NA), tdist
=TRUE,data = Data)
```

```
## Warning: 101 rows with NAs omitted from model fitting.
```

```
############# ml without level 3 ###########
model_TPR_multilevel_3 <- rma.mv(yi = TPR_cor_Z, V = TPR_cor_Z_var, slab = Ref_APA, ran
dom = ~ 1 | paper_id/effect_size_id, test = "t", method = "REML", sigma2=c(NA,0), tdist
=TRUE,data = Data)
```

```
## Warning: 101 rows with NAs omitted from model fitting.
```

```
##################### sig level 2 #######################
anova12_TPR <- anova(model_TPR_multilevel,model_TPR_multilevel_2)
anova12_TPR
```

```
##
##          df      AIC      BIC     AICc  logLik    LRT   pval       QE
## Full      3 -26.9774 -20.6943 -26.5488 16.4887               117.8564
## Reduced   2 -24.9796 -20.7909 -24.7691 14.4898 3.9978 0.0456 117.8564
```

```
###############sig level 3v #################
anova13_TPR <- anova(model_TPR_multilevel,model_TPR_multilevel_3)
anova13_TPR
```

```
##
##          df      AIC      BIC     AICc  logLik    LRT   pval       QE
## Full      3 -26.9774 -20.6943 -26.5488 16.4887               117.8564
## Reduced   2 -28.9774 -24.7887 -28.7668 16.4887 0.0000 1.0000 117.8564
```

7. Cardiovascular Challenge and Threat Index

7.1 Identyfing outliers

```
# CTI
model_fun_CTI <- rma(yi = CTI_cor_Z, vi= CTI_cor_Z_var, data = Data, slab = Ref_APA)
```

```
## Warning: 89 studies with NAs omitted from model fitting.
```

```
model_fun_CTI
```

```
##
## Random-Effects Model (k = 73; tau^2 estimator: REML)
##
## tau^2 (estimated amount of total heterogeneity): 0.0293 (SE = 0.0088)
## tau (square root of estimated tau^2 value):      0.1710
## I^2 (total heterogeneity / total variability):   66.76%
## H^2 (total variability / sampling variability):  3.01
##
## Test for Heterogeneity:
## Q(df = 72) = 195.5175, p-val < .0001
##
## Model Results:
##
## estimate      se    zval    pval   ci.lb   ci.ub
##   0.0966  0.0273  3.5456  0.0004  0.0432  0.1501  ***
##
## ---
## Signif. codes:  0 '***' 0.001 '**' 0.01 '*' 0.05 '.' 0.1 ' ' 1
```

```
# outliers diagnostics  - outlier rstudent > 3.0 #
Out_model_fun_CTI <- influence(model_fun_CTI)
Out_model_fun_CTI
```

```
##
##                                    rstudent  dffits cook.d  cov.r tau2.del
## Arthur et al., 2019.1               0.9248  0.0835 0.0070 1.0093   0.0293
## Arthur et al., 2019.2               1.4709  0.1301 0.0169 0.9987   0.0288
## Arthur et al., 2019.3              -0.5593 -0.0500 0.0025 1.0134   0.0295
## Behnke et al., 2024.1              -0.8577 -0.1373 0.0191 1.0322   0.0296
## Brimmell et al., 2019               2.3027  0.2735 0.0710 0.9574   0.0264
## Crowe et al., 2020.1               -0.0691 -0.0090 0.0001 1.0266   0.0299
## Crowe et al., 2020.2               -0.4474 -0.0523 0.0028 1.0241   0.0298
## Crowe et al., 2020.3               -0.7859 -0.0908 0.0083 1.0185   0.0295
## Crowe et al., 2020.4                0.1002  0.0105 0.0001 1.0265   0.0299
## Crowe et al., 2020.5               -0.6160 -0.0715 0.0051 1.0217   0.0297
## Dixon et al., 2019                  1.4682  0.1672 0.0276 0.9979   0.0285
## Hangen et al., 2019.1              -0.3652 -0.0563 0.0033 1.0428   0.0303
## Hangen et al., 2019.2              -0.3102 -0.0481 0.0024 1.0436   0.0303
## Hase et al., 2019.1                -0.7277 -0.0890 0.0080 1.0220   0.0296
## Hase et al., 2019.2                 0.1610  0.0183 0.0003 1.0288   0.0300
## Hase et al., 2019.3                -0.8196 -0.1001 0.0101 1.0199   0.0295
## Hase et al., 2019.4                -0.3606 -0.0444 0.0020 1.0274   0.0299
## Hase et al., 2019.5                -0.3961 -0.0516 0.0027 1.0304   0.0300
## Hase et al., 2019.6                -0.2932 -0.0386 0.0015 1.0315   0.0300
## Hase et al., in preparation.3      -0.8314 -0.0915 0.0084 1.0160   0.0295
## Hase et al., in preparation.4      -0.3439 -0.0382 0.0015 1.0225   0.0298
## Hase et al., in preparation.5      -0.0654 -0.0080 0.0001 1.0238   0.0299
## Hase et al., in preparation.6      -1.2063 -0.1317 0.0173 1.0069   0.0290
## Hase et al., in preparation.7       0.2567  0.0271 0.0007 1.0230   0.0298
## Hase et al., in preparation.8       0.0546  0.0050 0.0000 1.0238   0.0299
## Hase et al., in preparation.9       1.9726  0.2134 0.0444 0.9789   0.0276
## Hase et al., in preparation.10     -1.0078 -0.1073 0.0115 1.0115   0.0293
## Hase et al., in preparation.11      0.4944  0.0521 0.0027 1.0200   0.0297
## Hase et al., in preparation.12     -0.4151 -0.0450 0.0020 1.0210   0.0297
## Hase et al., in preparation.13     -0.4931 -0.0533 0.0029 1.0202   0.0297
## Hase et al., in preparation.14      0.3320  0.0346 0.0012 1.0216   0.0298
## Jewiss et al., 2023 (Study 1)       1.4638  0.1679 0.0278 0.9980   0.0285
## Jewiss et al., 2023 (Study 2).1     0.9973  0.1154 0.0133 1.0132   0.0292
## Jewiss et al., 2023 (Study 2).2    -0.1120 -0.0140 0.0002 1.0269   0.0299
## Jewiss et al., 2023 (Study 2).3    -0.2813 -0.0335 0.0011 1.0260   0.0299
## Jewiss et al., 2023 (Study 2).4     0.5813  0.0666 0.0045 1.0222   0.0297
## Jewiss et al., 2023 (Study 2).5     0.2298  0.0256 0.0007 1.0262   0.0299
## Jewiss et al., 2023 (Study 2).6     0.1008  0.0106 0.0001 1.0268   0.0299
## Jewiss et al., 2023 (Study 2).7    -0.1120 -0.0140 0.0002 1.0269   0.0299
## Jewiss et al., 2023 (Study 2).8    -0.3235 -0.0384 0.0015 1.0257   0.0299
## Jewiss et al., 2024                -0.4026 -0.0469 0.0022 1.0242   0.0298
## Khalaf et al., 2020.1               0.0747  0.0089 0.0001 1.0389   0.0302
## Khalaf et al., 2020.2               0.1620  0.0210 0.0005 1.0385   0.0302
## Miller et al., 2021.1               1.1413  0.1217 0.0148 1.0077   0.0291
## Miller et al., 2021.2               0.8737  0.0929 0.0086 1.0139   0.0294
## Miller et al., 2021.3               0.1721  0.0175 0.0003 1.0225   0.0298
## Miller et al., 2021.4               0.6184  0.0654 0.0043 1.0183   0.0296
## Miller et al., 2021.5              -0.0271 -0.0042 0.0000 1.0270   0.0299
## Miller et al., 2021.6              -0.9208 -0.1067 0.0114 1.0157   0.0294
## Miller et al., 2021.7              -0.3761 -0.0409 0.0017 1.0214   0.0298
## Miller et al., 2021.8               1.9509  0.2088 0.0426 0.9803   0.0277
## Miller et al., 2021.9              -0.3761 -0.0409 0.0017 1.0214   0.0298
## Miller et al., 2021.10             -0.2203 -0.0244 0.0006 1.0224   0.0298
```

```
## Moore et al., 2017               0.9692  0.1340 0.0180 1.0198   0.0293
## O'Brien et al., 2022            -0.5412 -0.0691 0.0048 1.0274   0.0298
## Petzel & Casad, 2022.1           5.8298  0.9133 0.4847 0.6332   0.0108
## Petzel & Casad, 2022.2          -1.2444 -0.1623 0.0261 1.0081   0.0288
## Sammy et al., 2017              -1.2714 -0.1573 0.0245 1.0063   0.0288
## Scheepers, 2017                  0.9215  0.1283 0.0165 1.0219   0.0294
## Slater et al., 2018             -0.5718 -0.0777 0.0061 1.0305   0.0299
## Smith et al., 2022.1            -0.7689 -0.0869 0.0076 1.0181   0.0295
## Smith et al., 2022.2            -1.5973 -0.1775 0.0310 0.9939   0.0283
## Smith et al., 2022.3            -2.3473 -0.2571 0.0632 0.9597   0.0266
## Trotman et al., 2018.2           1.0254  0.1368 0.0187 1.0164   0.0292
## Wood et al., 2018               -0.5571 -0.0629 0.0040 1.0213   0.0297
## Scheepers & Keller, 2022        -0.1555 -0.0240 0.0006 1.0418   0.0303
## Bosshard et al., 2023.2          0.0194  0.0012 0.0000 1.0435   0.0304
## Simms, 2022.1                    0.2447  0.0262 0.0007 1.0239   0.0298
## Simms, 2022.2                   -1.2819 -0.1410 0.0198 1.0048   0.0289
## Simms, 2022.3                   -0.4876 -0.0547 0.0030 1.0219   0.0297
## Simms, 2022.4                    0.8089  0.0892 0.0080 1.0163   0.0295
## Simms, 2022.5                   -0.6135 -0.0685 0.0047 1.0202   0.0297
## Simms, 2022.6                   -0.2691 -0.0307 0.0010 1.0239   0.0298
##                                  QE.del    hat weight    dfbs inf
## Arthur et al., 2019.1          193.6978 0.0081 0.8097  0.0835
## Arthur et al., 2019.2          191.6006 0.0077 0.7745  0.1304
## Arthur et al., 2019.3          195.3182 0.0077 0.7745 -0.0499
## Behnke et al., 2024.1          163.7671 0.0250 2.5040 -0.1376
## Brimmell et al., 2019          182.3373 0.0135 1.3534  0.2741
## Crowe et al., 2020.1           195.4559 0.0130 1.3027 -0.0090
## Crowe et al., 2020.2           195.4307 0.0130 1.3027 -0.0523
## Crowe et al., 2020.3           194.9129 0.0130 1.3027 -0.0907
## Crowe et al., 2020.4           195.2761 0.0130 1.3027  0.0105
## Crowe et al., 2020.5           195.2306 0.0130 1.3027 -0.0715
## Dixon et al., 2019             189.8045 0.0127 1.2664  0.1673
## Hangen et al., 2019.1          195.4956 0.0225 2.2456 -0.0566
## Hangen et al., 2019.2          195.5171 0.0225 2.2456 -0.0483
## Hase et al., 2019.1            194.9966 0.0146 1.4570 -0.0890
## Hase et al., 2019.2            195.1223 0.0143 1.4293  0.0183
## Hase et al., 2019.3            194.7758 0.0146 1.4570 -0.1001
## Hase et al., 2019.4            195.4910 0.0143 1.4293 -0.0444
## Hase et al., 2019.5            195.4728 0.0161 1.6080 -0.0517
## Hase et al., 2019.6            195.5158 0.0161 1.6080 -0.0386
## Hase et al., in preparation.3   194.8445 0.0119 1.1871 -0.0915
## Hase et al., in preparation.4   195.4930 0.0117 1.1657 -0.0382
## Hase et al., in preparation.5   195.4679 0.0117 1.1657 -0.0080
## Hase et al., in preparation.6   193.7563 0.0119 1.1871 -0.1317
## Hase et al., in preparation.7   195.0806 0.0117 1.1657  0.0270
## Hase et al., in preparation.8   195.3685 0.0117 1.1657  0.0050
## Hase et al., in preparation.9   186.9705 0.0114 1.1436  0.2141
## Hase et al., in preparation.10  194.4289 0.0112 1.1207 -0.1073
## Hase et al., in preparation.11  194.5921 0.0112 1.1207  0.0520
## Hase et al., in preparation.12  195.4525 0.0112 1.1207 -0.0450
## Hase et al., in preparation.13  195.3885 0.0112 1.1207 -0.0532
## Hase et al., in preparation.14  194.9626 0.0112 1.1207  0.0346
## Jewiss et al., 2023 (Study 1)   189.7397 0.0128 1.2848  0.1681
## Jewiss et al., 2023 (Study 2).1 192.3278 0.0132 1.3201  0.1154
## Jewiss et al., 2023 (Study 2).2 195.4813 0.0132 1.3201 -0.0140
## Jewiss et al., 2023 (Study 2).3 195.5145 0.0132 1.3201 -0.0335
```

```
## Jewiss et al., 2023 (Study 2).4 194.0983 0.0132 1.3201  0.0666
## Jewiss et al., 2023 (Study 2).5 195.0488 0.0132 1.3201  0.0256
## Jewiss et al., 2023 (Study 2).6 195.2694 0.0132 1.3201  0.0106
## Jewiss et al., 2023 (Study 2).7 195.4813 0.0132 1.3201 -0.0140
## Jewiss et al., 2023 (Study 2).8 195.5041 0.0132 1.3201 -0.0384
## Jewiss et al., 2024              195.4640 0.0128 1.2848 -0.0468
## Khalaf et al., 2020.1            194.9610 0.0191 1.9114  0.0089
## Khalaf et al., 2020.2            194.6647 0.0191 1.9114  0.0211
## Miller et al., 2021.1            192.2049 0.0112 1.1207  0.1217
## Miller et al., 2021.2            193.3667 0.0112 1.1207  0.0928
## Miller et al., 2021.3            195.2354 0.0112 1.1207  0.0175
## Miller et al., 2021.4            194.2466 0.0112 1.1207  0.0653
## Miller et al., 2021.5            195.4195 0.0132 1.3201 -0.0042
## Miller et al., 2021.6            194.5699 0.0132 1.3201 -0.1067
## Miller et al., 2021.7            195.4763 0.0112 1.1207 -0.0409
## Miller et al., 2021.8            187.2771 0.0112 1.1207  0.2095
## Miller et al., 2021.9            195.4763 0.0112 1.1207 -0.0409
## Miller et al., 2021.10           195.5174 0.0112 1.1207 -0.0243
## Moore et al., 2017               189.4170 0.0188 1.8779  0.1340
## O'Brien et al., 2022             195.3179 0.0157 1.5662 -0.0691
## Petzel & Casad, 2022.1           130.2561 0.0168 1.6817  0.8829   *
## Petzel & Casad, 2022.2           192.7979 0.0168 1.6817 -0.1621
## Sammy et al., 2017               193.0300 0.0152 1.5205 -0.1573
## Scheepers, 2017                  189.5262 0.0191 1.9114  0.1284
## Slater et al., 2018              195.2356 0.0178 1.7794 -0.0778
## Smith et al., 2022.1             194.9636 0.0125 1.2475 -0.0869
## Smith et al., 2022.2             191.9771 0.0125 1.2475 -0.1778
## Smith et al., 2022.3             187.2598 0.0125 1.2475 -0.2582
## Trotman et al., 2018.2           190.0914 0.0174 1.7445  0.1368
## Wood et al., 2018                195.3168 0.0123 1.2279 -0.0628
## Scheepers & Keller, 2022         195.3955 0.0207 2.0683 -0.0241
## Bosshard et al., 2023.2          194.8221 0.0213 2.1324  0.0012
## Simms, 2022.1                    195.0811 0.0121 1.2078  0.0262
## Simms, 2022.2                    193.4535 0.0121 1.2078 -0.1411
## Simms, 2022.3                    195.3939 0.0121 1.2078 -0.0547
## Simms, 2022.4                    193.4523 0.0121 1.2078  0.0891
## Simms, 2022.5                    195.2421 0.0121 1.2078 -0.0685
## Simms, 2022.6                    195.5150 0.0121 1.2078 -0.0307
```

```
ranktest(model_fun_CTI)
```

```
## Warning in cor.test.default(yi.star, vi, method = "kendall", exact = exact):
## nie można obliczyć dokładnej wartości prawdopodobieństwa z powtórzonymi
## wartościami
```

```
##
## Rank Correlation Test for Funnel Plot Asymmetry
##
## Kendall's tau = 0.0362, p = 0.6596
```

```
# leave one out analysis #

leave1out(model_fun_CTI)
```

```
##
##                                  estimate     se   zval   pval  ci.lb  ci.ub
## Arthur et al., 2019.1              0.0944 0.0274 3.4462 0.0006 0.0407 0.1480
## Arthur et al., 2019.2              0.0931 0.0272 3.4181 0.0006 0.0397 0.1465
## Arthur et al., 2019.3              0.0980 0.0274 3.5718 0.0004 0.0442 0.1518
## Behnke et al., 2024.1              0.1004 0.0277 3.6257 0.0003 0.0461 0.1547
## Brimmell et al., 2019              0.0894 0.0267 3.3513 0.0008 0.0371 0.1417
## Crowe et al., 2020.1               0.0969 0.0276 3.5083 0.0005 0.0428 0.1510
## Crowe et al., 2020.2               0.0981 0.0276 3.5556 0.0004 0.0440 0.1521
## Crowe et al., 2020.3               0.0991 0.0275 3.6033 0.0003 0.0452 0.1530
## Crowe et al., 2020.4               0.0964 0.0276 3.4891 0.0005 0.0422 0.1505
## Crowe et al., 2020.5               0.0986 0.0276 3.5788 0.0003 0.0446 0.1526
## Dixon et al., 2019                 0.0921 0.0272 3.3831 0.0007 0.0388 0.1455
## Hangen et al., 2019.1              0.0982 0.0278 3.5281 0.0004 0.0436 0.1528
## Hangen et al., 2019.2              0.0980 0.0278 3.5185 0.0004 0.0434 0.1525
## Hase et al., 2019.1                0.0991 0.0276 3.5956 0.0003 0.0451 0.1531
## Hase et al., 2019.2                0.0961 0.0276 3.4775 0.0005 0.0420 0.1503
## Hase et al., 2019.3                0.0994 0.0275 3.6102 0.0003 0.0454 0.1533
## Hase et al., 2019.4                0.0979 0.0276 3.5421 0.0004 0.0437 0.1520
## Hase et al., 2019.5                0.0981 0.0277 3.5442 0.0004 0.0438 0.1523
## Hase et al., 2019.6                0.0977 0.0277 3.5293 0.0004 0.0434 0.1520
## Hase et al., in preparation.3      0.0991 0.0275 3.6085 0.0003 0.0453 0.1530
## Hase et al., in preparation.4      0.0977 0.0276 3.5444 0.0004 0.0437 0.1517
## Hase et al., in preparation.5      0.0969 0.0276 3.5121 0.0004 0.0428 0.1509
## Hase et al., in preparation.6      0.1002 0.0274 3.6643 0.0002 0.0466 0.1538
## Hase et al., in preparation.7      0.0959 0.0276 3.4787 0.0005 0.0419 0.1499
## Hase et al., in preparation.8      0.0965 0.0276 3.4992 0.0005 0.0425 0.1506
## Hase et al., in preparation.9      0.0909 0.0270 3.3706 0.0007 0.0380 0.1438
## Hase et al., in preparation.10     0.0996 0.0274 3.6321 0.0003 0.0458 0.1533
## Hase et al., in preparation.11     0.0952 0.0275 3.4590 0.0005 0.0413 0.1492
## Hase et al., in preparation.12     0.0979 0.0275 3.5537 0.0004 0.0439 0.1519
## Hase et al., in preparation.13     0.0981 0.0275 3.5633 0.0004 0.0441 0.1521
## Hase et al., in preparation.14     0.0957 0.0275 3.4736 0.0005 0.0417 0.1497
## Jewiss et al., 2023 (Study 1)      0.0921 0.0272 3.3822 0.0007 0.0387 0.1455
## Jewiss et al., 2023 (Study 2).1    0.0935 0.0274 3.4078 0.0007 0.0397 0.1473
## Jewiss et al., 2023 (Study 2).2    0.0970 0.0276 3.5128 0.0004 0.0429 0.1512
## Jewiss et al., 2023 (Study 2).3    0.0976 0.0276 3.5337 0.0004 0.0434 0.1517
## Jewiss et al., 2023 (Study 2).4    0.0948 0.0276 3.4407 0.0006 0.0408 0.1488
## Jewiss et al., 2023 (Study 2).5    0.0959 0.0276 3.4746 0.0005 0.0418 0.1501
## Jewiss et al., 2023 (Study 2).6    0.0964 0.0276 3.4884 0.0005 0.0422 0.1505
## Jewiss et al., 2023 (Study 2).7    0.0970 0.0276 3.5128 0.0004 0.0429 0.1512
## Jewiss et al., 2023 (Study 2).8    0.0977 0.0276 3.5391 0.0004 0.0436 0.1518
## Jewiss et al., 2024                0.0979 0.0276 3.5500 0.0004 0.0439 0.1520
## Khalaf et al., 2020.1              0.0964 0.0278 3.4697 0.0005 0.0419 0.1509
## Khalaf et al., 2020.2              0.0961 0.0278 3.4584 0.0005 0.0416 0.1505
## Miller et al., 2021.1              0.0933 0.0274 3.4110 0.0006 0.0397 0.1470
## Miller et al., 2021.2              0.0941 0.0274 3.4289 0.0006 0.0403 0.1479
## Miller et al., 2021.3              0.0962 0.0276 3.4890 0.0005 0.0421 0.1502
## Miller et al., 2021.4              0.0949 0.0275 3.4486 0.0006 0.0409 0.1488
## Miller et al., 2021.5              0.0968 0.0276 3.5028 0.0005 0.0426 0.1509
## Miller et al., 2021.6              0.0996 0.0275 3.6241 0.0003 0.0457 0.1534
## Miller et al., 2021.7              0.0978 0.0275 3.5490 0.0004 0.0438 0.1518
## Miller et al., 2021.8              0.0910 0.0270 3.3728 0.0007 0.0381 0.1439
## Miller et al., 2021.9              0.0978 0.0275 3.5490 0.0004 0.0438 0.1518
## Miller et al., 2021.10             0.0973 0.0276 3.5308 0.0004 0.0433 0.1513
```

```
## Moore et al., 2017                0.0930 0.0275 3.3783 0.0007 0.0390 0.1469
## O'Brien et al., 2022              0.0985 0.0276 3.5666 0.0004 0.0444 0.1527
## Petzel & Casad, 2022.1            0.0777 0.0217 3.5807 0.0003 0.0352 0.1202
## Petzel & Casad, 2022.2            0.1010 0.0274 3.6922 0.0002 0.0474 0.1547
## Sammy et al., 2017                0.1009 0.0273 3.6905 0.0002 0.0473 0.1545
## Scheepers, 2017                   0.0931 0.0276 3.3802 0.0007 0.0391 0.1471
## Slater et al., 2018               0.0988 0.0277 3.5699 0.0004 0.0445 0.1530
## Smith et al., 2022.1              0.0990 0.0275 3.6003 0.0003 0.0451 0.1529
## Smith et al., 2022.2              0.1014 0.0272 3.7331 0.0002 0.0482 0.1547
## Smith et al., 2022.3              0.1035 0.0267 3.8759 0.0001 0.0512 0.1558
## Trotman et al., 2018.2            0.0929 0.0275 3.3813 0.0007 0.0391 0.1468
## Wood et al., 2018                 0.0984 0.0275 3.5709 0.0004 0.0444 0.1524
## Scheepers & Keller, 2022          0.0973 0.0278 3.4977 0.0005 0.0428 0.1518
## Bosshard et al., 2023.2           0.0966 0.0278 3.4697 0.0005 0.0420 0.1512
## Simms, 2022.1                     0.0959 0.0276 3.4780 0.0005 0.0419 0.1500
## Simms, 2022.2                     0.1005 0.0273 3.6773 0.0002 0.0469 0.1540
## Simms, 2022.3                     0.0981 0.0276 3.5619 0.0004 0.0441 0.1521
## Simms, 2022.4                     0.0942 0.0275 3.4284 0.0006 0.0404 0.1481
## Simms, 2022.5                     0.0985 0.0275 3.5785 0.0003 0.0446 0.1525
## Simms, 2022.6                     0.0975 0.0276 3.5345 0.0004 0.0434 0.1515
##                                         Q     Qp   tau2      I2     H2
## Arthur et al., 2019.1           193.6978 0.0000 0.0293 67.0244 3.0325
## Arthur et al., 2019.2           191.6006 0.0000 0.0288 66.6342 2.9971
## Arthur et al., 2019.3           195.3182 0.0000 0.0295 67.2019 3.0490
## Behnke et al., 2024.1           163.7671 0.0000 0.0296 59.1347 2.4471
## Brimmell et al., 2019           182.3373 0.0000 0.0264 64.5720 2.8226
## Crowe et al., 2020.1            195.4559 0.0000 0.0299 67.3830 3.0659
## Crowe et al., 2020.2            195.4307 0.0000 0.0298 67.2881 3.0570
## Crowe et al., 2020.3            194.9129 0.0000 0.0295 67.0806 3.0377
## Crowe et al., 2020.4            195.2761 0.0000 0.0299 67.3787 3.0655
## Crowe et al., 2020.5            195.2306 0.0000 0.0297 67.1992 3.0487
## Dixon et al., 2019              189.8045 0.0000 0.0285 66.3075 2.9680
## Hangen et al., 2019.1           195.4956 0.0000 0.0303 66.4471 2.9804
## Hangen et al., 2019.2           195.5171 0.0000 0.0303 66.4788 2.9832
## Hase et al., 2019.1             194.9966 0.0000 0.0296 67.0995 3.0395
## Hase et al., 2019.2             195.1223 0.0000 0.0300 67.3749 3.0651
## Hase et al., 2019.3             194.7758 0.0000 0.0295 67.0200 3.0321
## Hase et al., 2019.4             195.4910 0.0000 0.0299 67.3227 3.0602
## Hase et al., 2019.5             195.4728 0.0000 0.0300 67.2866 3.0569
## Hase et al., 2019.6             195.5158 0.0000 0.0300 67.3296 3.0609
## Hase et al., in preparation.3   194.8445 0.0000 0.0295 67.0619 3.0360
## Hase et al., in preparation.4   195.4930 0.0000 0.0298 67.3188 3.0599
## Hase et al., in preparation.5   195.4679 0.0000 0.0299 67.3677 3.0644
## Hase et al., in preparation.6   193.7563 0.0000 0.0290 66.7147 3.0043
## Hase et al., in preparation.7   195.0806 0.0000 0.0298 67.3367 3.0615
## Hase et al., in preparation.8   195.3685 0.0000 0.0299 67.3671 3.0644
## Hase et al., in preparation.9   186.9705 0.0000 0.0276 65.6274 2.9093
## Hase et al., in preparation.10  194.4289 0.0000 0.0293 66.9308 3.0240
## Hase et al., in preparation.11  194.5921 0.0000 0.0297 67.2513 3.0536
## Hase et al., in preparation.12  195.4525 0.0000 0.0297 67.2912 3.0573
## Hase et al., in preparation.13  195.3885 0.0000 0.0297 67.2612 3.0545
## Hase et al., in preparation.14  194.9626 0.0000 0.0298 67.3112 3.0592
## Jewiss et al., 2023 (Study 1)   189.7397 0.0000 0.0285 66.2999 2.9674
## Jewiss et al., 2023 (Study 2).1 192.3278 0.0000 0.0292 66.8663 3.0181
## Jewiss et al., 2023 (Study 2).2 195.4813 0.0000 0.0299 67.3808 3.0657
## Jewiss et al., 2023 (Study 2).3 195.5145 0.0000 0.0299 67.3485 3.0626
```

```
## Jewiss et al., 2023 (Study 2).4 194.0983 0.0000 0.0297 67.2072 3.0494
## Jewiss et al., 2023 (Study 2).5 195.0488 0.0000 0.0299 67.3568 3.0634
## Jewiss et al., 2023 (Study 2).6 195.2694 0.0000 0.0299 67.3799 3.0656
## Jewiss et al., 2023 (Study 2).7 195.4813 0.0000 0.0299 67.3808 3.0657
## Jewiss et al., 2023 (Study 2).8 195.5041 0.0000 0.0299 67.3358 3.0615
## Jewiss et al., 2024            195.4640 0.0000 0.0298 67.3064 3.0587
## Khalaf et al., 2020.1          194.9610 0.0000 0.0302 67.2527 3.0537
## Khalaf et al., 2020.2          194.6647 0.0000 0.0302 67.2364 3.0522
## Miller et al., 2021.1          192.2049 0.0000 0.0291 66.7858 3.0108
## Miller et al., 2021.2          193.3667 0.0000 0.0294 67.0223 3.0324
## Miller et al., 2021.3          195.2354 0.0000 0.0298 67.3478 3.0626
## Miller et al., 2021.4          194.2466 0.0000 0.0296 67.1901 3.0479
## Miller et al., 2021.5          195.4195 0.0000 0.0299 67.3860 3.0662
## Miller et al., 2021.6          194.5699 0.0000 0.0294 66.9614 3.0268
## Miller et al., 2021.7          195.4763 0.0000 0.0298 67.3043 3.0585
## Miller et al., 2021.8          187.2771 0.0000 0.0277 65.6962 2.9151
## Miller et al., 2021.9          195.4763 0.0000 0.0298 67.3043 3.0585
## Miller et al., 2021.10         195.5174 0.0000 0.0298 67.3432 3.0621
## Moore et al., 2017             189.4170 0.0000 0.0293 66.5833 2.9925
## O'Brien et al., 2022           195.3179 0.0000 0.0298 67.2132 3.0500
## Petzel & Casad, 2022.1         130.2561 0.0000 0.0108 42.4154 1.7366
## Petzel & Casad, 2022.2         192.7979 0.0000 0.0288 66.3660 2.9732
## Sammy et al., 2017             193.0300 0.0000 0.0288 66.4475 2.9804
## Scheepers, 2017                189.5262 0.0000 0.0294 66.6147 2.9953
## Slater et al., 2018            195.2356 0.0000 0.0299 67.1155 3.0409
## Smith et al., 2022.1           194.9636 0.0000 0.0295 67.1010 3.0396
## Smith et al., 2022.2           191.9771 0.0000 0.0283 66.1631 2.9554
## Smith et al., 2022.3           187.2598 0.0000 0.0266 64.7484 2.8368
## Trotman et al., 2018.2         190.0914 0.0000 0.0292 66.6214 2.9959
## Wood et al., 2018              195.3168 0.0000 0.0297 67.2348 3.0520
## Scheepers & Keller, 2022       195.3955 0.0000 0.0303 67.0576 3.0356
## Bosshard et al., 2023.2        194.8221 0.0000 0.0304 66.9462 3.0254
## Simms, 2022.1                  195.0811 0.0000 0.0298 67.3442 3.0622
## Simms, 2022.2                  193.4535 0.0000 0.0289 66.6186 2.9957
## Simms, 2022.3                  195.3939 0.0000 0.0297 67.2680 3.0551
## Simms, 2022.4                  193.4523 0.0000 0.0295 67.0602 3.0358
## Simms, 2022.5                  195.2421 0.0000 0.0297 67.2044 3.0492
## Simms, 2022.6                  195.5150 0.0000 0.0298 67.3434 3.0622
```

```
# Egger#
model_fun_CTI_OutEgger <- rma.mv(yi = CTI_cor_Z, V = CTI_cor_Z_var, random = ~ 1 | pape
r_id/effect_size_id, mod = ~sqrt(CTI_cor_Z_var), tdist=TRUE, data = Data)
```

```
## Warning: 89 rows with NAs omitted from model fitting.
```

```
model_fun_CTI_OutEgger
```

```
##
## Multivariate Meta-Analysis Model (k = 73; method: REML)
##
## Variance Components:
##
##             estim    sqrt  nlvls  fixed                   factor
## sigma^2.1  0.0044  0.0664     28     no                 paper_id
## sigma^2.2  0.0266  0.1630     73     no  paper_id/effect_size_id
##
## Test for Residual Heterogeneity:
## QE(df = 71) = 171.9220, p-val < .0001
##
## Test of Moderators (coefficient 2):
## F(df1 = 1, df2 = 71) = 0.2902, p-val = 0.5918
##
## Model Results:
##
##                      estimate      se    tval  df    pval    ci.lb   ci.ub
## intrcpt                0.0501  0.1003  0.5001  71  0.6185  -0.1498  0.2501
## sqrt(CTI_cor_Z_var)    0.3488  0.6474  0.5387  71  0.5918  -0.9421  1.6397
##
## ---
## Signif. codes:  0 '***' 0.001 '**' 0.01 '*' 0.05 '.' 0.1 ' ' 1
```

```
# Run trim-and-fill analysis for the right side
model_fun_CTI_tf_right <- trimfill(model_fun_CTI, side = "right")

# Run trim-and-fill analysis for the left side
model_fun_CTI_tf_left <- trimfill(model_fun_CTI, side = "left")

# Print the trim-and-fill model results
print(model_fun_CTI_tf_right)
```

```
##
## Estimated number of missing studies on the right side: 13 (SE = 5.6241)
##
## Random-Effects Model (k = 86; tau^2 estimator: REML)
##
## tau^2 (estimated amount of total heterogeneity): 0.0393 (SE = 0.0098)
## tau (square root of estimated tau^2 value):      0.1983
## I^2 (total heterogeneity / total variability):   71.71%
## H^2 (total variability / sampling variability):  3.54
##
## Test for Heterogeneity:
## Q(df = 85) = 280.9697, p-val < .0001
##
## Model Results:
##
## estimate      se    zval    pval   ci.lb   ci.ub
##   0.1508  0.0276  5.4701  <.0001  0.0968  0.2048  ***
##
## ---
## Signif. codes:  0 '***' 0.001 '**' 0.01 '*' 0.05 '.' 0.1 ' ' 1
```

```
print(model_fun_CTI_tf_left)
```

```
##
## Estimated number of missing studies on the left side: 0 (SE = 4.4604)
##
## Random-Effects Model (k = 73; tau^2 estimator: REML)
##
## tau^2 (estimated amount of total heterogeneity): 0.0293 (SE = 0.0088)
## tau (square root of estimated tau^2 value):      0.1710
## I^2 (total heterogeneity / total variability):   66.76%
## H^2 (total variability / sampling variability):  3.01
##
## Test for Heterogeneity:
## Q(df = 72) = 195.5175, p-val < .0001
##
## Model Results:
##
## estimate      se    zval    pval   ci.lb   ci.ub
##   0.0966  0.0273  3.5456  0.0004  0.0432  0.1501  ***
##
## ---
## Signif. codes:  0 '***' 0.001 '**' 0.01 '*' 0.05 '.' 0.1 ' ' 1
```

```
# Combine the number of studies imputed from both sides
total_imputed_studies <- model_fun_CTI_tf_right$k0 + model_fun_CTI_tf_left$k0
cat("Total number of imputed studies (both sides) for CTI:", total_imputed_studies,
"\n")
```

```
## Total number of imputed studies (both sides) for CTI: 13
```

```
# Generate funnel plots only right
#par(mfrow=c(1, 3))
#funnel(model_fun_CTI, main="Original Model")
funnel(model_fun_CTI_tf_right, main="Trim-and-Fill Right", xlab = 'CTI')
```

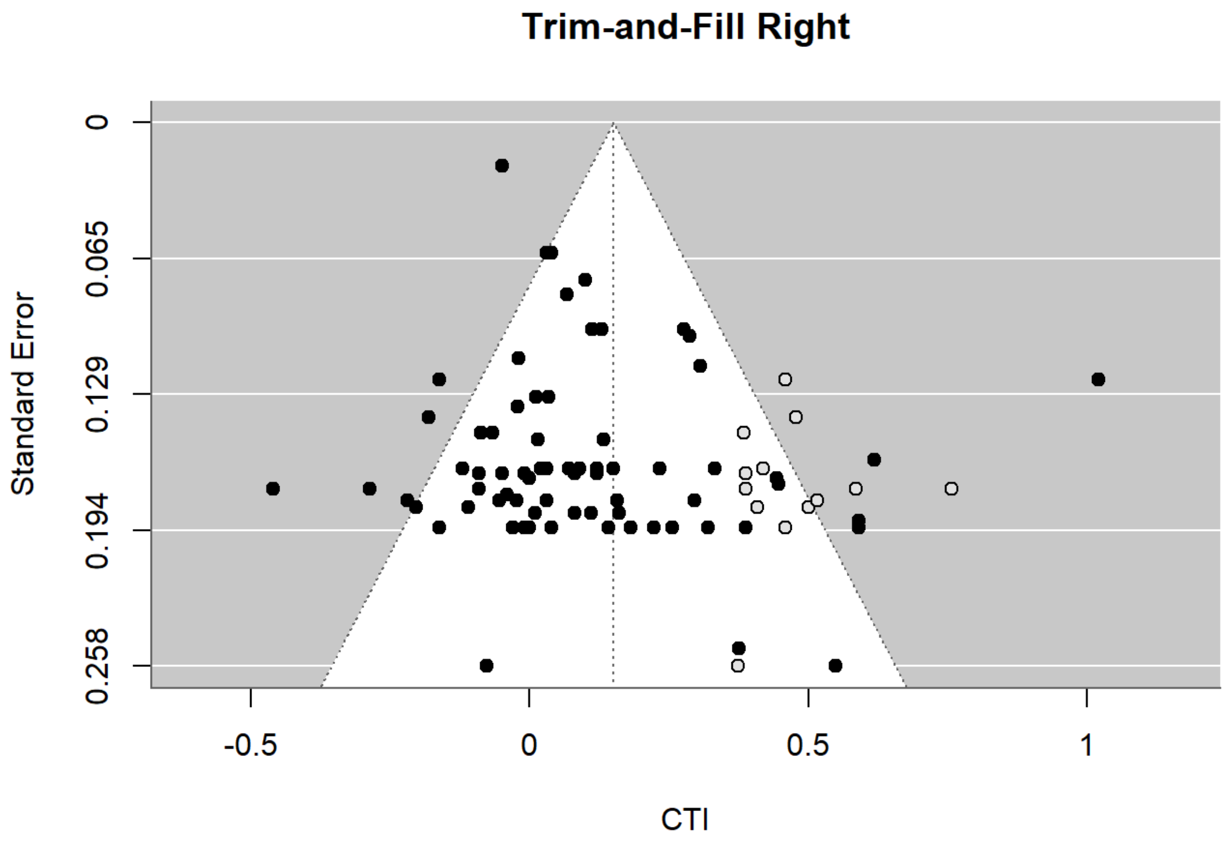


```
#funnel(model_fun_CTI_tf_left, main="Trim-and-Fill Left")

# Return to single plot layout
#par(mfrow=c(1, 1))
```

7.2 Multilevel Model

```
model_CTI_multilevel <- rma.mv(yi = CTI_cor_Z, V = CTI_cor_Z_var, slab = Ref_APA, data
= Data, random = ~ 1 | paper_id/effect_size_id, test = "t", method = "REML")
```

```
## Warning: 89 rows with NAs omitted from model fitting.
```

```
model_CTI_multilevel
```

```
##
## Multivariate Meta-Analysis Model (k = 73; method: REML)
##
## Variance Components:
##
##             estim    sqrt  nlvls  fixed                   factor
## sigma^2.1  0.0000  0.0000     28     no                 paper_id
## sigma^2.2  0.0293  0.1710     73     no  paper_id/effect_size_id
##
## Test for Heterogeneity:
## Q(df = 72) = 195.5175, p-val < .0001
##
## Model Results:
##
## estimate      se    tval  df    pval   ci.lb   ci.ub
##   0.0966  0.0273  3.5456  72  0.0007  0.0423  0.1510  ***
##
## ---
## Signif. codes:  0 '***' 0.001 '**' 0.01 '*' 0.05 '.' 0.1 ' ' 1
```

```
convert_z2r(0.1150)
```

```
## [1] 0.1144957
```

```
predict_CTI <-predict(model_CTI_multilevel, digits=3, transf=transf.ztor, level = 95)
predict_CTI
```

```
##
##   pred ci.lb ci.ub  pi.lb pi.ub
##  0.096 0.042 0.150 -0.244 0.415
```

```
forest.rma(model_CTI_multilevel, header = "CTI",slab = Data$Ref_APA, alim=c(-1,1.6))
```

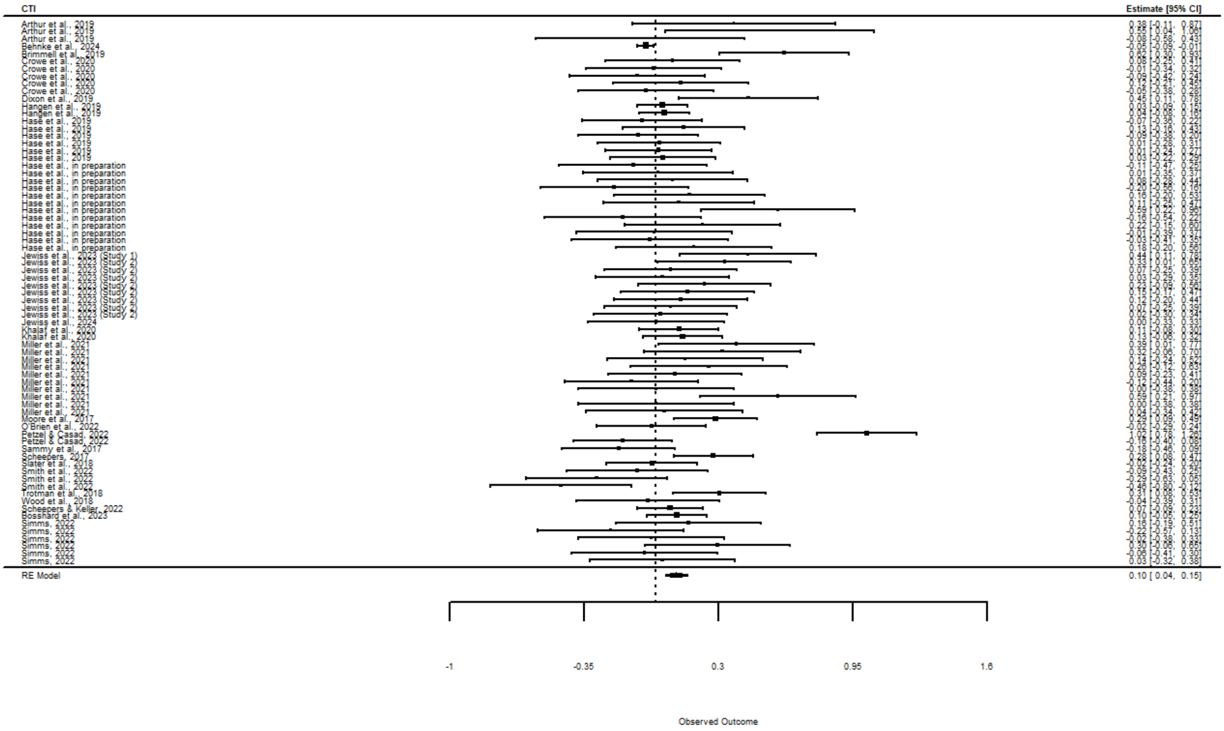


```
######## list 1 ########
list_CTI <- Data$CTI_cor_Z_var

############ sum 1######################
sum_CTI <- sum(list_CTI, na.rm = TRUE)

###################### sum 2 #####################
sum2_CTI <- (sum_CTI)^2

####################### list 2 ##############
list_In_CTI <- Data$CTI_cor_Z_var_Sq

#################### sum 3 ######################
sum_In_CTI<- sum(list_In_CTI, na.rm = TRUE)

############### numerator ##############
numerator_CTI<- (model_CTI_multilevel$k-1)*sum_CTI

############# denominator #############
denominator_CTI<- sum2_CTI - sum_In_CTI

############## eps ################
EPS_CTI<- numerator_CTI / denominator_CTI

############### i2 1 level ##################
I2_1_CTI <- (EPS_CTI) / (model_CTI_multilevel$sigma2[1] + model_CTI_multilevel$sigma2
[2] + EPS_CTI) *100
I2_1_CTI
```

```
## [1] 99.91892
```

```
############## i2 2 level #################
I2_2_CTI <- (model_CTI_multilevel$sigma2[1]) / (model_CTI_multilevel$sigma2[1] + model_
CTI_multilevel$sigma2[2] + EPS_CTI) *100
I2_2_CTI
```

```
## [1] 9.976715e-10
```

```
########### I2 level 3
I2_3_CTI <- (model_CTI_multilevel$sigma2[2]) / (model_CTI_multilevel$sigma2[1] + model_
CTI_multilevel$sigma2[2] + EPS_CTI) *100
I2_3_CTI
```

```
## [1] 0.08107815
```

```
############### ML without level 2 ##########
model_CTI_multilevel_2 <- rma.mv(yi = CTI_cor_Z, V = CTI_cor_Z_var, slab = Ref_APA, ran
dom = ~ 1 | paper_id/effect_size_id, test = "t", method = "REML", sigma2=c(0,NA), tdist
=TRUE,data = Data)
```

```
## Warning: 89 rows with NAs omitted from model fitting.
```

```
############# ml without level 3 ###########
model_CTI_multilevel_3 <- rma.mv(yi = CTI_cor_Z, V = CTI_cor_Z_var, slab = Ref_APA, ran
dom = ~ 1 | paper_id/effect_size_id, test = "t", method = "REML", sigma2=c(NA,0), tdist
=TRUE,data = Data)
```

```
## Warning: 89 rows with NAs omitted from model fitting.
```

```
##################### sig level 2 #######################
anova12_CTI <- anova(model_CTI_multilevel,model_CTI_multilevel_2)
anova12_CTI
```

```
##
##         df     AIC    BIC    AICc logLik    LRT   pval       QE
## Full     3  0.9258 7.7558  1.2787 2.5371                195.5175
## Reduced  2 -1.0742 3.4791 -0.9003 2.5371 0.0000 1.0000 195.5175
```

```
###############sig level 3v #################
anova13_CTI <- anova(model_CTI_multilevel,model_CTI_multilevel_3)
anova13_CTI
```

```
##
##         df     AIC     BIC    AICc  logLik     LRT   pval       QE
## Full     3  0.9258  7.7558  1.2787  2.5371                 195.5175
## Reduced  2 17.3488 21.9021 17.5227 -6.6744 18.4230 <.0001 195.5175
```

8. Cognitive Challenge and Threat Index

8.1 Identyfing outliers

```
# Cogni
model_fun_Cogni <- rma(yi = Cogni_cor_Z, vi= Cogni_cor_Z_var, data = Data, slab = Ref_A
PA)
```

```
## Warning: 41 studies with NAs omitted from model fitting.
```

```
model_fun_Cogni
```

```
##
## Random-Effects Model (k = 121; tau^2 estimator: REML)
##
## tau^2 (estimated amount of total heterogeneity): 0.0209 (SE = 0.0046)
## tau (square root of estimated tau^2 value):      0.1445
## I^2 (total heterogeneity / total variability):   72.22%
## H^2 (total variability / sampling variability):  3.60
##
## Test for Heterogeneity:
## Q(df = 120) = 377.1303, p-val < .0001
##
## Model Results:
##
## estimate      se    zval    pval   ci.lb   ci.ub
##   0.1426  0.0176  8.0869  <.0001  0.1080  0.1771  ***
##
## ---
## Signif. codes:  0 '***' 0.001 '**' 0.01 '*' 0.05 '.' 0.1 ' ' 1
```

```
# outliers diagnostics  - outlier rstudent > 3.0 #
Out_model_fun_Cogni <- influence(model_fun_Cogni)
Out_model_fun_Cogni
```

```
## 
##                                      rstudient  dffits cook.d  cov.r tau2.del 
## Baumgartner & Schneider, 2023           0.6951  0.0742 0.0055 1.0158   0.0211 
## Behnke et al., 2020.1                   1.0369  0.1010 0.0102 1.0102   0.0209 
## Behnke et al., 2020.2                   0.2726  0.0338 0.0012 1.0180   0.0212 
## Behnke et al., 2022                     0.5509  0.0750 0.0057 1.0249   0.0212 
## Behnke et al., 2024.1                  -0.4259 -0.0425 0.0018 1.0253   0.0212 
## Brimmell et al., 2019                   1.0899  0.0896 0.0080 1.0069   0.0209 
## Cabral et al., 2024.1                  -0.0635  0.0014 0.0000 1.0179   0.0212 
## Cabral et al., 2024.2                   0.0474  0.0122 0.0001 1.0181   0.0212 
## Cabral et al., 2024.3                  -0.1187 -0.0041 0.0000 1.0178   0.0212 
## Carenzo et al., 2020.1                 -2.9849 -0.4105 0.1512 0.9269   0.0181 
## Carenzo et al., 2020.2                  2.9348  0.2503 0.0571 0.9391   0.0185 
## Gurera & Isaacowitz, 2022.1             0.0629  0.0133 0.0002 1.0175   0.0212 
## Gurera & Isaacowitz, 2022.2             0.9287  0.0899 0.0081 1.0114   0.0210 
## Gurera & Isaacowitz, 2022.3             0.1302  0.0170 0.0003 1.0141   0.0211 
## Gurera & Isaacowitz, 2022.4            -0.6745 -0.0547 0.0030 1.0100   0.0210 
## Hase et al., 2019.1                    -0.2894 -0.0196 0.0004 1.0134   0.0211 
## Hase et al., 2019.2                     0.3804  0.0378 0.0014 1.0135   0.0211 
## Hase et al., 2019.3                    -2.1304 -0.2053 0.0413 0.9809   0.0200 
## Hase et al., 2019.4                    -1.2910 -0.1146 0.0131 1.0010   0.0207 
## Hase et al., 2019.5                     1.1090  0.1007 0.0101 1.0081   0.0209 
## Hase et al., 2019.6                     1.0580  0.0968 0.0094 1.0089   0.0209 
## Hase et al., in preparation.1          -1.9693 -0.1839 0.0333 0.9859   0.0202 
## Hase et al., in preparation.2          -0.4694 -0.0355 0.0013 1.0117   0.0210 
## Hase et al., in preparation.3          -0.0941 -0.0025 0.0000 1.0111   0.0211 
## Hase et al., in preparation.4           0.7580  0.0598 0.0036 1.0088   0.0210 
## Hase et al., in preparation.5           1.0974  0.0826 0.0068 1.0059   0.0209 
## Hase et al., in preparation.6          -1.4914 -0.1202 0.0144 0.9975   0.0206 
## Hase et al., in preparation.7          -0.7349 -0.0538 0.0029 1.0073   0.0209 
## Hase et al., in preparation.8          -1.2097 -0.0941 0.0088 1.0018   0.0208 
## Hase et al., in preparation.9          -0.7691 -0.0561 0.0031 1.0068   0.0209 
## Hase et al., in preparation.10          0.6042  0.0479 0.0023 1.0094   0.0210 
## Hase et al., in preparation.11         -0.6767 -0.0480 0.0023 1.0074   0.0210 
## Hase et al., in preparation.12         -0.1753 -0.0087 0.0001 1.0102   0.0210 
## Hase et al., in preparation.13         -0.0064  0.0040 0.0000 1.0105   0.0211 
## Hase et al., in preparation.14         -0.3430 -0.0216 0.0005 1.0096   0.0210 
## Jamieson et al., 2022.1                 1.7000  0.1788 0.0311 0.9930   0.0202 
## Jamieson et al., 2022.2                 2.2201  0.2132 0.0430 0.9717   0.0195 
## Jamieson et al., 2022.3                 1.7107  0.1669 0.0273 0.9939   0.0203 
## Journault et al., in preparation.1      1.6881  0.1767 0.0304 0.9936   0.0202 
## Journault et al., in preparation.2      0.4541  0.0605 0.0037 1.0234   0.0212 
## Journault et al., in preparation.3      1.0794  0.1228 0.0151 1.0128   0.0209 
## Khalaf et al., 2020.1                   1.2190  0.1213 0.0147 1.0075   0.0208 
## Khalaf et al., 2020.2                   1.0883  0.1103 0.0122 1.0102   0.0209 
## Laurin & Pellet, 2023                  -0.1477 -0.0065 0.0000 1.0277   0.0213 
## Lee et al., 2019                       -0.6117 -0.0655 0.0043 1.0207   0.0211 
## Malkoc et al., 2023                    -0.5209 -0.0533 0.0029 1.0218   0.0212 
## Malkoc et al., 2024                    -0.2157 -0.0149 0.0002 1.0253   0.0213 
## Moe & Putwain, 2020.1                   1.1301  0.1016 0.0103 1.0076   0.0209 
## Moe & Putwain, 2020.2                   0.4469  0.0463 0.0022 1.0150   0.0211 
## Moe & Putwain, 2020.3                   0.5825  0.0578 0.0034 1.0141   0.0211 
## Moe & Putwain, 2020.4                   0.4093  0.0431 0.0019 1.0152   0.0211 
## Moe & Putwain, 2020.5                   0.5226  0.0527 0.0028 1.0145   0.0211 
## Moe & Putwain, 2020.6                   0.9793  0.0899 0.0081 1.0098   0.0209 
```

```
## Moe & Putwain, 2020.7              0.5118  0.0518 0.0027 1.0146   0.0211
## Moore et al., 2017                 1.1401  0.1134 0.0128 1.0090   0.0209
## Mosley et al., 2018.1             -0.9855 -0.0854 0.0073 1.0063   0.0209
## Mosley et al., 2018.2             -0.4972 -0.0387 0.0015 1.0120   0.0210
## Mulvenna et al., 2023.1           -0.4669 -0.0457 0.0021 1.0219   0.0212
## Mulvenna et al., 2023.2           -0.2711 -0.0215 0.0005 1.0241   0.0213
## O'Brien et al., 2022              -1.6605 -0.1619 0.0259 0.9927   0.0204
## Sammy et al., 2017                 1.6633  0.1368 0.0186 0.9973   0.0205
## Schickel et al., 2023.1           -0.0682  0.0016 0.0000 1.0208   0.0212
## Schickel et al., 2023.2            0.6618  0.0744 0.0056 1.0175   0.0211
## Sharpe et al., 2024.1              0.2546  0.0241 0.0006 1.0112   0.0211
## Sharpe et al., 2024.2             -1.3253 -0.1245 0.0154 1.0005   0.0206
## Sharpe et al., 2024.3              1.9858  0.1311 0.0171 0.9934   0.0205
## Thornton et al., 2020.1           -0.3631 -0.0304 0.0009 1.0189   0.0212
## Thornton et al., 2020.2           -0.5396 -0.0503 0.0025 1.0170   0.0211
## Trotman et al., 2018.1             0.5538  0.0589 0.0035 1.0160   0.0211
## Trotman et al., 2018.2            -0.2277 -0.0150 0.0002 1.0171   0.0212
## Turner et al., 2021.1              0.1059  0.0185 0.0003 1.0191   0.0212
## Turner et al., 2021.2              0.9351  0.0946 0.0090 1.0123   0.0210
## Turner et al., 2021.3             -0.1219 -0.0043 0.0000 1.0187   0.0212
## Wood et al., 2018                  1.1312  0.0874 0.0076 1.0058   0.0209
## Scheepers & Keller, 2022           0.6364  0.0743 0.0056 1.0188   0.0211
## Bosshard et al., 2023.1            0.3778  0.0510 0.0026 1.0230   0.0212
## Mansell, 2023.1                   -1.1740 -0.1264 0.0159 1.0050   0.0207
## Mansell, 2023.2                   -1.3615 -0.1503 0.0223 1.0000   0.0205
## Jamieson et al., 2021.1            1.4866  0.1630 0.0261 1.0008   0.0205
## Jamieson et al., 2021.2            0.9583  0.1139 0.0130 1.0162   0.0210
## Sharpe et al., 2024.4             -1.4100 -0.0962 0.0092 0.9990   0.0207
## Sharpe et al., 2024.5             -0.9652 -0.0634 0.0040 1.0036   0.0209
## Sharpe et al., 2024.6             -0.3155 -0.0177 0.0003 1.0076   0.0210
## Sharpe et al., 2024.7             -0.5930 -0.0369 0.0014 1.0063   0.0210
## Sharpe et al., 2024.8             -1.3708 -0.0933 0.0087 0.9994   0.0207
## Sharpe et al., 2024.9             -1.7281 -0.1204 0.0144 0.9947   0.0206
## Sharpe et al., 2024.10            -0.5745 -0.0356 0.0013 1.0064   0.0210
## Sharpe et al., 2024.11            -0.6189 -0.0387 0.0015 1.0061   0.0210
## Sharpe et al., 2024.12            -0.4763 -0.0198 0.0004 1.0030   0.0209
## Sharpe et al., 2024.13            -0.7951 -0.0345 0.0012 1.0021   0.0209
## Sharpe et al., 2024.14             0.1206  0.0069 0.0000 1.0038   0.0210
## Sharpe et al., 2024.15            -0.2139 -0.0079 0.0001 1.0035   0.0209
## Simms, 2022.1                      0.1358  0.0154 0.0002 1.0116   0.0211
## Simms, 2022.2                      0.3480  0.0314 0.0010 1.0112   0.0211
## Simms, 2022.3                     -0.3100 -0.0197 0.0004 1.0106   0.0210
## Simms, 2022.4                     -0.4627 -0.0322 0.0010 1.0097   0.0210
## Simms, 2022.5                     -0.0156  0.0037 0.0000 1.0115   0.0211
## Simms, 2022.6                     -1.0707 -0.0838 0.0070 1.0038   0.0208
## Marr et al., 2021                  0.4656  0.0586 0.0035 1.0213   0.0212
## Conlon et al., 2022.1              1.4311  0.1269 0.0160 1.0022   0.0207
## Conlon et al., 2022.2              0.8707  0.0835 0.0070 1.0118   0.0210
## van Gog et al., 2024.1            -0.5458 -0.0470 0.0022 1.0140   0.0211
## van Gog et al., 2024.2            -0.5993 -0.0525 0.0028 1.0134   0.0210
## van Gog et al., 2024.3            -0.9314 -0.0840 0.0071 1.0078   0.0209
## van Gog et al., 2024.4            -0.6238 -0.0528 0.0028 1.0119   0.0210
## van Gog et al., 2024.5            -0.3600 -0.0266 0.0007 1.0137   0.0211
## van Gog et al., 2024.6             0.1454  0.0192 0.0004 1.0152   0.0211
## van Gog et al., 2024.7            -0.4333 -0.0351 0.0012 1.0147   0.0211
## van Gog et al., 2024.8            -1.1237 -0.1077 0.0116 1.0049   0.0208
```

```
## van Gog et al., 2024.9             -1.6375 -0.1571 0.0244 0.9934   0.0204
## van Gog et al., 2024.10             0.4059  0.0417 0.0018 1.0146   0.0211
## van Gog et al., 2024.11            -0.4485 -0.0341 0.0012 1.0123   0.0211
## van Gog et al., 2024.12            -0.3509 -0.0252 0.0006 1.0130   0.0211
## van Gog et al., 2024.13            -0.8418 -0.0759 0.0058 1.0095   0.0209
## van Gog et al., 2024.14            -0.5817 -0.0493 0.0024 1.0128   0.0210
## van Gog et al., 2024.15            -1.2941 -0.1275 0.0161 1.0014   0.0206
## van Gog et al., 2024.16            -0.0618  0.0013 0.0000 1.0170   0.0212
## van Gog et al., 2024.17            -0.7555 -0.0685 0.0047 1.0112   0.0210
## van Gog et al., 2024.18            -1.6826 -0.1726 0.0293 0.9915   0.0203
## van Gog et al., 2024.19            -0.6509 -0.0577 0.0033 1.0127   0.0210
## van Gog et al., 2024.20             0.1551  0.0218 0.0005 1.0172   0.0212
##                                     QE.del    hat weight    dfbs inf
## Baumgartner & Schneider, 2023      376.0458 0.0098 0.9785  0.0742
## Behnke et al., 2020.1              374.7321 0.0093 0.9263  0.1010
## Behnke et al., 2020.2              377.0359 0.0093 0.9263  0.0338
## Behnke et al., 2022                371.4928 0.0143 1.4307  0.0752
## Behnke et al., 2024.1              360.0503 0.0146 1.4585 -0.0426
## Brimmell et al., 2019              375.2546 0.0067 0.6678  0.0896
## Cabral et al., 2024.1              377.0734 0.0092 0.9173  0.0014
## Cabral et al., 2024.2              377.1268 0.0092 0.9173  0.0122
## Cabral et al., 2024.3              377.0228 0.0092 0.9173 -0.0041
## Carenzo et al., 2020.1             341.0679 0.0114 1.1383 -0.4063   *
## Carenzo et al., 2020.2             345.7446 0.0114 1.1383  0.2481
## Gurera & Isaacowitz, 2022.1        377.1294 0.0088 0.8834  0.0133
## Gurera & Isaacowitz, 2022.2        375.3689 0.0088 0.8834  0.0899
## Gurera & Isaacowitz, 2022.3        377.1242 0.0071 0.7125  0.0170
## Gurera & Isaacowitz, 2022.4        376.0585 0.0071 0.7125 -0.0547
## Hase et al., 2019.1                376.8703 0.0073 0.7290 -0.0196
## Hase et al., 2019.2                376.9498 0.0071 0.7125  0.0377
## Hase et al., 2019.3                367.8462 0.0073 0.7290 -0.2059
## Hase et al., 2019.4                373.5908 0.0071 0.7125 -0.1147
## Hase et al., 2019.5                374.7727 0.0082 0.8213  0.1007
## Hase et al., 2019.6                374.9992 0.0082 0.8213  0.0968
## Hase et al., in preparation.1      369.3800 0.0070 0.7039 -0.1844
## Hase et al., in preparation.2      376.5717 0.0070 0.7039 -0.0355
## Hase et al., in preparation.3      377.0886 0.0057 0.5731 -0.0025
## Hase et al., in preparation.4      376.3613 0.0056 0.5612  0.0598
## Hase et al., in preparation.5      375.4259 0.0056 0.5612  0.0826
## Hase et al., in preparation.6      373.2222 0.0057 0.5731 -0.1203
## Hase et al., in preparation.7      376.1049 0.0056 0.5612 -0.0538
## Hase et al., in preparation.8      374.5367 0.0056 0.5612 -0.0942
## Hase et al., in preparation.9      376.0320 0.0055 0.5490 -0.0560
## Hase et al., in preparation.10     376.6744 0.0054 0.5364  0.0479
## Hase et al., in preparation.11     376.2748 0.0054 0.5364 -0.0479
## Hase et al., in preparation.12     377.0408 0.0054 0.5364 -0.0087
## Hase et al., in preparation.13     377.1226 0.0054 0.5364  0.0040
## Hase et al., in preparation.14     376.8717 0.0054 0.5364 -0.0216
## Jamieson et al., 2022.1            356.9870 0.0130 1.3021  0.1780
## Jamieson et al., 2022.2            352.6334 0.0123 1.2276  0.2118
## Jamieson et al., 2022.3            366.8627 0.0112 1.1165  0.1666
## Journault et al., in preparation.1 358.8824 0.0129 1.2858  0.1760
## Journault et al., in preparation.2 376.2039 0.0128 1.2811  0.0606
## Journault et al., in preparation.3 370.1425 0.0128 1.2817  0.1228
## Khalaf et al., 2020.1              373.0985 0.0102 1.0187  0.1212
## Khalaf et al., 2020.2              373.9681 0.0102 1.0187  0.1103
```

```
## Laurin & Pellet, 2023            374.9994 0.0144 1.4383 -0.0065
## Lee et al., 2019                 371.1765 0.0136 1.3567 -0.0656
## Malkoc et al., 2023              373.2067 0.0134 1.3370 -0.0534
## Malkoc et al., 2024              376.1061 0.0134 1.3370 -0.0149
## Moe & Putwain, 2020.1            374.7188 0.0081 0.8085  0.1016
## Moe & Putwain, 2020.2            376.8328 0.0081 0.8085  0.0463
## Moe & Putwain, 2020.3            376.5738 0.0081 0.8085  0.0577
## Moe & Putwain, 2020.4            376.8905 0.0081 0.8085  0.0430
## Moe & Putwain, 2020.5            376.6981 0.0081 0.8085  0.0527
## Moe & Putwain, 2020.6            375.3578 0.0081 0.8085  0.0899
## Moe & Putwain, 2020.7            376.7189 0.0081 0.8085  0.0518
## Moore et al., 2017               373.7907 0.0100 0.9960  0.1134
## Mosley et al., 2018.1            374.9393 0.0073 0.7290 -0.0854
## Mosley et al., 2018.2            376.4897 0.0073 0.7290 -0.0386
## Mulvenna et al., 2023.1          374.6075 0.0129 1.2946 -0.0458
## Mulvenna et al., 2023.2          376.0461 0.0129 1.2946 -0.0216
## O'Brien et al., 2022             370.7560 0.0080 0.7953 -0.1621
## Sammy et al., 2017               371.9932 0.0077 0.7673  0.1369
## Schickel et al., 2023.1          377.0414 0.0106 1.0634  0.0016
## Schickel et al., 2023.2          375.9794 0.0106 1.0634  0.0744
## Sharpe et al., 2024.1            377.0726 0.0057 0.5731  0.0241
## Sharpe et al., 2024.2            372.9904 0.0079 0.7885 -0.1246
## Sharpe et al., 2024.3            371.5501 0.0051 0.5103  0.1315
## Thornton et al., 2020.1          376.4190 0.0106 1.0557 -0.0304
## Thornton et al., 2020.2          375.7601 0.0106 1.0557 -0.0503
## Trotman et al., 2018.1           376.5610 0.0091 0.9080  0.0589
## Trotman et al., 2018.2           376.8810 0.0091 0.9080 -0.0150
## Turner et al., 2021.1            377.1292 0.0097 0.9673  0.0185
## Turner et al., 2021.2            375.0714 0.0097 0.9673  0.0946
## Turner et al., 2021.3            377.0060 0.0097 0.9673 -0.0043
## Wood et al., 2018                375.2492 0.0060 0.5960  0.0874
## Scheepers & Keller, 2022         375.9007 0.0113 1.1277  0.0743
## Bosshard et al., 2023.1          376.6850 0.0122 1.2198  0.0510
## Mansell, 2023.1                  371.6856 0.0106 1.0557 -0.1263
## Mansell, 2023.2                  369.9919 0.0106 1.0557 -0.1502
## Jamieson et al., 2021.1          357.7120 0.0134 1.3411  0.1625
## Jamieson et al., 2021.2          369.5351 0.0134 1.3411  0.1140
## Sharpe et al., 2024.4            374.1431 0.0042 0.4227 -0.0964
## Sharpe et al., 2024.5            375.6769 0.0042 0.4227 -0.0634
## Sharpe et al., 2024.6            376.9371 0.0042 0.4227 -0.0177
## Sharpe et al., 2024.7            376.5420 0.0042 0.4227 -0.0369
## Sharpe et al., 2024.8            374.3001 0.0042 0.4227 -0.0934
## Sharpe et al., 2024.9            372.7159 0.0042 0.4227 -0.1207
## Sharpe et al., 2024.10           376.5750 0.0042 0.4227 -0.0356
## Sharpe et al., 2024.11           376.4942 0.0042 0.4227 -0.0387
## Sharpe et al., 2024.12           376.8276 0.0019 0.1898 -0.0198
## Sharpe et al., 2024.13           376.3362 0.0019 0.1898 -0.0345
## Sharpe et al., 2024.14           377.1225 0.0019 0.1898  0.0069
## Sharpe et al., 2024.15           377.0577 0.0019 0.1898 -0.0079
## Simms, 2022.1                    377.1225 0.0058 0.5847  0.0153
## Simms, 2022.2                    377.0001 0.0058 0.5847  0.0314
## Simms, 2022.3                    376.8966 0.0058 0.5847 -0.0197
## Simms, 2022.4                    376.6692 0.0058 0.5847 -0.0322
## Simms, 2022.5                    377.1191 0.0058 0.5847  0.0037
## Simms, 2022.6                    375.0108 0.0058 0.5847 -0.0839
## Marr et al., 2021                376.4851 0.0117 1.1666  0.0587
```

```
## Conlon et al., 2022.1             372.9087 0.0085 0.8510  0.1270
## Conlon et al., 2022.2             375.6734 0.0085 0.8510  0.0835
## van Gog et al., 2024.1            376.1680 0.0088 0.8783 -0.0470
## van Gog et al., 2024.2            375.9976 0.0088 0.8783 -0.0525
## van Gog et al., 2024.3            374.9298 0.0080 0.8020 -0.0840
## van Gog et al., 2024.4            376.0623 0.0080 0.8020 -0.0528
## van Gog et al., 2024.5            376.7364 0.0077 0.7673 -0.0266
## van Gog et al., 2024.6            377.1206 0.0077 0.7673  0.0192
## van Gog et al., 2024.7            376.5062 0.0086 0.8567 -0.0351
## van Gog et al., 2024.8            373.7382 0.0086 0.8567 -0.1077
## van Gog et al., 2024.9            371.1049 0.0077 0.7745 -0.1572
## van Gog et al., 2024.10           376.9044 0.0077 0.7745  0.0417
## van Gog et al., 2024.11           376.5941 0.0073 0.7290 -0.0341
## van Gog et al., 2024.12           376.7752 0.0073 0.7290 -0.0252
## van Gog et al., 2024.13           375.2250 0.0083 0.8274 -0.0759
## van Gog et al., 2024.14           376.1459 0.0083 0.8274 -0.0493
## van Gog et al., 2024.15           372.6448 0.0087 0.8677 -0.1275
## van Gog et al., 2024.16           377.0808 0.0087 0.8677  0.0013
## van Gog et al., 2024.17           375.4537 0.0087 0.8677 -0.0685
## van Gog et al., 2024.18           369.8239 0.0087 0.8677 -0.1727
## van Gog et al., 2024.19           375.8325 0.0087 0.8730 -0.0577
## van Gog et al., 2024.20           377.1173 0.0087 0.8730  0.0218
```

```
ranktest(model_fun_Cogni)
```

```
## Warning in cor.test.default(yi.star, vi, method = "kendall", exact = exact):
## nie można obliczyć dokładnej wartości prawdopodobieństwa z powtórzonymi
## wartościami
```

```
##
## Rank Correlation Test for Funnel Plot Asymmetry
##
## Kendall's tau = -0.1557, p = 0.0121
```

```
# leave one out analysis #

leave1out(model_fun_Cogni)
```

```
## 
##                                       estimate     se   zval   pval  ci.lb  ci.ub
## Baumgartner & Schneider, 2023           0.1412 0.0178 7.9500 0.0000 0.1064 0.1761
## Behnke et al., 2020.1                   0.1408 0.0177 7.9452 0.0000 0.1060 0.1755
## Behnke et al., 2020.2                   0.1420 0.0178 7.9815 0.0000 0.1071 0.1768
## Behnke et al., 2022                     0.1412 0.0178 7.9132 0.0000 0.1062 0.1762
## Behnke et al., 2024.1                   0.1433 0.0178 8.0288 0.0000 0.1083 0.1783
## Brimmell et al., 2019                   0.1410 0.0177 7.9697 0.0000 0.1063 0.1756
## Cabral et al., 2024.1                   0.1425 0.0178 8.0139 0.0000 0.1077 0.1774
## Cabral et al., 2024.2                   0.1423 0.0178 8.0024 0.0000 0.1075 0.1772
## Cabral et al., 2024.3                   0.1426 0.0178 8.0201 0.0000 0.1078 0.1775
## Carenzo et al., 2020.1                  0.1494 0.0170 8.8035 0.0000 0.1161 0.1827
## Carenzo et al., 2020.2                  0.1383 0.0171 8.0983 0.0000 0.1049 0.1718
## Gurera & Isaacowitz, 2022.1             0.1423 0.0178 8.0039 0.0000 0.1075 0.1772
## Gurera & Isaacowitz, 2022.2             0.1410 0.0177 7.9516 0.0000 0.1062 0.1757
## Gurera & Isaacowitz, 2022.3             0.1423 0.0178 8.0136 0.0000 0.1075 0.1770
## Gurera & Isaacowitz, 2022.4             0.1435 0.0177 8.1011 0.0000 0.1088 0.1782
## Hase et al., 2019.1                     0.1429 0.0177 8.0528 0.0000 0.1081 0.1777
## Hase et al., 2019.2                     0.1419 0.0177 7.9951 0.0000 0.1071 0.1767
## Hase et al., 2019.3                     0.1461 0.0175 8.3703 0.0000 0.1119 0.1804
## Hase et al., 2019.4                     0.1446 0.0176 8.1969 0.0000 0.1100 0.1791
## Hase et al., 2019.5                     0.1408 0.0177 7.9542 0.0000 0.1061 0.1755
## Hase et al., 2019.6                     0.1408 0.0177 7.9549 0.0000 0.1061 0.1755
## Hase et al., in preparation.1           0.1458 0.0175 8.3283 0.0000 0.1115 0.1801
## Hase et al., in preparation.2           0.1432 0.0177 8.0752 0.0000 0.1084 0.1779
## Hase et al., in preparation.3           0.1426 0.0177 8.0448 0.0000 0.1079 0.1773
## Hase et al., in preparation.4           0.1415 0.0177 7.9918 0.0000 0.1068 0.1762
## Hase et al., in preparation.5           0.1411 0.0177 7.9808 0.0000 0.1064 0.1757
## Hase et al., in preparation.6           0.1447 0.0176 8.2170 0.0000 0.1102 0.1792
## Hase et al., in preparation.7           0.1435 0.0177 8.1112 0.0000 0.1088 0.1782
## Hase et al., in preparation.8           0.1442 0.0176 8.1734 0.0000 0.1096 0.1788
## Hase et al., in preparation.9           0.1435 0.0177 8.1154 0.0000 0.1089 0.1782
## Hase et al., in preparation.10          0.1417 0.0177 8.0016 0.0000 0.1070 0.1764
## Hase et al., in preparation.11          0.1434 0.0177 8.1048 0.0000 0.1087 0.1781
## Hase et al., in preparation.12          0.1427 0.0177 8.0547 0.0000 0.1080 0.1774
## Hase et al., in preparation.13          0.1425 0.0177 8.0406 0.0000 0.1078 0.1772
## Hase et al., in preparation.14          0.1429 0.0177 8.0701 0.0000 0.1082 0.1776
## Jamieson et al., 2022.1                 0.1394 0.0176 7.9385 0.0000 0.1050 0.1739
## Jamieson et al., 2022.2                 0.1389 0.0174 7.9933 0.0000 0.1048 0.1730
## Jamieson et al., 2022.3                 0.1396 0.0176 7.9460 0.0000 0.1052 0.1741
## Journault et al., in preparation.1      0.1395 0.0176 7.9381 0.0000 0.1050 0.1739
## Journault et al., in preparation.2      0.1415 0.0178 7.9335 0.0000 0.1065 0.1764
## Journault et al., in preparation.3      0.1404 0.0177 7.9137 0.0000 0.1056 0.1752
## Khalaf et al., 2020.1                   0.1404 0.0177 7.9362 0.0000 0.1057 0.1751
## Khalaf et al., 2020.2                   0.1406 0.0177 7.9362 0.0000 0.1059 0.1753
## Laurin & Pellet, 2023                   0.1427 0.0179 7.9837 0.0000 0.1076 0.1777
## Lee et al., 2019                        0.1437 0.0178 8.0698 0.0000 0.1088 0.1786
## Malkoc et al., 2023                     0.1435 0.0178 8.0529 0.0000 0.1086 0.1784
## Malkoc et al., 2024                     0.1428 0.0178 8.0013 0.0000 0.1078 0.1778
## Moe & Putwain, 2020.1                   0.1408 0.0177 7.9551 0.0000 0.1061 0.1754
## Moe & Putwain, 2020.2                   0.1417 0.0178 7.9808 0.0000 0.1069 0.1765
## Moe & Putwain, 2020.3                   0.1415 0.0178 7.9730 0.0000 0.1067 0.1763
## Moe & Putwain, 2020.4                   0.1418 0.0178 7.9833 0.0000 0.1070 0.1766
## Moe & Putwain, 2020.5                   0.1416 0.0178 7.9763 0.0000 0.1068 0.1764
## Moe & Putwain, 2020.6                   0.1410 0.0177 7.9578 0.0000 0.1062 0.1757
```

```
## Moe & Putwain, 2020.7              0.1416 0.0178 7.9769 0.0000 0.1068 0.1764
## Moore et al., 2017                 0.1406 0.0177 7.9380 0.0000 0.1059 0.1753
## Mosley et al., 2018.1              0.1441 0.0177 8.1467 0.0000 0.1094 0.1787
## Mosley et al., 2018.2              0.1432 0.0177 8.0775 0.0000 0.1085 0.1780
## Mulvenna et al., 2023.1            0.1434 0.0178 8.0452 0.0000 0.1084 0.1783
## Mulvenna et al., 2023.2            0.1429 0.0178 8.0127 0.0000 0.1080 0.1779
## O'Brien et al., 2022               0.1454 0.0176 8.2780 0.0000 0.1110 0.1798
## Sammy et al., 2017                 0.1402 0.0176 7.9613 0.0000 0.1056 0.1747
## Schickel et al., 2023.1            0.1425 0.0178 8.0026 0.0000 0.1076 0.1774
## Schickel et al., 2023.2            0.1412 0.0178 7.9430 0.0000 0.1064 0.1761
## Sharpe et al., 2024.1              0.1421 0.0177 8.0179 0.0000 0.1074 0.1769
## Sharpe et al., 2024.2              0.1447 0.0176 8.2088 0.0000 0.1102 0.1793
## Sharpe et al., 2024.3              0.1402 0.0176 7.9825 0.0000 0.1058 0.1747
## Thornton et al., 2020.1            0.1431 0.0178 8.0419 0.0000 0.1082 0.1780
## Thornton et al., 2020.2            0.1434 0.0178 8.0692 0.0000 0.1086 0.1783
## Trotman et al., 2018.1             0.1415 0.0178 7.9642 0.0000 0.1067 0.1763
## Trotman et al., 2018.2             0.1428 0.0178 8.0335 0.0000 0.1080 0.1777
## Turner et al., 2021.1              0.1422 0.0178 7.9923 0.0000 0.1073 0.1771
## Turner et al., 2021.2              0.1409 0.0177 7.9432 0.0000 0.1061 0.1756
## Turner et al., 2021.3              0.1426 0.0178 8.0165 0.0000 0.1078 0.1775
## Wood et al., 2018                  0.1410 0.0177 7.9763 0.0000 0.1064 0.1757
## Scheepers & Keller, 2022           0.1412 0.0178 7.9378 0.0000 0.1064 0.1761
## Bosshard et al., 2023.1            0.1416 0.0178 7.9448 0.0000 0.1067 0.1766
## Mansell, 2023.1                    0.1448 0.0177 8.1924 0.0000 0.1101 0.1794
## Mansell, 2023.2                    0.1452 0.0176 8.2364 0.0000 0.1106 0.1797
## Jamieson et al., 2021.1            0.1397 0.0176 7.9221 0.0000 0.1051 0.1743
## Jamieson et al., 2021.2            0.1405 0.0178 7.9087 0.0000 0.1057 0.1754
## Sharpe et al., 2024.4              0.1442 0.0176 8.1872 0.0000 0.1097 0.1788
## Sharpe et al., 2024.5              0.1437 0.0177 8.1357 0.0000 0.1091 0.1783
## Sharpe et al., 2024.6              0.1429 0.0177 8.0740 0.0000 0.1082 0.1775
## Sharpe et al., 2024.7              0.1432 0.0177 8.0984 0.0000 0.1085 0.1779
## Sharpe et al., 2024.8              0.1442 0.0176 8.1824 0.0000 0.1097 0.1787
## Sharpe et al., 2024.9              0.1447 0.0176 8.2286 0.0000 0.1102 0.1791
## Sharpe et al., 2024.10             0.1432 0.0177 8.0967 0.0000 0.1085 0.1778
## Sharpe et al., 2024.11             0.1432 0.0177 8.1008 0.0000 0.1086 0.1779
## Sharpe et al., 2024.12             0.1429 0.0177 8.0946 0.0000 0.1083 0.1775
## Sharpe et al., 2024.13             0.1432 0.0176 8.1131 0.0000 0.1086 0.1777
## Sharpe et al., 2024.14             0.1424 0.0177 8.0648 0.0000 0.1078 0.1770
## Sharpe et al., 2024.15             0.1427 0.0177 8.0807 0.0000 0.1081 0.1773
## Simms, 2022.1                      0.1423 0.0177 8.0252 0.0000 0.1075 0.1770
## Simms, 2022.2                      0.1420 0.0177 8.0106 0.0000 0.1073 0.1767
## Simms, 2022.3                      0.1429 0.0177 8.0640 0.0000 0.1082 0.1776
## Simms, 2022.4                      0.1431 0.0177 8.0798 0.0000 0.1084 0.1778
## Simms, 2022.5                      0.1425 0.0177 8.0372 0.0000 0.1077 0.1772
## Simms, 2022.6                      0.1440 0.0177 8.1550 0.0000 0.1094 0.1786
## Marr et al., 2021                  0.1415 0.0178 7.9437 0.0000 0.1066 0.1764
## Conlon et al., 2022.1              0.1403 0.0176 7.9514 0.0000 0.1057 0.1749
## Conlon et al., 2022.2              0.1411 0.0177 7.9564 0.0000 0.1063 0.1758
## van Gog et al., 2024.1             0.1434 0.0178 8.0777 0.0000 0.1086 0.1782
## van Gog et al., 2024.2             0.1435 0.0177 8.0856 0.0000 0.1087 0.1783
## van Gog et al., 2024.3             0.1440 0.0177 8.1391 0.0000 0.1093 0.1787
## van Gog et al., 2024.4             0.1435 0.0177 8.0917 0.0000 0.1087 0.1782
## van Gog et al., 2024.5             0.1430 0.0177 8.0587 0.0000 0.1082 0.1778
## van Gog et al., 2024.6             0.1422 0.0178 8.0072 0.0000 0.1074 0.1770
## van Gog et al., 2024.7             0.1432 0.0178 8.0630 0.0000 0.1084 0.1780
## van Gog et al., 2024.8             0.1444 0.0177 8.1743 0.0000 0.1098 0.1791
```

```
## van Gog et al., 2024.9            0.1453 0.0176 8.2701 0.0000 0.1109 0.1797
## van Gog et al., 2024.10           0.1418 0.0178 7.9870 0.0000 0.1070 0.1766
## van Gog et al., 2024.11           0.1432 0.0177 8.0714 0.0000 0.1084 0.1779
## van Gog et al., 2024.12           0.1430 0.0177 8.0598 0.0000 0.1082 0.1778
## van Gog et al., 2024.13           0.1439 0.0177 8.1245 0.0000 0.1092 0.1786
## van Gog et al., 2024.14           0.1434 0.0177 8.0848 0.0000 0.1087 0.1782
## van Gog et al., 2024.15           0.1448 0.0176 8.2083 0.0000 0.1102 0.1794
## van Gog et al., 2024.16           0.1425 0.0178 8.0178 0.0000 0.1077 0.1774
## van Gog et al., 2024.17           0.1438 0.0177 8.1101 0.0000 0.1090 0.1785
## van Gog et al., 2024.18           0.1456 0.0176 8.2933 0.0000 0.1112 0.1800
## van Gog et al., 2024.19           0.1436 0.0177 8.0936 0.0000 0.1088 0.1783
## van Gog et al., 2024.20           0.1422 0.0178 7.9964 0.0000 0.1073 0.1770
##                                          Q     Qp   tau2      I2     H2
## Baumgartner & Schneider, 2023     376.0458 0.0000 0.0211 72.4433 3.6289
## Behnke et al., 2020.1             374.7321 0.0000 0.0209 72.3024 3.6104
## Behnke et al., 2020.2             377.0359 0.0000 0.0212 72.5477 3.6427
## Behnke et al., 2022               371.4928 0.0000 0.0212 71.0777 3.4575
## Behnke et al., 2024.1             360.0503 0.0000 0.0212 69.6185 3.2915
## Brimmell et al., 2019             375.2546 0.0000 0.0209 72.3371 3.6150
## Cabral et al., 2024.1             377.0734 0.0000 0.0212 72.5518 3.6432
## Cabral et al., 2024.2             377.1268 0.0000 0.0212 72.5575 3.6440
## Cabral et al., 2024.3             377.0228 0.0000 0.0212 72.5464 3.6425
## Carenzo et al., 2020.1            341.0679 0.0000 0.0181 69.1888 3.2456
## Carenzo et al., 2020.2            345.7446 0.0000 0.0185 69.6520 3.2951
## Gurera & Isaacowitz, 2022.1       377.1294 0.0000 0.0212 72.5573 3.6440
## Gurera & Isaacowitz, 2022.2       375.3689 0.0000 0.0210 72.3669 3.6189
## Gurera & Isaacowitz, 2022.3       377.1242 0.0000 0.0211 72.5421 3.6419
## Gurera & Isaacowitz, 2022.4       376.0585 0.0000 0.0210 72.4137 3.6250
## Hase et al., 2019.1               376.8703 0.0000 0.0211 72.5118 3.6379
## Hase et al., 2019.2               376.9498 0.0000 0.0211 72.5248 3.6396
## Hase et al., 2019.3               367.8462 0.0000 0.0200 71.4512 3.5028
## Hase et al., 2019.4               373.5908 0.0000 0.0207 72.1259 3.5876
## Hase et al., 2019.5               374.7727 0.0000 0.0209 72.2950 3.6095
## Hase et al., 2019.6               374.9992 0.0000 0.0209 72.3203 3.6128
## Hase et al., in preparation.1     369.3800 0.0000 0.0202 71.6311 3.5250
## Hase et al., in preparation.2     376.5717 0.0000 0.0210 72.4726 3.6327
## Hase et al., in preparation.3     377.0886 0.0000 0.0211 72.5126 3.6380
## Hase et al., in preparation.4     376.3613 0.0000 0.0210 72.4456 3.6292
## Hase et al., in preparation.5     375.4259 0.0000 0.0209 72.3512 3.6168
## Hase et al., in preparation.6     373.2222 0.0000 0.0206 72.0759 3.5811
## Hase et al., in preparation.7     376.1049 0.0000 0.0209 72.3963 3.6227
## Hase et al., in preparation.8     374.5367 0.0000 0.0208 72.2218 3.5999
## Hase et al., in preparation.9     376.0320 0.0000 0.0209 72.3865 3.6214
## Hase et al., in preparation.10    376.6744 0.0000 0.0210 72.4731 3.6328
## Hase et al., in preparation.11    376.2748 0.0000 0.0210 72.4117 3.6247
## Hase et al., in preparation.12    377.0408 0.0000 0.0210 72.4994 3.6363
## Hase et al., in preparation.13    377.1226 0.0000 0.0211 72.5105 3.6378
## Hase et al., in preparation.14    376.8717 0.0000 0.0210 72.4792 3.6336
## Jamieson et al., 2022.1           356.9870 0.0000 0.0202 71.2499 3.4782
## Jamieson et al., 2022.2           352.6334 0.0000 0.0195 70.7010 3.4131
## Jamieson et al., 2022.3           366.8627 0.0000 0.0203 71.6155 3.5230
## Journault et al., in preparation.1 358.8824 0.0000 0.0202 71.3185 3.4866
## Journault et al., in preparation.2 376.2039 0.0000 0.0212 72.3036 3.6106
## Journault et al., in preparation.3 370.1425 0.0000 0.0209 71.9618 3.5666
## Khalaf et al., 2020.1             373.0985 0.0000 0.0208 72.1490 3.5905
## Khalaf et al., 2020.2             373.9681 0.0000 0.0209 72.2359 3.6018
```

```
## Laurin & Pellet, 2023              374.9994 0.0000 0.0213 70.9100 3.4376
## Lee et al., 2019                   371.1765 0.0000 0.0211 71.9166 3.5608
## Malkoc et al., 2023                373.2067 0.0000 0.0212 72.0585 3.5789
## Malkoc et al., 2024                376.1061 0.0000 0.0213 72.1678 3.5930
## Moe & Putwain, 2020.1              374.7188 0.0000 0.0209 72.2878 3.6085
## Moe & Putwain, 2020.2              376.8328 0.0000 0.0211 72.5218 3.6392
## Moe & Putwain, 2020.3              376.5738 0.0000 0.0211 72.4935 3.6355
## Moe & Putwain, 2020.4              376.8905 0.0000 0.0211 72.5280 3.6401
## Moe & Putwain, 2020.5              376.6981 0.0000 0.0211 72.5071 3.6373
## Moe & Putwain, 2020.6              375.3578 0.0000 0.0209 72.3590 3.6178
## Moe & Putwain, 2020.7              376.7189 0.0000 0.0211 72.5094 3.6376
## Moore et al., 2017                 373.7907 0.0000 0.0209 72.2141 3.5989
## Mosley et al., 2018.1              374.9393 0.0000 0.0209 72.2858 3.6083
## Mosley et al., 2018.2              376.4897 0.0000 0.0210 72.4668 3.6320
## Mulvenna et al., 2023.1            374.6075 0.0000 0.0212 72.2176 3.5994
## Mulvenna et al., 2023.2            376.0461 0.0000 0.0213 72.2856 3.6082
## O'Brien et al., 2022               370.7560 0.0000 0.0204 71.8106 3.5474
## Sammy et al., 2017                 371.9932 0.0000 0.0205 71.9767 3.5685
## Schickel et al., 2023.1            377.0414 0.0000 0.0212 72.5347 3.6410
## Schickel et al., 2023.2            375.9794 0.0000 0.0211 72.4315 3.6273
## Sharpe et al., 2024.1              377.0726 0.0000 0.0211 72.5159 3.6385
## Sharpe et al., 2024.2              372.9904 0.0000 0.0206 72.0702 3.5804
## Sharpe et al., 2024.3              371.5501 0.0000 0.0205 71.9701 3.5676
## Thornton et al., 2020.1            376.4190 0.0000 0.0212 72.4809 3.6338
## Thornton et al., 2020.2            375.7601 0.0000 0.0211 72.4207 3.6259
## Trotman et al., 2018.1             376.5610 0.0000 0.0211 72.4971 3.6360
## Trotman et al., 2018.2             376.8810 0.0000 0.0212 72.5309 3.6405
## Turner et al., 2021.1              377.1292 0.0000 0.0212 72.5559 3.6438
## Turner et al., 2021.2              375.0714 0.0000 0.0210 72.3423 3.6156
## Turner et al., 2021.3              377.0060 0.0000 0.0212 72.5439 3.6422
## Wood et al., 2018                  375.2492 0.0000 0.0209 72.3338 3.6145
## Scheepers & Keller, 2022           375.9007 0.0000 0.0211 72.4113 3.6247
## Bosshard et al., 2023.1            376.6850 0.0000 0.0212 72.4175 3.6255
## Mansell, 2023.1                    371.6856 0.0000 0.0207 72.0380 3.5763
## Mansell, 2023.2                    369.9919 0.0000 0.0205 71.8746 3.5555
## Jamieson et al., 2021.1            357.7120 0.0000 0.0205 71.3553 3.4910
## Jamieson et al., 2021.2            369.5351 0.0000 0.0210 71.8600 3.5537
## Sharpe et al., 2024.4              374.1431 0.0000 0.0207 72.1880 3.5956
## Sharpe et al., 2024.5              375.6769 0.0000 0.0209 72.3371 3.6149
## Sharpe et al., 2024.6              376.9371 0.0000 0.0210 72.4644 3.6317
## Sharpe et al., 2024.7              376.5420 0.0000 0.0210 72.4232 3.6262
## Sharpe et al., 2024.8              374.3001 0.0000 0.0207 72.2032 3.5975
## Sharpe et al., 2024.9              372.7159 0.0000 0.0206 72.0510 3.5780
## Sharpe et al., 2024.10             376.5750 0.0000 0.0210 72.4266 3.6267
## Sharpe et al., 2024.11             376.4942 0.0000 0.0210 72.4184 3.6256
## Sharpe et al., 2024.12             376.8276 0.0000 0.0209 72.4098 3.6245
## Sharpe et al., 2024.13             376.3362 0.0000 0.0209 72.3797 3.6205
## Sharpe et al., 2024.14             377.1225 0.0000 0.0210 72.4348 3.6278
## Sharpe et al., 2024.15             377.0577 0.0000 0.0209 72.4258 3.6266
## Simms, 2022.1                      377.1225 0.0000 0.0211 72.5216 3.6392
## Simms, 2022.2                      377.0001 0.0000 0.0211 72.5114 3.6379
## Simms, 2022.3                      376.8966 0.0000 0.0210 72.4911 3.6352
## Simms, 2022.4                      376.6692 0.0000 0.0210 72.4643 3.6317
## Simms, 2022.5                      377.1191 0.0000 0.0211 72.5192 3.6389
## Simms, 2022.6                      375.0108 0.0000 0.0208 72.2760 3.6070
## Marr et al., 2021                  376.4851 0.0000 0.0212 72.4430 3.6288
```

```
## Conlon et al., 2022.1             372.9087 0.0000 0.0207 72.0907 3.5830
## Conlon et al., 2022.2             375.6734 0.0000 0.0210 72.3976 3.6229
## van Gog et al., 2024.1            376.1680 0.0000 0.0211 72.4506 3.6298
## van Gog et al., 2024.2            375.9976 0.0000 0.0210 72.4317 3.6274
## van Gog et al., 2024.3            374.9298 0.0000 0.0209 72.2978 3.6098
## van Gog et al., 2024.4            376.0623 0.0000 0.0210 72.4281 3.6269
## van Gog et al., 2024.5            376.7364 0.0000 0.0211 72.5013 3.6365
## van Gog et al., 2024.6            377.1206 0.0000 0.0211 72.5484 3.6428
## van Gog et al., 2024.7            376.5062 0.0000 0.0211 72.4858 3.6345
## van Gog et al., 2024.8            373.7382 0.0000 0.0208 72.1739 3.5938
## van Gog et al., 2024.9            371.1049 0.0000 0.0204 71.8460 3.5519
## van Gog et al., 2024.10           376.9044 0.0000 0.0211 72.5266 3.6399
## van Gog et al., 2024.11           376.5941 0.0000 0.0211 72.4791 3.6336
## van Gog et al., 2024.12           376.7752 0.0000 0.0211 72.5005 3.6364
## van Gog et al., 2024.13           375.2250 0.0000 0.0209 72.3365 3.6149
## van Gog et al., 2024.14           376.1459 0.0000 0.0210 72.4414 3.6286
## van Gog et al., 2024.15           372.6448 0.0000 0.0206 72.0532 3.5782
## van Gog et al., 2024.16           377.0808 0.0000 0.0212 72.5511 3.6431
## van Gog et al., 2024.17           375.4537 0.0000 0.0210 72.3696 3.6192
## van Gog et al., 2024.18           369.8239 0.0000 0.0203 71.7300 3.5373
## van Gog et al., 2024.19           375.8325 0.0000 0.0210 72.4127 3.6249
## van Gog et al., 2024.20           377.1173 0.0000 0.0212 72.5559 3.6438
```

```
# Egger#
model_fun_Cogni_OutEgger <- rma.mv(yi = Cogni_cor_Z, V = Cogni_cor_Z_var, random = ~ 1
| paper_id/effect_size_id, mod = ~sqrt(Cogni_cor_Z_var), tdist=TRUE, data = Data)
```

```
## Warning: 41 rows with NAs omitted from model fitting.
```

```
model_fun_Cogni_OutEgger
```

```
##
## Multivariate Meta-Analysis Model (k = 121; method: REML)
##
## Variance Components:
##
##             estim    sqrt  nlvls  fixed                  factor
## sigma^2.1  0.0105  0.1024     39     no                paper_id
## sigma^2.2  0.0098  0.0989    121     no  paper_id/effect_size_id
##
## Test for Residual Heterogeneity:
## QE(df = 119) = 373.2432, p-val < .0001
##
## Test of Moderators (coefficient 2):
## F(df1 = 1, df2 = 119) = 1.5876, p-val = 0.2101
##
## Model Results:
##
##                         estimate      se     tval   df    pval    ci.lb   ci.ub
## intrcpt                   0.2211  0.0505   4.3756  119  <.0001   0.1210  0.3211
## sqrt(Cogni_cor_Z_var)   -0.5302  0.4208  -1.2600  119  0.2101  -1.3635  0.3030
##
## intrcpt                 ***
## sqrt(Cogni_cor_Z_var)
##
## ---
## Signif. codes:  0 '***' 0.001 '**' 0.01 '*' 0.05 '.' 0.1 ' ' 1
```

```
# Run trim-and-fill analysis for the right side
model_fun_Cogni_tf_right <- trimfill(model_fun_Cogni, side = "right")

# Run trim-and-fill analysis for the left side
model_fun_Cogni_tf_left <- trimfill(model_fun_Cogni, side = "left")

# Print the trim-and-fill model results
print(model_fun_Cogni_tf_right)
```

```
##
## Estimated number of missing studies on the right side: 28 (SE = 7.1995)
##
## Random-Effects Model (k = 149; tau^2 estimator: REML)
##
## tau^2 (estimated amount of total heterogeneity): 0.0350 (SE = 0.0061)
## tau (square root of estimated tau^2 value):      0.1871
## I^2 (total heterogeneity / total variability):   79.41%
## H^2 (total variability / sampling variability):  4.86
##
## Test for Heterogeneity:
## Q(df = 148) = 584.4407, p-val < .0001
##
## Model Results:
##
## estimate      se     zval    pval   ci.lb   ci.ub
##   0.2056  0.0191  10.7476  <.0001  0.1681  0.2430  ***
##
## ---
## Signif. codes:  0 '***' 0.001 '**' 0.01 '*' 0.05 '.' 0.1 ' ' 1
```

```
print(model_fun_Cogni_tf_left)
```

```
##
## Estimated number of missing studies on the left side: 0 (SE = 5.7525)
##
## Random-Effects Model (k = 121; tau^2 estimator: REML)
##
## tau^2 (estimated amount of total heterogeneity): 0.0209 (SE = 0.0046)
## tau (square root of estimated tau^2 value):      0.1445
## I^2 (total heterogeneity / total variability):   72.22%
## H^2 (total variability / sampling variability):  3.60
##
## Test for Heterogeneity:
## Q(df = 120) = 377.1303, p-val < .0001
##
## Model Results:
##
## estimate      se    zval    pval   ci.lb   ci.ub
##   0.1426  0.0176  8.0869  <.0001  0.1080  0.1771  ***
##
## ---
## Signif. codes:  0 '***' 0.001 '**' 0.01 '*' 0.05 '.' 0.1 ' ' 1
```

```
# Combine the number of studies imputed from both sides
total_imputed_studies <- model_fun_Cogni_tf_right$k0 + model_fun_Cogni_tf_left$k0
cat("Total number of imputed studies (both sides) for Cogni:", total_imputed_studies,
"\n")
```

```
## Total number of imputed studies (both sides) for Cogni: 28
```

```
# Generate funnel plots only left
#par(mfrow=c(1, 3))
funnel(model_fun_Cogni, main="Original Model")
```

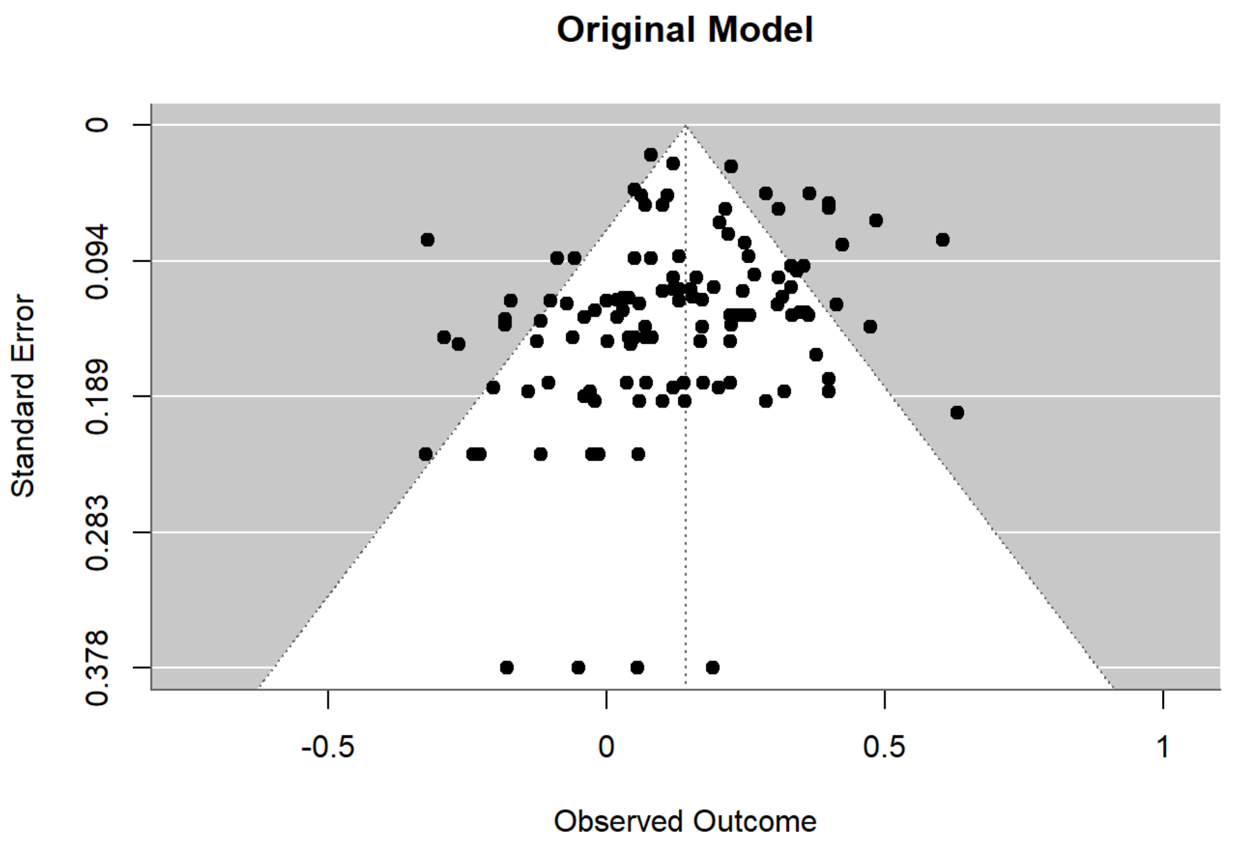


```
funnel(model_fun_Cogni_tf_right, main="Trim-and-Fill Right")
```

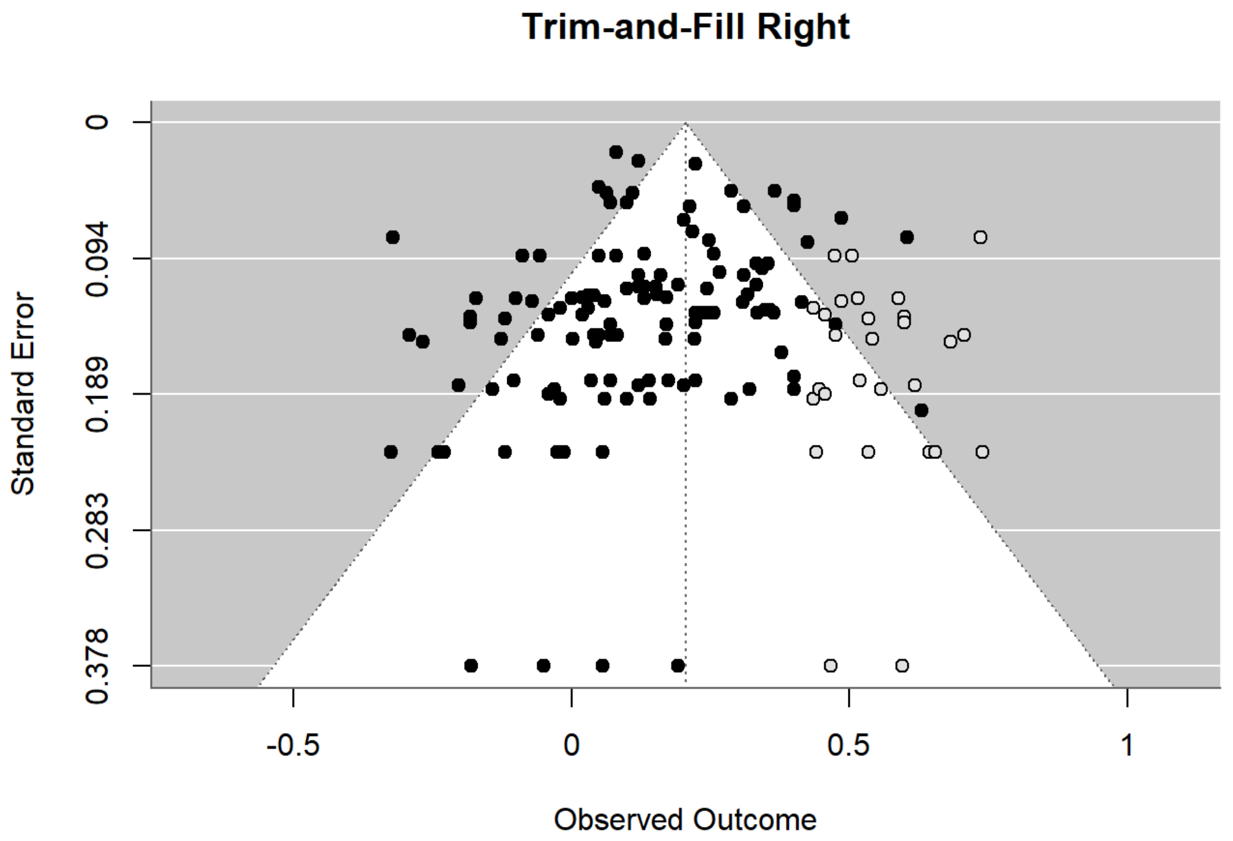


```
funnel(model_fun_Cogni_tf_left, main="Trim-and-Fill Left", xlab = 'Cogni')
```

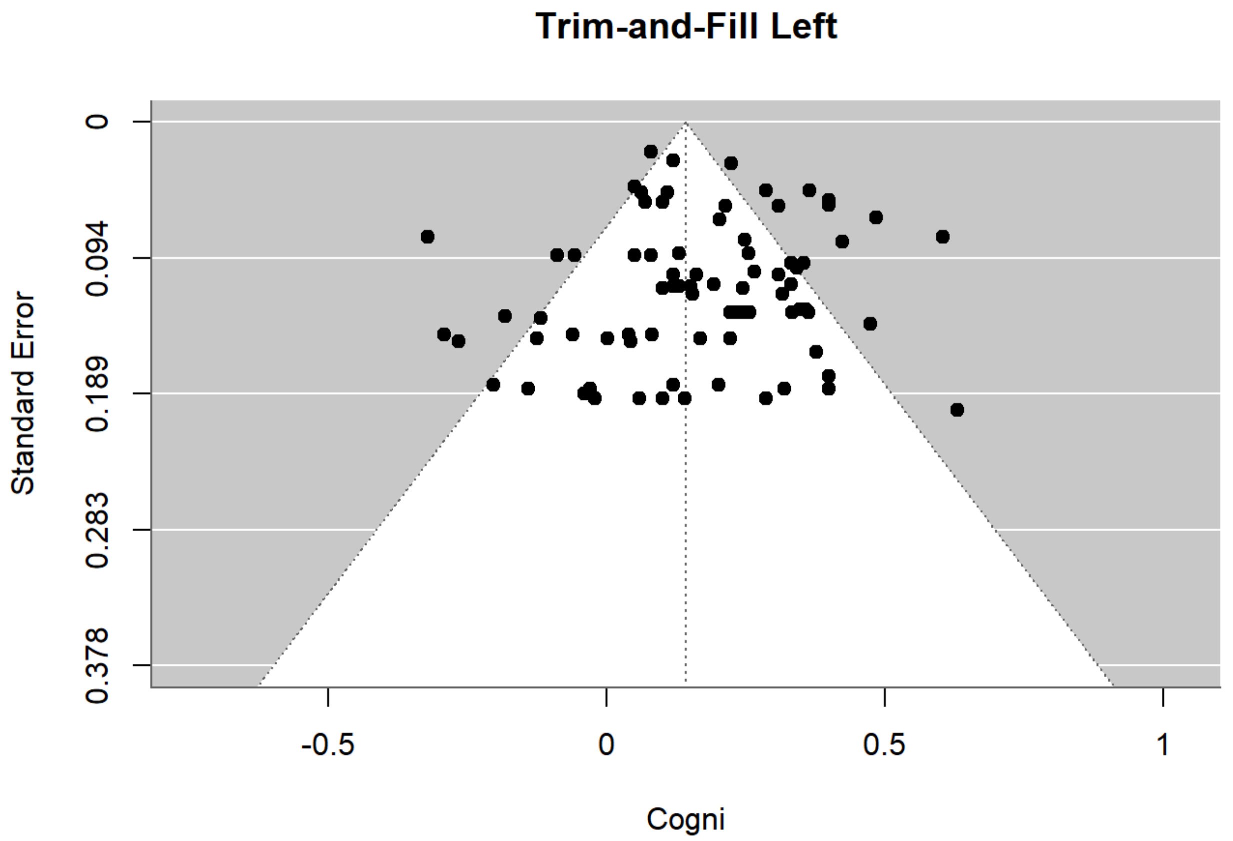

```
# Return to single plot layout
#par(mfrow=c(1, 1))
```

8.2 Multilevel Model

```
model_Cogni_multilevel <- rma.mv(yi = Cogni_cor_Z, V = Cogni_cor_Z_var, slab = Ref_APA,
data = Data, random = ~ 1 | paper_id/effect_size_id, test = "t", method = "REML")
```

```
## Warning: 41 rows with NAs omitted from model fitting.
```

```
model_Cogni_multilevel
```

```
##
## Multivariate Meta-Analysis Model (k = 121; method: REML)
##
## Variance Components:
##
##              estim    sqrt  nlvls  fixed                   factor
## sigma^2.1  0.0122  0.1106     39     no                 paper_id
## sigma^2.2  0.0093  0.0963    121     no  paper_id/effect_size_id
##
## Test for Heterogeneity:
## Q(df = 120) = 377.1303, p-val < .0001
##
## Model Results:
##
## estimate      se    tval   df    pval   ci.lb   ci.ub
##   0.1650  0.0239  6.9040  120  <.0001  0.1177  0.2123  ***
##
## ---
## Signif. codes:  0 '***' 0.001 '**' 0.01 '*' 0.05 '.' 0.1 ' ' 1
```

```
convert_z2r(0.1660)
```

```
## [1] 0.1644919
```

```
predict_Cogni <- predict(model_Cogni_multilevel, digits=3, transf=transf.ztor, level =
95)
predict_Cogni
```

```
##
##   pred ci.lb ci.ub  pi.lb pi.ub
##  0.163 0.117 0.209 -0.128 0.429
```

```
forest.rma(model_Cogni_multilevel, header = "Cogni",slab = Data$Ref_APA, alim=c(-0.8,1.
2))
```

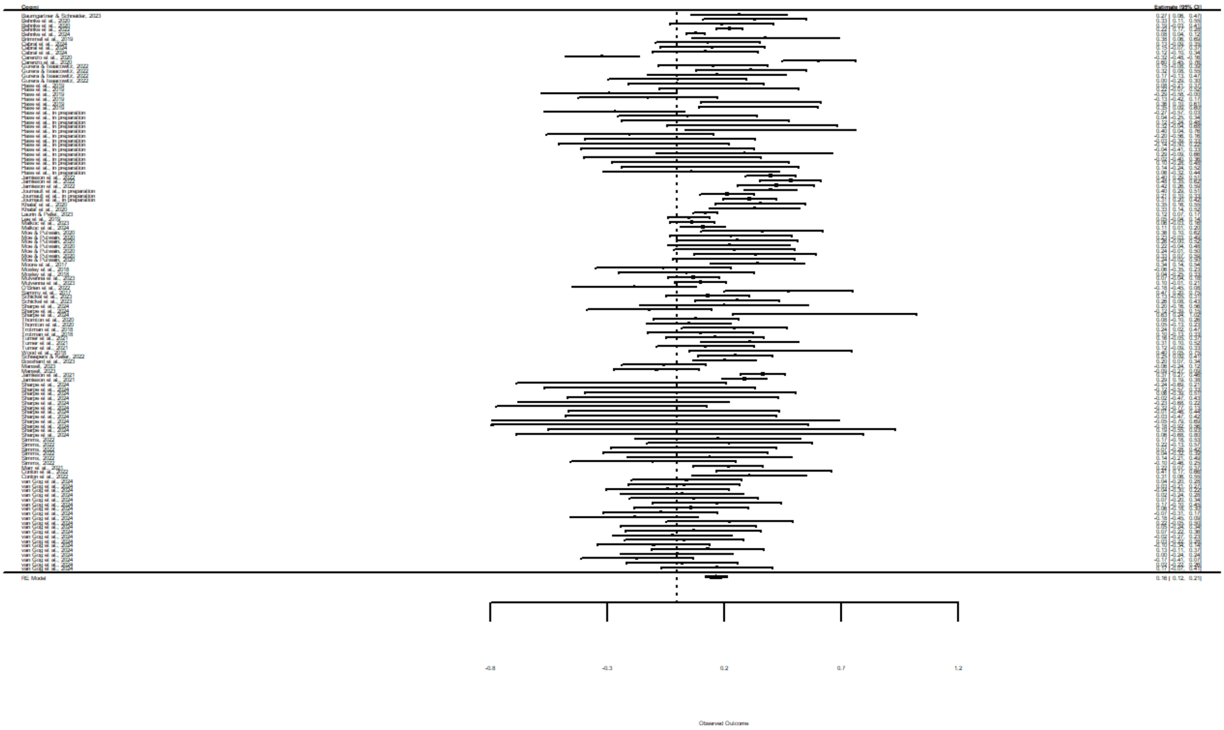

```
######## list 1 ########
list_Cogni <- Data$Cogni_cor_Z_var

############ sum 1#####################
sum_Cogni <- sum(list_Cogni, na.rm = TRUE)

###################### sum 2 #####################
sum2_Cogni <- (sum_Cogni)^2

####################### list 2 ##############
list_In_Cogni <- Data$Cogni_cor_Z_var_Sq

#################### sum 3 ######################
sum_In_Cogni<- sum(list_In_Cogni, na.rm = TRUE)

################ numerator ##############
numerator_Cogni<- (model_Cogni_multilevel$k-1)*sum_Cogni

############# denominator #############
denominator_Cogni<- sum2_Cogni - sum_In_Cogni

############## eps ################
EPS_Cogni<- numerator_Cogni / denominator_Cogni

############### i2 1 level ##################
I2_1_Cogni <- (EPS_Cogni) / (model_Cogni_multilevel$sigma2[1] + model_Cogni_multilevel$sigma2[2] + EPS_Cogni) *100
I2_1_Cogni
```

```
## [1] 99.94956
```

```
############## i2 2 level #################
I2_2_Cogni <- (model_Cogni_multilevel$sigma2[1]) / (model_Cogni_multilevel$sigma2[1] +
model_Cogni_multilevel$sigma2[2] + EPS_Cogni) *100
I2_2_Cogni
```

```
## [1] 0.02869619
```

```
########### I2 level 3
I2_3_Cogni <- (model_Cogni_multilevel$sigma2[2]) / (model_Cogni_multilevel$sigma2[1] +
model_Cogni_multilevel$sigma2[2] + EPS_Cogni) *100
I2_3_Cogni
```

```
## [1] 0.02174071
```

```
############### ML without level 2 ##########
model_Cogni_multilevel_2 <- rma.mv(yi = Cogni_cor_Z, V = Cogni_cor_Z_var, slab = Ref_AP
A, random = ~ 1 | paper_id/effect_size_id, test = "t", method = "REML", sigma2=c(0,NA),
tdist=TRUE,data = Data)
```

```
## Warning: 41 rows with NAs omitted from model fitting.
```

```
############# ml without level 3 ###########
model_Cogni_multilevel_3 <- rma.mv(yi = Cogni_cor_Z, V = Cogni_cor_Z_var, slab = Ref_AP
A, random = ~ 1 | paper_id/effect_size_id, test = "t", method = "REML", sigma2=c(NA,0),
tdist=TRUE,data = Data)
```

```
## Warning: 41 rows with NAs omitted from model fitting.
```

```
##################### sig level 2 #######################
anova12_Cogni <- anova(model_Cogni_multilevel,model_Cogni_multilevel_2)
anova12_Cogni
```

```
##
##         df      AIC      BIC     AICc  logLik     LRT   pval       QE
## Full     3 -64.3029 -55.9404 -64.0960 35.1515                377.1303
## Reduced  2 -47.4700 -41.8950 -47.3674 25.7350 18.8330 <.0001 377.1303
```

```
###############sig level 3v #################
anova13_Cogni <- anova(model_Cogni_multilevel,model_Cogni_multilevel_3)
anova13_Cogni
```

```
##
##         df      AIC      BIC     AICc  logLik     LRT   pval       QE
## Full     3 -64.3029 -55.9404 -64.0960 35.1515                377.1303
## Reduced  2 -42.8638 -37.2888 -42.7612 23.4319 23.4391 <.0001 377.1303
```

9. Results table

```r
# Function to perform the calculations and create the result table for a given paramete
r
create_result_table <- function(parameter_name, cor_col, n_col) {
  # Z-transform correlation coefficients
  z_col <- .5 * log((1 + Data[[cor_col]]) / (1 - Data[[cor_col]]))
  var_col <- 1 / (Data[[n_col]] - 3)

  # Add z and variance columns to Data
  Data[[paste0(parameter_name, "_cor_Z")]] <- z_col
  Data[[paste0(parameter_name, "_cor_Z_var")]] <- var_col
  Data[[paste0(parameter_name, "_cor_Z_var_Sq")]] <- var_col^2

  # Meta-analysis model
  model <- rma.mv(yi = z_col, V = var_col, slab = Data$Ref_APA,
                  data = Data, random = ~ 1 | paper_id/effect_size_id,
                  test = "t", method = "REML")

  # Prediction intervals
  predict_result <- predict(model, transf=transf.ztor, level = 95)

  # Additional I2 calculations
  EPS <- (model$k - 1) * sum(var_col, na.rm = TRUE) /
         ((sum(var_col, na.rm = TRUE))^2 - sum(var_col^2, na.rm = TRUE))

  I2_1 <- round((EPS) / (model$sigma2[1] + model$sigma2[2] + EPS) * 100, 2)
  I2_2 <- round((model$sigma2[1]) / (model$sigma2[1] + model$sigma2[2] + EPS) * 100, 2)
  I2_3 <- round((model$sigma2[2]) / (model$sigma2[1] + model$sigma2[2] + EPS) * 100, 2)

  # Egger's test
  egger_test <- rma.mv(yi = z_col, V = var_col, random = ~ 1 | paper_id/effect_size_id,
                       mod = ~ sqrt(var_col), tdist = TRUE, data = Data)
  egger_result <- round(egger_test$QM, 2)

  # Rank correlation test
  rank_corr_test <- ranktest(model)
  rank_corr_result <- round(as.numeric(rank_corr_test$tau), 2)

  # Creating the table
  result_table <- data.frame(
    Parameter_Name = parameter_name,
    Number_of_Effect_Sizes = model$k,
    Mean_Effect_Size = round(model$b[1], 2),
    CI_95 = paste0(round(model$ci.lb, 2), ", ", round(model$ci.ub, 2)),
    PI_95 = paste0(round(predict_result$pi.lb, 2), ", ", round(predict_result$pi.ub,
2)),
    Q_Parameter = round(model$QE,2),
    I2_Level1 = I2_1,
    I2_Level2 = I2_2,
    I2_Level3 = I2_3,
    Egger_Test_Result = egger_result,
    Rank_Corr_Test_Result = rank_corr_result
  )

  return(result_table)
}
```

```
# List of parameters and corresponding column names
parameters <- list(
  list(name = "CO", cor_col = "CO_cor", n_col = "n_performance"),
  list(name = "TPR", cor_col = "TPR_cor", n_col = "n_performance"),
  list(name = "CTI", cor_col = "CTI_cor", n_col = "n_performance"),
  list(name = "Cogni", cor_col = "Cogni_cor", n_col = "n_performance")
)

# Apply the function to each parameter and combine the results
result_tables <- lapply(parameters, function(param) {
  create_result_table(param$name, param$cor_col, param$n_col)
})
```

```
## Warning: 94 rows with NAs omitted from model fitting.
## Warning: 94 rows with NAs omitted from model fitting.
```

```
## Warning in cor.test.default(yi.star, vi, method = "kendall", exact = exact):
## nie można obliczyć dokładnej wartości prawdopodobieństwa z powtórzonymi
## wartościami
```

```
## Warning: 101 rows with NAs omitted from model fitting.
## Warning: 101 rows with NAs omitted from model fitting.
```

```
## Warning in cor.test.default(yi.star, vi, method = "kendall", exact = exact):
## nie można obliczyć dokładnej wartości prawdopodobieństwa z powtórzonymi
## wartościami
```

```
## Warning: 89 rows with NAs omitted from model fitting.
## Warning: 89 rows with NAs omitted from model fitting.
```

```
## Warning in cor.test.default(yi.star, vi, method = "kendall", exact = exact):
## nie można obliczyć dokładnej wartości prawdopodobieństwa z powtórzonymi
## wartościami
```

```
## Warning: 41 rows with NAs omitted from model fitting.
## Warning: 41 rows with NAs omitted from model fitting.
```

```
## Warning in cor.test.default(yi.star, vi, method = "kendall", exact = exact):
## nie można obliczyć dokładnej wartości prawdopodobieństwa z powtórzonymi
## wartościami
```

```
# Combine all result tables into one data frame
final_result_table <- do.call(rbind, result_tables)

# Print the final result table
print(final_result_table)
```

```
##   Parameter_Name Number_of_Effect_Sizes Mean_Effect_Size        CI_95
## 1             CO                     68             0.06      0, 0.13
## 2            TPR                     61            -0.10 -0.17, -0.03
## 3            CTI                     73             0.10   0.04, 0.15
## 4          Cogni                    121             0.16   0.12, 0.21
##         PI_95 Q_Parameter I2_Level1 I2_Level2 I2_Level3 Egger_Test_Result
## 1 -0.25, 0.36      151.69     99.85      0.08      0.07              0.03
## 2 -0.35, 0.17      117.86     99.88      0.12      0.00              1.39
## 3 -0.24, 0.42      195.52     99.84      0.00      0.16              0.29
## 4 -0.13, 0.43      377.13     99.93      0.04      0.03              1.59
##   Rank_Corr_Test_Result
## 1                 -0.05
## 2                 -0.01
## 3                  0.04
## 4                 -0.16
```

```
write.csv(final_result_table, "meta_analysis_results.csv", row.names = FALSE)
```

10. Figures

10.1 Funnel plots

```
# Run trim-and-fill analysis for the right side
model_fun_CO_tf_right <- trimfill(model_fun_CO, side = "right")
model_fun_TPR_tf_left <- trimfill(model_fun_TPR, side = "left")
model_fun_CTI_tf_right <- trimfill(model_fun_CTI, side = "right")
#model_fun_Cogni_tf_right <- trimfill(model_fun_Cogni, side = "left" )

# Set up 2x2 plotting area
par(mfrow=c(2, 2))

# Generate funnel plots
funnel(model_fun_CO_tf_right, main="", xlab = 'Cardiac Output')
funnel(model_fun_TPR_tf_left, main="", xlab = 'Total Peripheral Resistance')
funnel(model_fun_CTI_tf_right, main="", xlab = 'Challenge Threat Index')
funnel(model_fun_Cogni_tf_right, main="", xlab = 'Cognitive Evaluations')
```

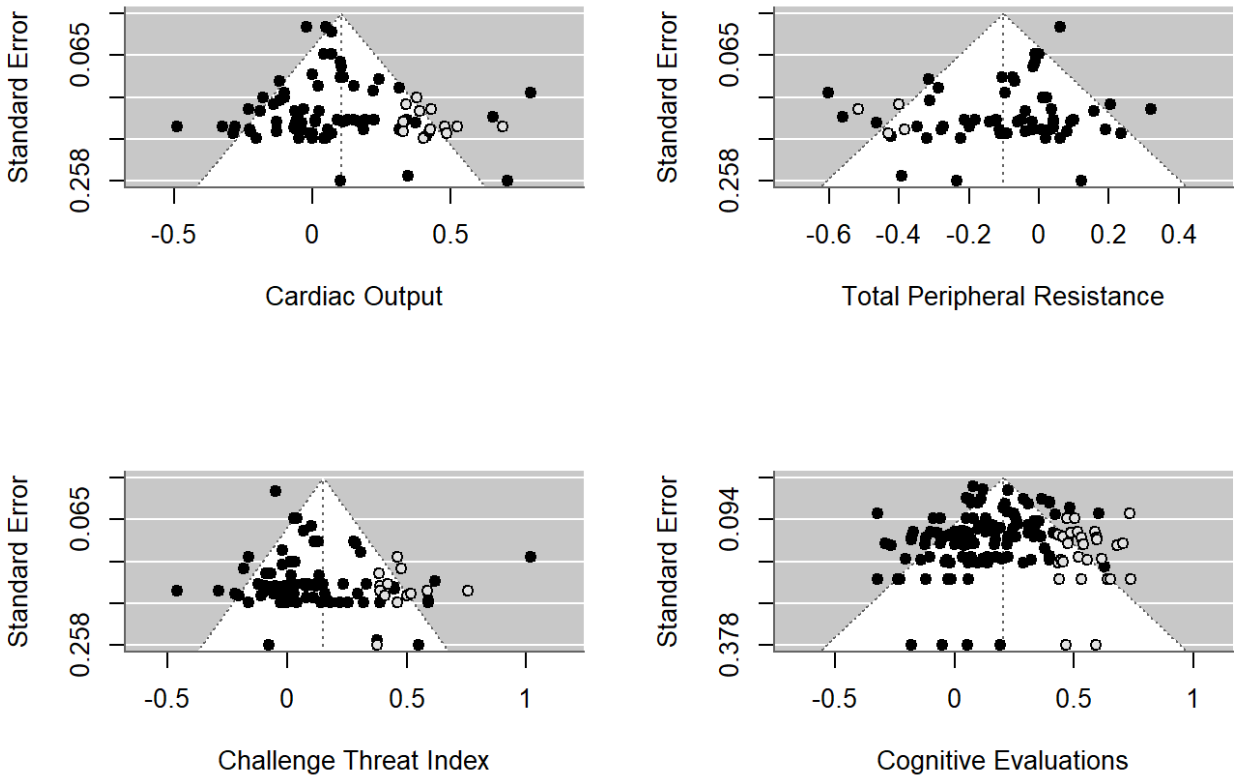


```
# Set up the file for output
jpeg("funnel_plots.jpg", width = 16, height = 16, units = "cm", res = 300)

# Set up 2x2 plotting area
par(mfrow=c(2, 2))

# Generate funnel plots
funnel(model_fun_CO_tf_right, main="", xlab = 'Cardiac Output')
funnel(model_fun_TPR_tf_left, main="", xlab = 'Total Peripheral Resistance')
funnel(model_fun_CTI_tf_right, main="", xlab = 'Challenge Threat Index')
funnel(model_fun_Cogni_tf_right, main="", xlab = 'Cognitive Evaluations')

# Close the file
dev.off()
```

```
## png
##   2
```

```
# Return to single plot layout
par(mfrow=c(1, 1))
```

10.2 Forest plots

```
# Save CO forest plot
jpeg("forest_plot_CO.jpg", width = 8, height = 26, units = "cm", res = 300)
forest(model_CO_multilevel, header = "Reference", xlab = 'CO', slab = Data$Ref_APA, ali
m = c(-0.8, 1), cex = 0.42, shade="zebra")
dev.off()
```

```
## png
##   2
```

```
# Save TPR forest plot
jpeg("forest_plot_TPR.jpg", width = 8, height = 26, units = "cm", res = 300)
forest(model_TPR_multilevel, header = "Reference", xlab = 'TPR', slab = Data$Ref_APA, a
lim = c(-1, .6), cex = 0.42, shade="zebra")
dev.off()
```

```
## png
##   2
```

```
# Save CTI forest plot
jpeg("forest_plot_CTI.jpg", width = 8, height = 26, units = "cm", res = 300)
forest(model_CTI_multilevel, header = "Reference", xlab = 'CTI', slab = Data$Ref_APA, a
lim = c(-1.5, 1.5), cex = 0.42, shade="zebra")
dev.off()
```

```
## png
##   2
```

```
# Save Cogni forest plot
jpeg("forest_plot_Cogni.jpg", width = 8, height = 26, units = "cm", res = 300)
forest(model_Cogni_multilevel, header = "Reference", xlab = 'Cognitive Evaluations', sl
ab = Data$Ref_APA, alim = c(-0.8, 1), cex = 0.42, shade="zebra")
dev.off()
```

```
## png
##   2
```

```
# Display forest plots
knitr::include_graphics("forest_plot_CO.jpg")
```

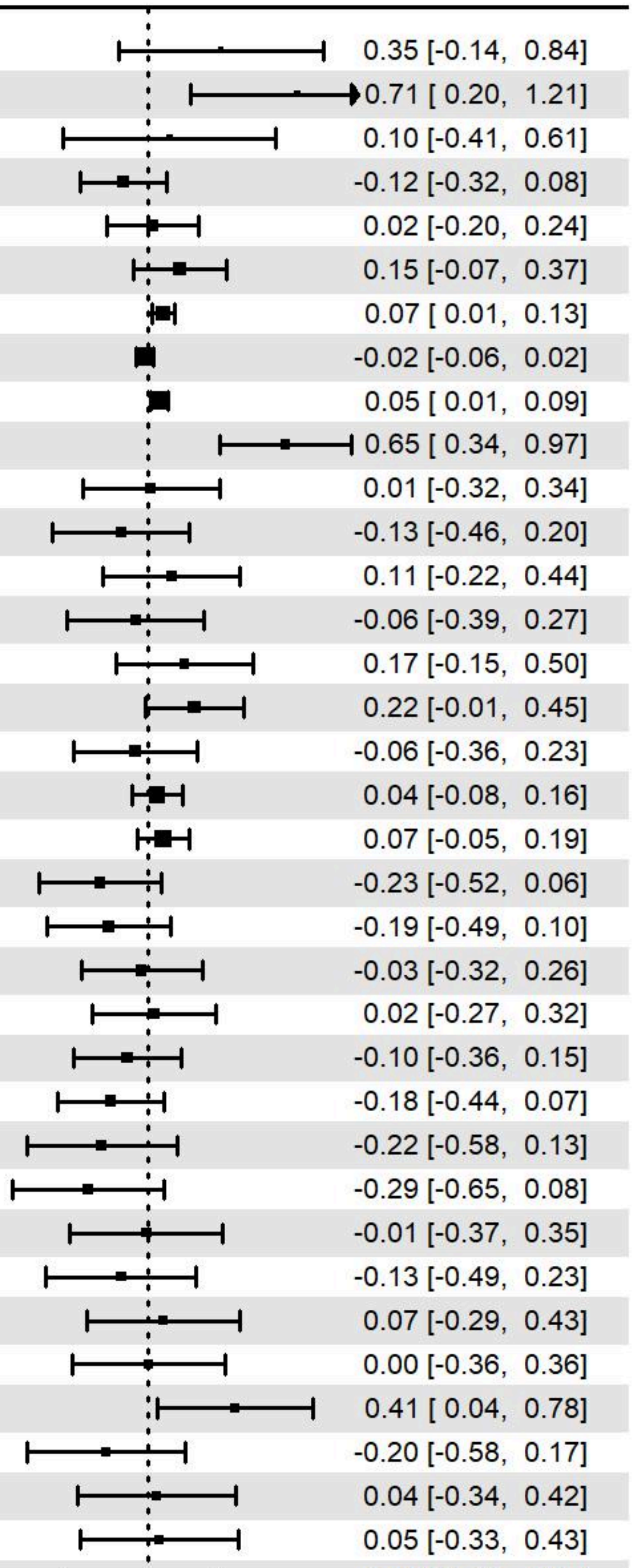

Reference	Estimate [95% CI]
Arthur et al., 2019	0.35 [-0.14, 0.84]
Arthur et al., 2019	0.71 [0.20, 1.21]
Arthur et al., 2019	0.10 [-0.41, 0.61]
Baumgartner & Schneider, 2023	-0.12 [-0.32, 0.08]
Behnke et al., 2020	0.02 [-0.20, 0.24]
Behnke et al., 2020	0.15 [-0.07, 0.37]
Behnke et al., 2022	0.07 [0.01, 0.13]
Behnke et al., 2024	-0.02 [-0.06, 0.02]
Behnke et al., 2024	0.05 [0.01, 0.09]
Brimmell et al., 2019	0.65 [0.34, 0.97]
Crowe et al., 2020	0.01 [-0.32, 0.34]
Crowe et al., 2020	-0.13 [-0.46, 0.20]
Crowe et al., 2020	0.11 [-0.22, 0.44]
Crowe et al., 2020	-0.06 [-0.39, 0.27]
Crowe et al., 2020	0.17 [-0.15, 0.50]
Gurera & Isaacowitz, 2022	0.22 [-0.01, 0.45]
Gurera & Isaacowitz, 2022	-0.06 [-0.36, 0.23]
Hangen et al., 2019	0.04 [-0.08, 0.16]
Hangen et al., 2019	0.07 [-0.05, 0.19]
Hase et al., 2019	-0.23 [-0.52, 0.06]
Hase et al., 2019	-0.19 [-0.49, 0.10]
Hase et al., 2019	-0.03 [-0.32, 0.26]
Hase et al., 2019	0.02 [-0.27, 0.32]
Hase et al., 2019	-0.10 [-0.36, 0.15]
Hase et al., 2019	-0.18 [-0.44, 0.07]
Hase et al., in preparation	-0.22 [-0.58, 0.13]
Hase et al., in preparation	-0.29 [-0.65, 0.08]
Hase et al., in preparation	-0.01 [-0.37, 0.35]
Hase et al., in preparation	-0.13 [-0.49, 0.23]
Hase et al., in preparation	0.07 [-0.29, 0.43]
Hase et al., in preparation	0.00 [-0.36, 0.36]
Hase et al., in preparation	0.41 [0.04, 0.78]
Hase et al., in preparation	-0.20 [-0.58, 0.17]
Hase et al., in preparation	0.04 [-0.34, 0.42]
Hase et al., in preparation	0.05 [-0.33, 0.43]

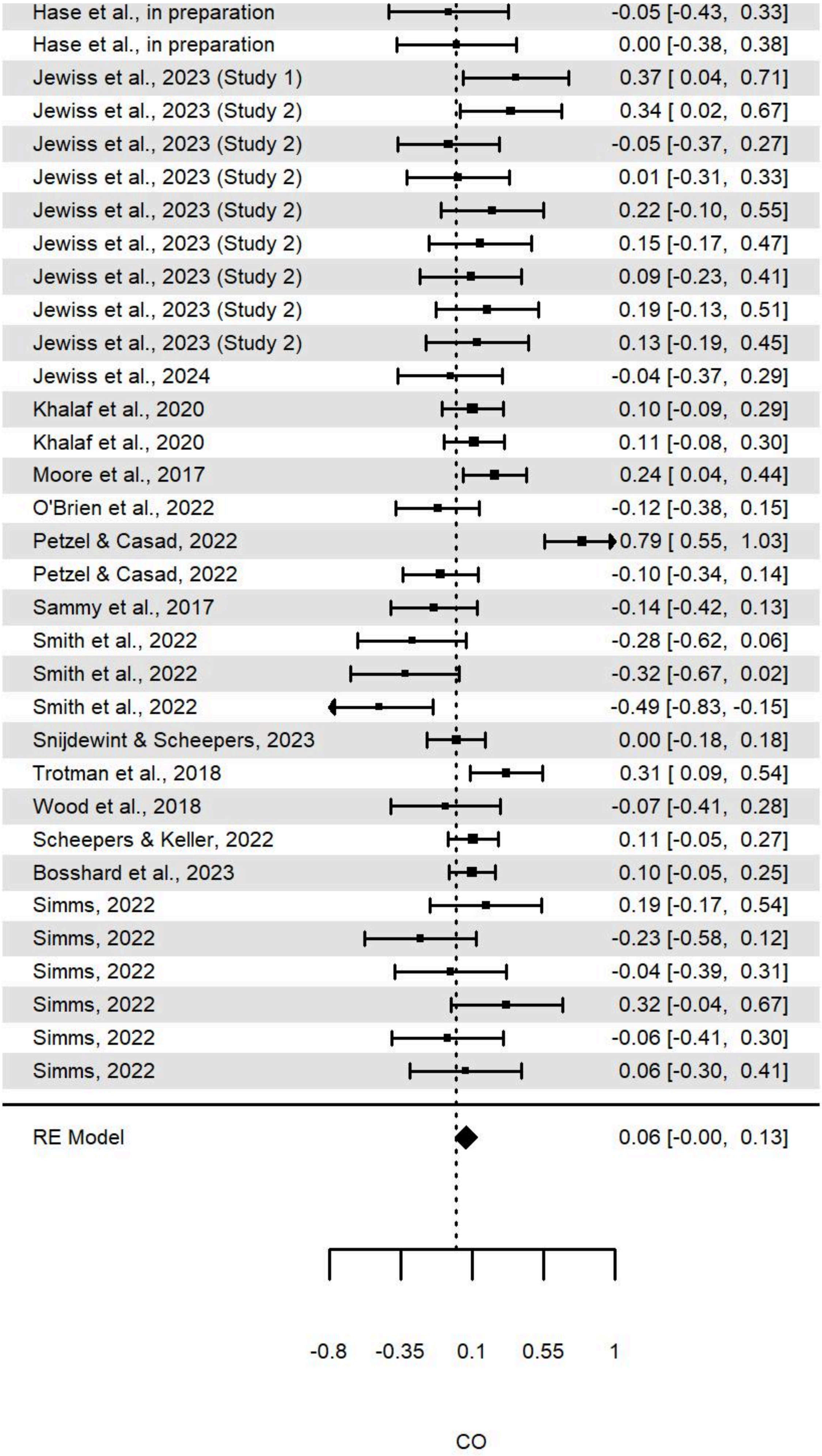

Hase et al., in preparation -0.05 [-0.43, 0.33]
Hase et al., in preparation 0.00 [-0.38, 0.38]
Jewiss et al., 2023 (Study 1) 0.37 [0.04, 0.71]
Jewiss et al., 2023 (Study 2) 0.34 [0.02, 0.67]
Jewiss et al., 2023 (Study 2) -0.05 [-0.37, 0.27]
Jewiss et al., 2023 (Study 2) 0.01 [-0.31, 0.33]
Jewiss et al., 2023 (Study 2) 0.22 [-0.10, 0.55]
Jewiss et al., 2023 (Study 2) 0.15 [-0.17, 0.47]
Jewiss et al., 2023 (Study 2) 0.09 [-0.23, 0.41]
Jewiss et al., 2023 (Study 2) 0.19 [-0.13, 0.51]
Jewiss et al., 2023 (Study 2) 0.13 [-0.19, 0.45]
Jewiss et al., 2024 -0.04 [-0.37, 0.29]
Khalaf et al., 2020 0.10 [-0.09, 0.29]
Khalaf et al., 2020 0.11 [-0.08, 0.30]
Moore et al., 2017 0.24 [0.04, 0.44]
O'Brien et al., 2022 -0.12 [-0.38, 0.15]
Petzel & Casad, 2022 0.79 [0.55, 1.03]
Petzel & Casad, 2022 -0.10 [-0.34, 0.14]
Sammy et al., 2017 -0.14 [-0.42, 0.13]
Smith et al., 2022 -0.28 [-0.62, 0.06]
Smith et al., 2022 -0.32 [-0.67, 0.02]
Smith et al., 2022 -0.49 [-0.83, -0.15]
Snijdewint & Scheepers, 2023 0.00 [-0.18, 0.18]
Trotman et al., 2018 0.31 [0.09, 0.54]
Wood et al., 2018 -0.07 [-0.41, 0.28]
Scheepers & Keller, 2022 0.11 [-0.05, 0.27]
Bosshard et al., 2023 0.10 [-0.05, 0.25]
Simms, 2022 0.19 [-0.17, 0.54]
Simms, 2022 -0.23 [-0.58, 0.12]
Simms, 2022 -0.04 [-0.39, 0.31]
Simms, 2022 0.32 [-0.04, 0.67]
Simms, 2022 -0.06 [-0.41, 0.30]
Simms, 2022 0.06 [-0.30, 0.41]
RE Model 0.06 [-0.00, 0.13]
-0.8
-0.35
0.1
0.55
1
CO

```
knitr::include_graphics("forest_plot_TPR.jpg")
```

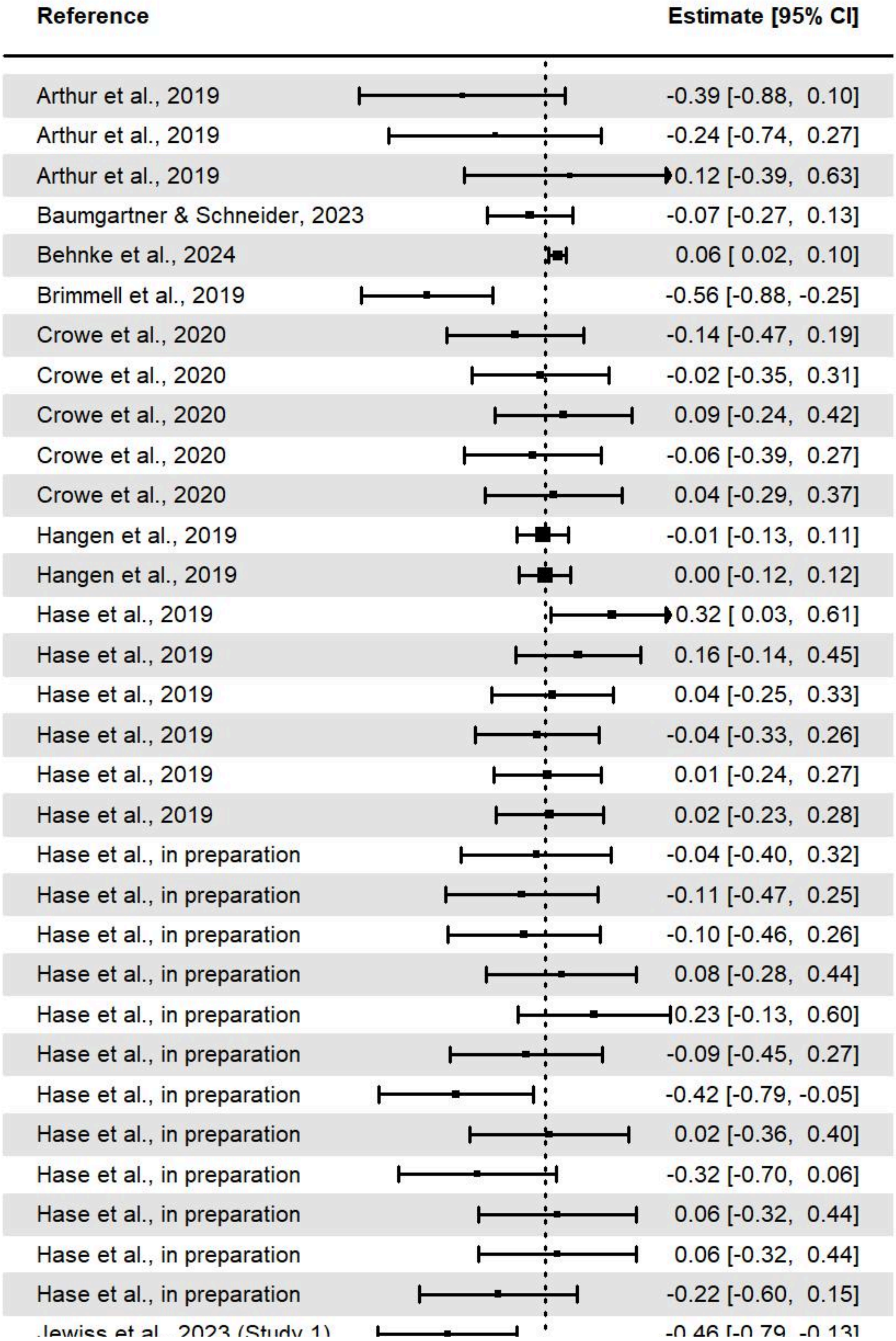

Reference
Estimate [95% CI]
Arthur et al., 2019 -0.39 [-0.88, 0.10]
Arthur et al., 2019 -0.24 [-0.74, 0.27]
Arthur et al., 2019 0.12 [-0.39, 0.63]
Baumgartner & Schneider, 2023 -0.07 [-0.27, 0.13]
Behnke et al., 2024 0.06 [0.02, 0.10]
Brimmell et al., 2019 -0.56 [-0.88, -0.25]
Crowe et al., 2020 -0.14 [-0.47, 0.19]
Crowe et al., 2020 -0.02 [-0.35, 0.31]
Crowe et al., 2020 0.09 [-0.24, 0.42]
Crowe et al., 2020 -0.06 [-0.39, 0.27]
Crowe et al., 2020 0.04 [-0.29, 0.37]
Hangen et al., 2019 -0.01 [-0.13, 0.11]
Hangen et al., 2019 0.00 [-0.12, 0.12]
Hase et al., 2019 0.32 [0.03, 0.61]
Hase et al., 2019 0.16 [-0.14, 0.45]
Hase et al., 2019 0.04 [-0.25, 0.33]
Hase et al., 2019 -0.04 [-0.33, 0.26]
Hase et al., 2019 0.01 [-0.24, 0.27]
Hase et al., 2019 0.02 [-0.23, 0.28]
Hase et al., in preparation -0.04 [-0.40, 0.32]
Hase et al., in preparation -0.11 [-0.47, 0.25]
Hase et al., in preparation -0.10 [-0.46, 0.26]
Hase et al., in preparation 0.08 [-0.28, 0.44]
Hase et al., in preparation 0.23 [-0.13, 0.60]
Hase et al., in preparation -0.09 [-0.45, 0.27]
Hase et al., in preparation -0.42 [-0.79, -0.05]
Hase et al., in preparation 0.02 [-0.36, 0.40]
Hase et al., in preparation -0.32 [-0.70, 0.06]
Hase et al., in preparation 0.06 [-0.32, 0.44]
Hase et al., in preparation 0.06 [-0.32, 0.44]
Hase et al., in preparation -0.22 [-0.60, 0.15]
Jewiss et al., 2023 (Study 1) -0.46 [-0.79, -0.13]

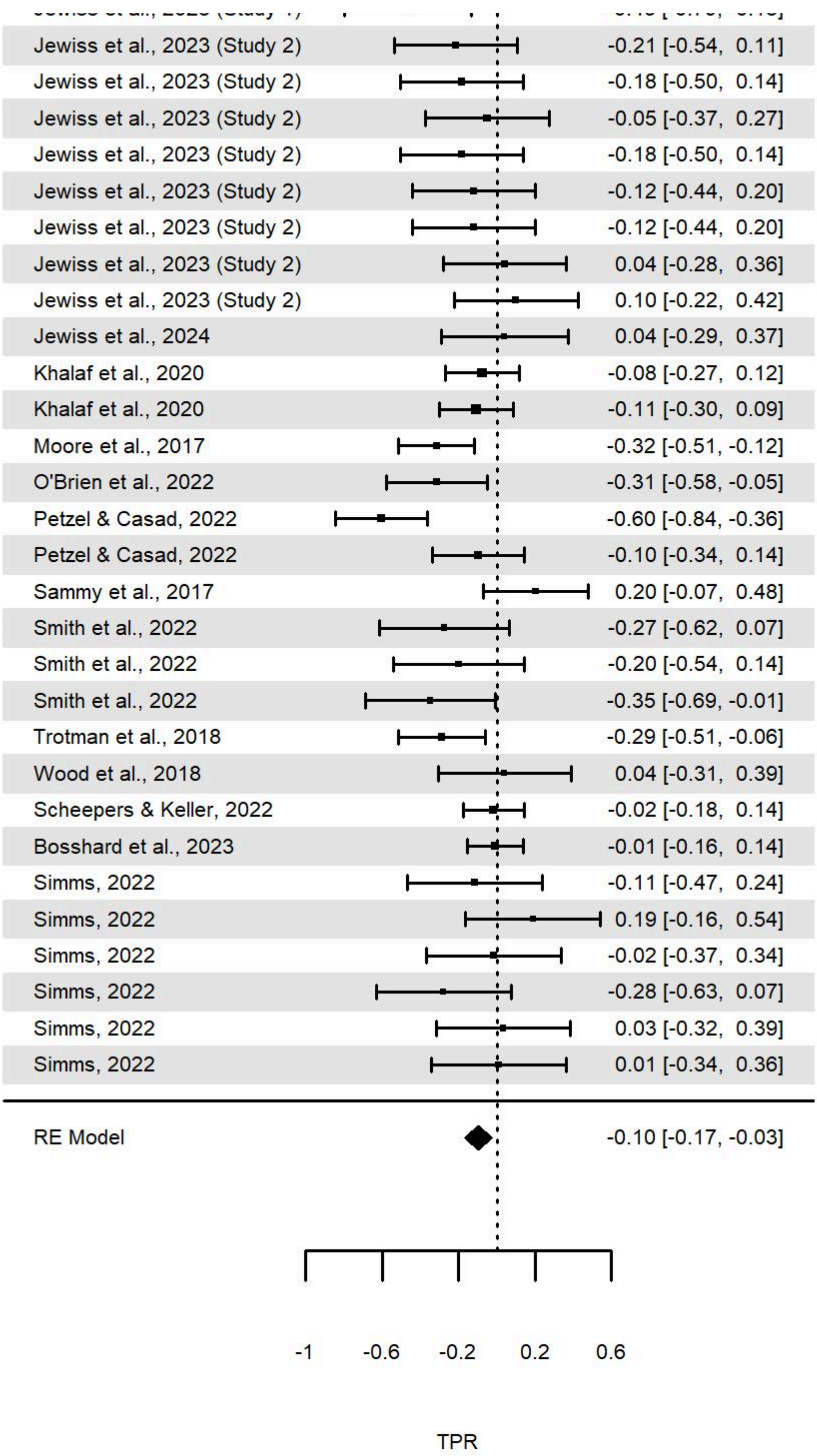
Jewiss et al., 2023 (Study 2) -0.21 [-0.54, 0.11]
Jewiss et al., 2023 (Study 2) -0.18 [-0.50, 0.14]
Jewiss et al., 2023 (Study 2) -0.05 [-0.37, 0.27]
Jewiss et al., 2023 (Study 2) -0.18 [-0.50, 0.14]
Jewiss et al., 2023 (Study 2) -0.12 [-0.44, 0.20]
Jewiss et al., 2023 (Study 2) -0.12 [-0.44, 0.20]
Jewiss et al., 2023 (Study 2) 0.04 [-0.28, 0.36]
Jewiss et al., 2023 (Study 2) 0.10 [-0.22, 0.42]
Jewiss et al., 2024 0.04 [-0.29, 0.37]
Khalaf et al., 2020 -0.08 [-0.27, 0.12]
Khalaf et al., 2020 -0.11 [-0.30, 0.09]
Moore et al., 2017 -0.32 [-0.51, -0.12]
O'Brien et al., 2022 -0.31 [-0.58, -0.05]
Petzel & Casad, 2022 -0.60 [-0.84, -0.36]
Petzel & Casad, 2022 -0.10 [-0.34, 0.14]
Sammy et al., 2017 0.20 [-0.07, 0.48]
Smith et al., 2022 -0.27 [-0.62, 0.07]
Smith et al., 2022 -0.20 [-0.54, 0.14]
Smith et al., 2022 -0.35 [-0.69, -0.01]
Trotman et al., 2018 -0.29 [-0.51, -0.06]
Wood et al., 2018 0.04 [-0.31, 0.39]
Scheepers & Keller, 2022 -0.02 [-0.18, 0.14]
Bosshard et al., 2023 -0.01 [-0.16, 0.14]
Simms, 2022 -0.11 [-0.47, 0.24]
Simms, 2022 0.19 [-0.16, 0.54]
Simms, 2022 -0.02 [-0.37, 0.34]
Simms, 2022 -0.28 [-0.63, 0.07]
Simms, 2022 0.03 [-0.32, 0.39]
Simms, 2022 0.01 [-0.34, 0.36]
RE Model -0.10 [-0.17, -0.03]
-1
-0.6
-0.2
0.2
0.6
TPR

```
knitr::include_graphics("forest_plot_CTI.jpg")
```

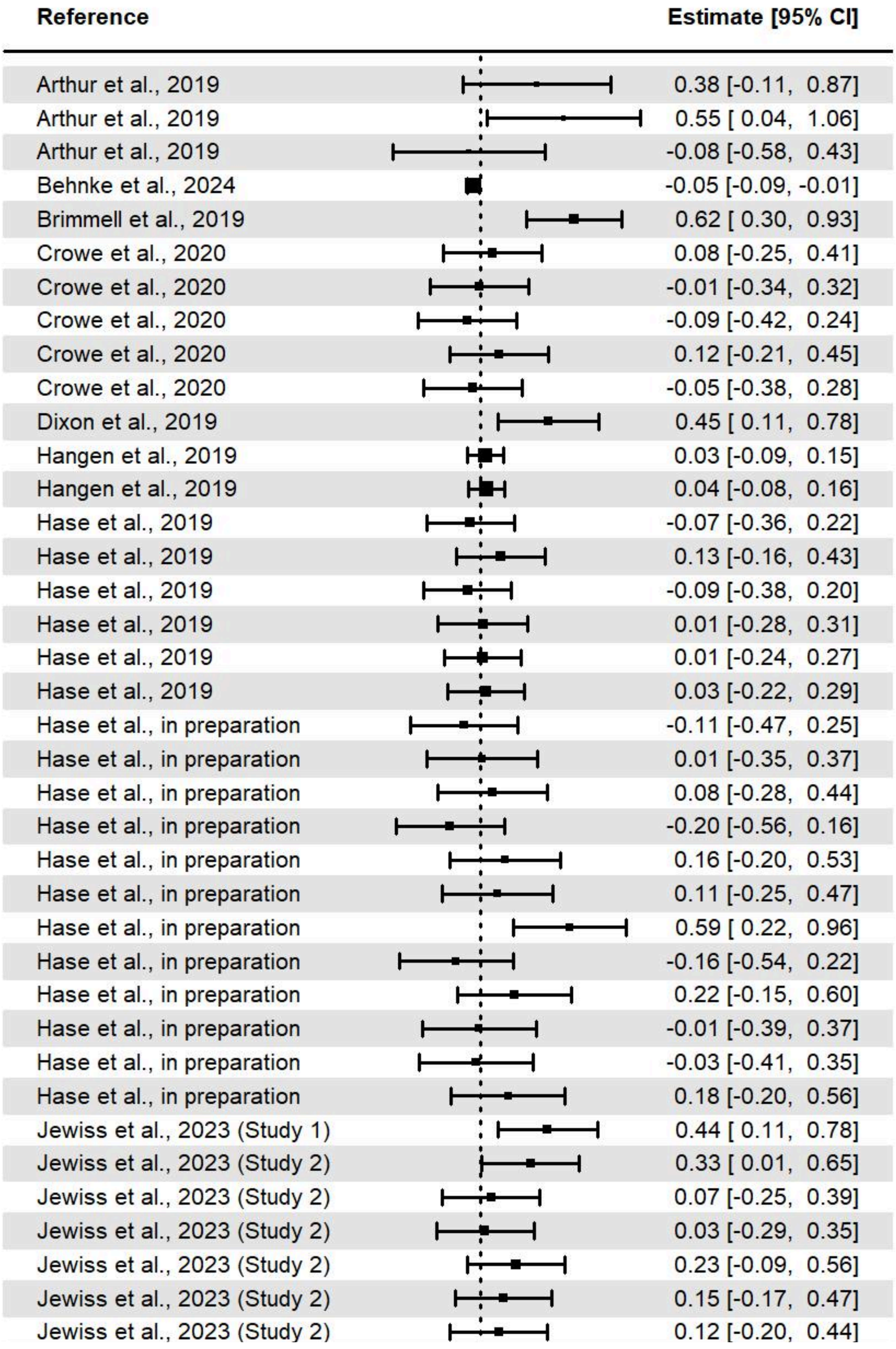
Reference
Estimate [95% CI]
Arthur et al., 2019 0.38 [-0.11, 0.87]
Arthur et al., 2019 0.55 [0.04, 1.06]
Arthur et al., 2019 -0.08 [-0.58, 0.43]
Behnke et al., 2024 -0.05 [-0.09, -0.01]
Brimmell et al., 2019 0.62 [0.30, 0.93]
Crowe et al., 2020 0.08 [-0.25, 0.41]
Crowe et al., 2020 -0.01 [-0.34, 0.32]
Crowe et al., 2020 -0.09 [-0.42, 0.24]
Crowe et al., 2020 0.12 [-0.21, 0.45]
Crowe et al., 2020 -0.05 [-0.38, 0.28]
Dixon et al., 2019 0.45 [0.11, 0.78]
Hangen et al., 2019 0.03 [-0.09, 0.15]
Hangen et al., 2019 0.04 [-0.08, 0.16]
Hase et al., 2019 -0.07 [-0.36, 0.22]
Hase et al., 2019 0.13 [-0.16, 0.43]
Hase et al., 2019 -0.09 [-0.38, 0.20]
Hase et al., 2019 0.01 [-0.28, 0.31]
Hase et al., 2019 0.01 [-0.24, 0.27]
Hase et al., 2019 0.03 [-0.22, 0.29]
Hase et al., in preparation -0.11 [-0.47, 0.25]
Hase et al., in preparation 0.01 [-0.35, 0.37]
Hase et al., in preparation 0.08 [-0.28, 0.44]
Hase et al., in preparation -0.20 [-0.56, 0.16]
Hase et al., in preparation 0.16 [-0.20, 0.53]
Hase et al., in preparation 0.11 [-0.25, 0.47]
Hase et al., in preparation 0.59 [0.22, 0.96]
Hase et al., in preparation -0.16 [-0.54, 0.22]
Hase et al., in preparation 0.22 [-0.15, 0.60]
Hase et al., in preparation -0.01 [-0.39, 0.37]
Hase et al., in preparation -0.03 [-0.41, 0.35]
Hase et al., in preparation 0.18 [-0.20, 0.56]
Jewiss et al., 2023 (Study 1) 0.44 [0.11, 0.78]
Jewiss et al., 2023 (Study 2) 0.33 [0.01, 0.65]
Jewiss et al., 2023 (Study 2) 0.07 [-0.25, 0.39]
Jewiss et al., 2023 (Study 2) 0.03 [-0.29, 0.35]
Jewiss et al., 2023 (Study 2) 0.23 [-0.09, 0.56]
Jewiss et al., 2023 (Study 2) 0.15 [-0.17, 0.47]
Jewiss et al., 2023 (Study 2) 0.12 [-0.20, 0.44]

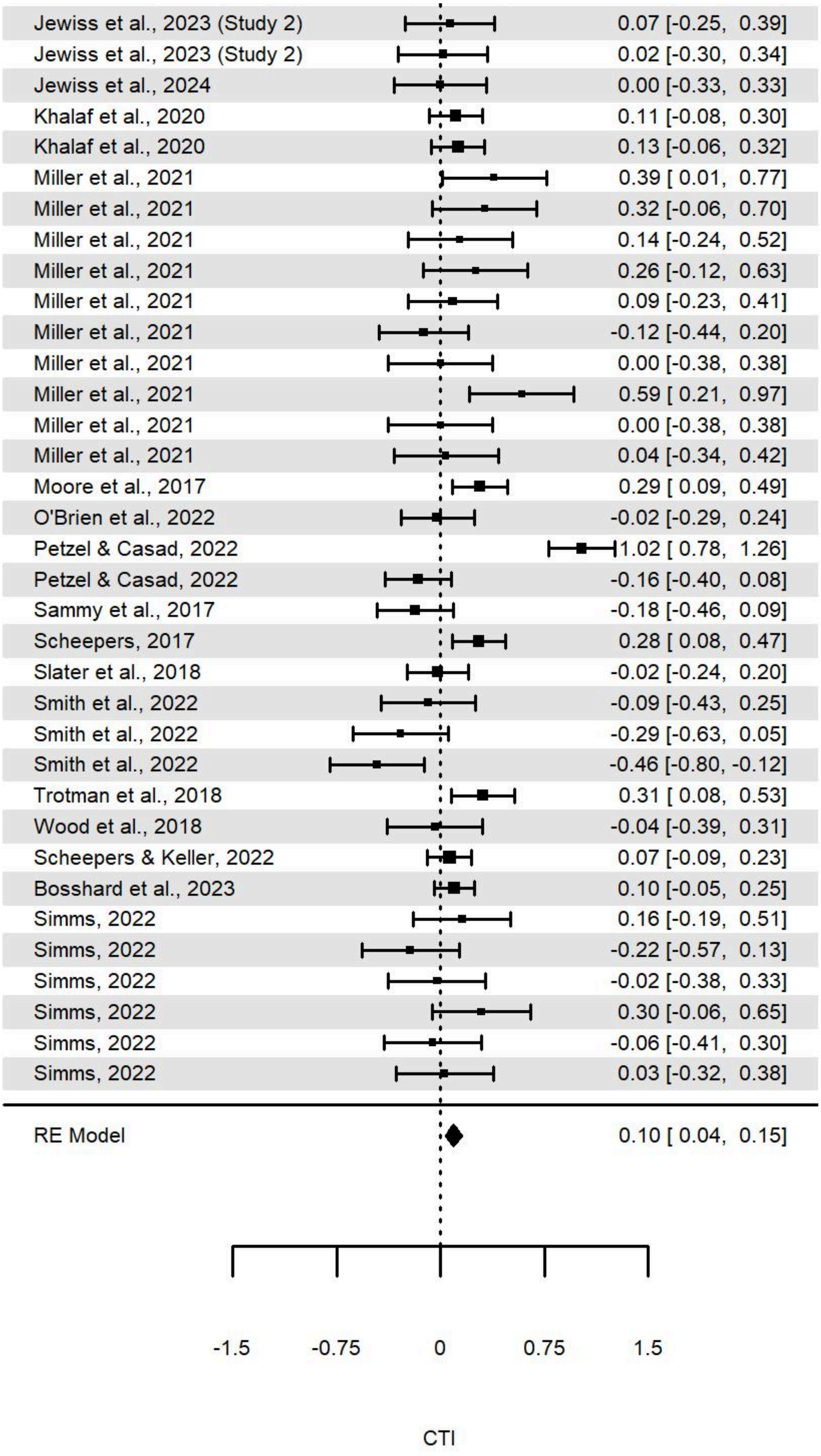
Jewiss et al., 2023 (Study 2) 0.07 [-0.25, 0.39]
Jewiss et al., 2023 (Study 2) 0.02 [-0.30, 0.34]
Jewiss et al., 2024 0.00 [-0.33, 0.33]
Khalaf et al., 2020 0.11 [-0.08, 0.30]
Khalaf et al., 2020 0.13 [-0.06, 0.32]
Miller et al., 2021 0.39 [0.01, 0.77]
Miller et al., 2021 0.32 [-0.06, 0.70]
Miller et al., 2021 0.14 [-0.24, 0.52]
Miller et al., 2021 0.26 [-0.12, 0.63]
Miller et al., 2021 0.09 [-0.23, 0.41]
Miller et al., 2021 -0.12 [-0.44, 0.20]
Miller et al., 2021 0.00 [-0.38, 0.38]
Miller et al., 2021 0.59 [0.21, 0.97]
Miller et al., 2021 0.00 [-0.38, 0.38]
Miller et al., 2021 0.04 [-0.34, 0.42]
Moore et al., 2017 0.29 [0.09, 0.49]
O'Brien et al., 2022 -0.02 [-0.29, 0.24]
Petzel & Casad, 2022 1.02 [0.78, 1.26]
Petzel & Casad, 2022 -0.16 [-0.40, 0.08]
Sammy et al., 2017 -0.18 [-0.46, 0.09]
Scheepers, 2017 0.28 [0.08, 0.47]
Slater et al., 2018 -0.02 [-0.24, 0.20]
Smith et al., 2022 -0.09 [-0.43, 0.25]
Smith et al., 2022 -0.29 [-0.63, 0.05]
Smith et al., 2022 -0.46 [-0.80, -0.12]
Trotman et al., 2018 0.31 [0.08, 0.53]
Wood et al., 2018 -0.04 [-0.39, 0.31]
Scheepers & Keller, 2022 0.07 [-0.09, 0.23]
Bosshard et al., 2023 0.10 [-0.05, 0.25]
Simms, 2022 0.16 [-0.19, 0.51]
Simms, 2022 -0.22 [-0.57, 0.13]
Simms, 2022 -0.02 [-0.38, 0.33]
Simms, 2022 0.30 [-0.06, 0.65]
Simms, 2022 -0.06 [-0.41, 0.30]
Simms, 2022 0.03 [-0.32, 0.38]
RE Model 0.10 [0.04, 0.15]
-1.5
-0.75
0
0.75
1.5
CTI

```
knitr::include_graphics("forest_plot_Cogni.jpg")
```

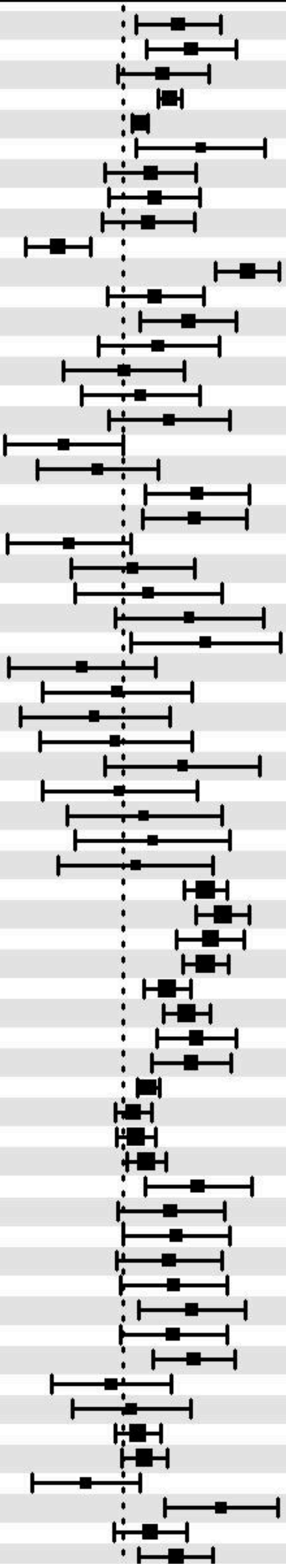

Reference	Estimate [95% CI]
Baumgartner & Schneider, 2023	0.27 [0.06, 0.47]
Behnke et al., 2020	0.33 [0.11, 0.55]
Behnke et al., 2020	0.19 [-0.03, 0.41]
Behnke et al., 2022	0.22 [0.17, 0.28]
Behnke et al., 2024	0.08 [0.04, 0.12]
Brimmell et al., 2019	0.38 [0.06, 0.69]
Cabral et al., 2024	0.13 [-0.09, 0.35]
Cabral et al., 2024	0.15 [-0.07, 0.37]
Cabral et al., 2024	0.12 [-0.10, 0.34]
Carenzo et al., 2020	-0.32 [-0.48, -0.16]
Carenzo et al., 2020	0.60 [0.45, 0.76]
Gurera & Isaacowitz, 2022	0.15 [-0.08, 0.39]
Gurera & Isaacowitz, 2022	0.32 [0.08, 0.55]
Gurera & Isaacowitz, 2022	0.17 [-0.13, 0.47]
Gurera & Isaacowitz, 2022	0.00 [-0.29, 0.30]
Hase et al., 2019	0.08 [-0.21, 0.37]
Hase et al., 2019	0.22 [-0.07, 0.52]
Hase et al., 2019	-0.29 [-0.58, -0.00]
Hase et al., 2019	-0.13 [-0.42, 0.17]
Hase et al., 2019	0.36 [0.10, 0.61]
Hase et al., 2019	0.35 [0.09, 0.60]
Hase et al., in preparation	-0.27 [-0.57, 0.03]
Hase et al., in preparation	0.04 [-0.25, 0.34]
Hase et al., in preparation	0.12 [-0.24, 0.48]
Hase et al., in preparation	0.32 [-0.04, 0.68]
Hase et al., in preparation	0.40 [0.04, 0.76]
Hase et al., in preparation	-0.20 [-0.56, 0.16]
Hase et al., in preparation	-0.03 [-0.39, 0.33]
Hase et al., in preparation	-0.14 [-0.50, 0.22]
Hase et al., in preparation	-0.04 [-0.41, 0.33]
Hase et al., in preparation	0.29 [-0.09, 0.66]
Hase et al., in preparation	-0.02 [-0.40, 0.36]
Hase et al., in preparation	0.10 [-0.28, 0.48]
Hase et al., in preparation	0.14 [-0.24, 0.52]
Hase et al., in preparation	0.06 [-0.32, 0.44]
Jamieson et al., 2022	0.40 [0.29, 0.51]
Jamieson et al., 2022	0.48 [0.35, 0.62]
Jamieson et al., 2022	0.42 [0.26, 0.59]
Journault et al., in preparation	0.40 [0.29, 0.51]
Journault et al., in preparation	0.21 [0.10, 0.33]
Journault et al., in preparation	0.31 [0.20, 0.42]
Khalaf et al., 2020	0.35 [0.16, 0.55]
Khalaf et al., 2020	0.33 [0.14, 0.52]
Laurin & Pellet, 2023	0.12 [0.07, 0.17]
Lee et al., 2019	0.05 [-0.04, 0.14]
Malkoc et al., 2023	0.06 [-0.03, 0.16]
Malkoc et al., 2024	0.11 [0.01, 0.20]
Moe & Putwain, 2020	0.36 [0.10, 0.62]
Moe & Putwain, 2020	0.23 [-0.03, 0.49]
Moe & Putwain, 2020	0.26 [-0.00, 0.52]
Moe & Putwain, 2020	0.22 [-0.04, 0.48]
Moe & Putwain, 2020	0.24 [-0.01, 0.50]
Moe & Putwain, 2020	0.33 [0.07, 0.59]
Moe & Putwain, 2020	0.24 [-0.02, 0.50]
Moore et al., 2017	0.34 [0.14, 0.54]
Mosley et al., 2018	-0.06 [-0.35, 0.23]
Mosley et al., 2018	0.04 [-0.25, 0.33]
Mulvenna et al., 2023	0.07 [-0.04, 0.18]
Mulvenna et al., 2023	0.10 [-0.01, 0.21]
O'Brien et al., 2022	-0.18 [-0.45, 0.08]
Sammy et al., 2017	0.47 [0.20, 0.75]
Schickel et al., 2023	0.13 [-0.05, 0.31]
Schickel et al., 2023	0.26 [0.08, 0.43]

11 Moderator Analysis

11.1 Moderator Analysis - main code

```
model_CO_multilevel_BD <- rma.mv(yi = CO_cor_Z, V = CO_cor_Z_var, slab = Ref_APA, data
= Data, random = ~ 1 | paper_id/effect_size_id, test = "t", method = "REML", mods = ~ f
actor(Cardio_before0_during1)-1, tdist=TRUE)

## Warning: 94 rows with NAs omitted from model fitting.

model_CO_multilevel_Age <- rma.mv(yi = CO_cor_Z, V = CO_cor_Z_var, slab = Ref_APA, data
= Data, random = ~ 1 | paper_id/effect_size_id, test = "t", method = "REML", mods = ~ a
ge, tdist=TRUE)

## Warning: 94 rows with NAs omitted from model fitting.

model_CO_multilevel_Sex <- rma.mv(yi = CO_cor_Z, V = CO_cor_Z_var, slab = Ref_APA, data
= Data, random = ~ 1 | paper_id/effect_size_id, test = "t", method = "REML", mods = ~ s
ex_percent_F, tdist=TRUE)

## Warning: 94 rows with NAs omitted from model fitting.

model_CO_multilevel_Rob <- rma.mv(yi = CO_cor_Z, V = CO_cor_Z_var, slab = Ref_APA, data
= Data, random = ~ 1 | paper_id/effect_size_id, test = "t", method = "REML", mods = ~ f
actor(overall_RoB)-1, tdist=TRUE)

## Warning: 95 rows with NAs omitted from model fitting.

## Warning: Redundant predictors dropped from the model.

model_CO_multilevel_BD
```

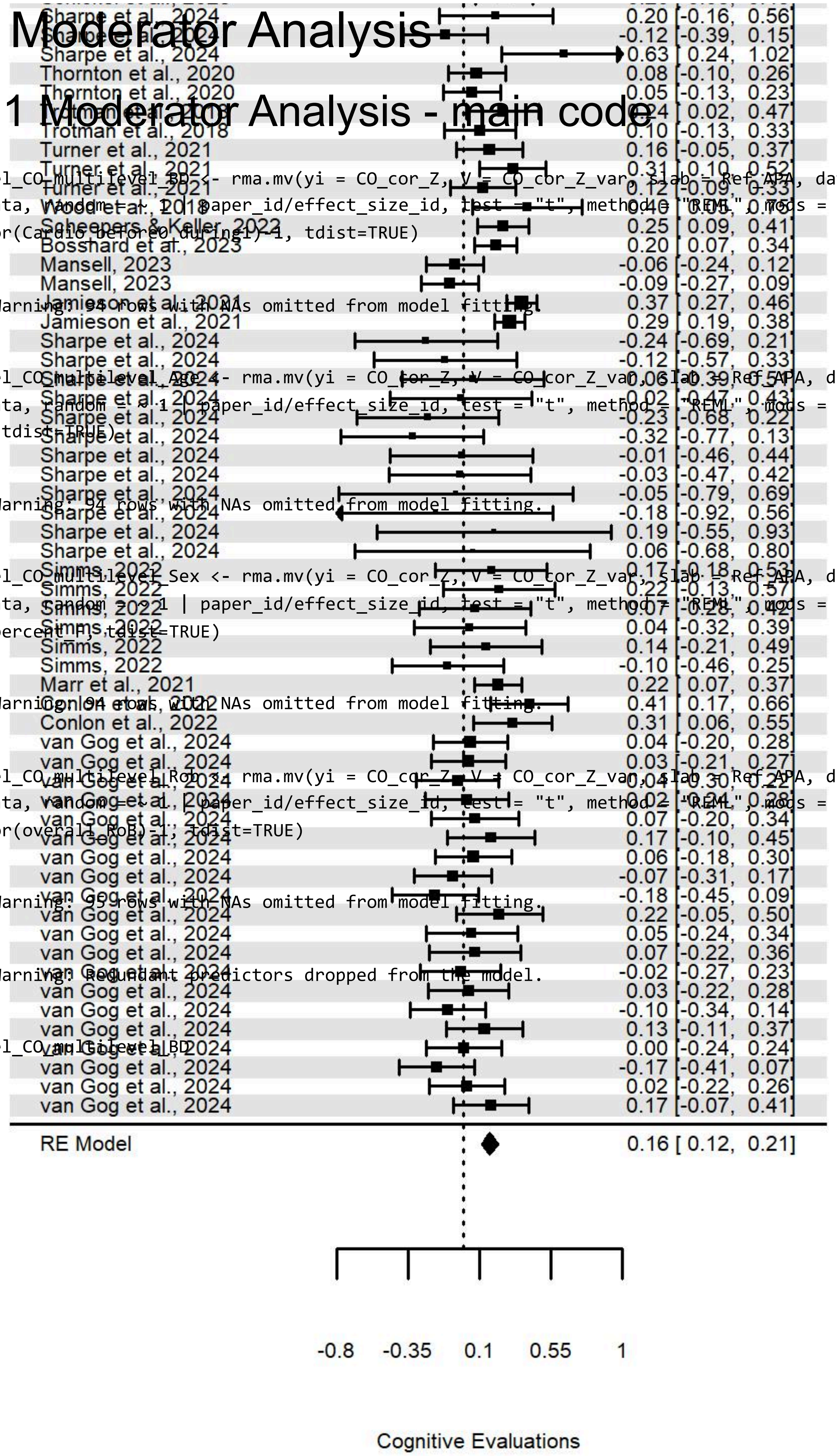

```
##
## Multivariate Meta-Analysis Model (k = 68; method: REML)
##
## Variance Components:
##
##             estim    sqrt  nlvls  fixed                  factor
## sigma^2.1  0.0127  0.1127     28     no                paper_id
## sigma^2.2  0.0121  0.1099     68     no  paper_id/effect_size_id
##
## Test for Residual Heterogeneity:
## QE(df = 66) = 143.8691, p-val < .0001
##
## Test of Moderators (coefficients 1:2):
## F(df1 = 2, df2 = 66) = 2.0653, p-val = 0.1349
##
## Model Results:
##
##                                  estimate      se    tval  df    pval    ci.lb
## factor(Cardio_before0_during1)0    0.0439  0.0385  1.1396  66  0.2586  -0.0330
## factor(Cardio_before0_during1)1    0.0925  0.0520  1.7786  66  0.0799  -0.0113
##                                   ci.ub
## factor(Cardio_before0_during1)0  0.1209
## factor(Cardio_before0_during1)1  0.1963  .
##
## ---
## Signif. codes:  0 '***' 0.001 '**' 0.01 '*' 0.05 '.' 0.1 ' ' 1
```

```
model_CO_multilevel_Age
```

```
##
## Multivariate Meta-Analysis Model (k = 68; method: REML)
##
## Variance Components:
##
##             estim    sqrt  nlvls  fixed                  factor
## sigma^2.1  0.0149  0.1219     28     no                paper_id
## sigma^2.2  0.0105  0.1022     68     no  paper_id/effect_size_id
##
## Test for Residual Heterogeneity:
## QE(df = 66) = 150.6689, p-val < .0001
##
## Test of Moderators (coefficient 2):
## F(df1 = 1, df2 = 66) = 1.1833, p-val = 0.2806
##
## Model Results:
##
##          estimate      se     tval  df    pval    ci.lb   ci.ub
## intrcpt    0.1632  0.0987   1.6524  66  0.1032  -0.0340  0.3603
## age       -0.0043  0.0040  -1.0878  66  0.2806  -0.0123  0.0036
##
## ---
## Signif. codes:  0 '***' 0.001 '**' 0.01 '*' 0.05 '.' 0.1 ' ' 1
```

```
model_CO_multilevel_Sex
```

```
##
## Multivariate Meta-Analysis Model (k = 68; method: REML)
##
## Variance Components:
##
##             estim    sqrt  nlvls  fixed                  factor
## sigma^2.1  0.0147  0.1213     28     no                paper_id
## sigma^2.2  0.0115  0.1074     68     no  paper_id/effect_size_id
##
## Test for Residual Heterogeneity:
## QE(df = 66) = 148.0255, p-val < .0001
##
## Test of Moderators (coefficient 2):
## F(df1 = 1, df2 = 66) = 0.0336, p-val = 0.8552
##
## Model Results:
##
##                estimate      se    tval  df    pval    ci.lb   ci.ub
## intrcpt          0.0536  0.0552  0.9702  66  0.3355  -0.0567  0.1638
## sex_percent_F    0.0230  0.1257  0.1833  66  0.8552  -0.2280  0.2740
##
## ---
## Signif. codes:  0 '***' 0.001 '**' 0.01 '*' 0.05 '.' 0.1 ' ' 1
```

```
model_CO_multilevel_Rob
```

```
##
## Multivariate Meta-Analysis Model (k = 67; method: REML)
##
## Variance Components:
##
##             estim    sqrt  nlvls  fixed                  factor
## sigma^2.1  0.0163  0.1277     27     no                paper_id
## sigma^2.2  0.0118  0.1087     67     no  paper_id/effect_size_id
##
## Test for Residual Heterogeneity:
## QE(df = 65) = 150.0373, p-val < .0001
##
## Test of Moderators (coefficients 1:2):
## F(df1 = 2, df2 = 65) = 1.5413, p-val = 0.2218
##
## Model Results:
##
##                       estimate      se    tval  df    pval    ci.lb   ci.ub
## factor(overall_RoB)1    0.0645  0.0395  1.6334  65  0.1072  -0.0144  0.1433
## factor(overall_RoB)2    0.0485  0.0753  0.6439  65  0.5219  -0.1019  0.1988
##
## ---
## Signif. codes:  0 '***' 0.001 '**' 0.01 '*' 0.05 '.' 0.1 ' ' 1
```

```
model_TPR_multilevel_BD <- rma.mv(yi = TPR_cor_Z, V = TPR_cor_Z_var, slab = Ref_APA, da
ta = Data, random = ~ 1 | paper_id/effect_size_id, test = "t", method = "REML", mods =
~ factor(Cardio_before0_during1)-1, tdist=TRUE)
```

```
## Warning: 101 rows with NAs omitted from model fitting.
```

```
model_TPR_multilevel_Age <- rma.mv(yi = TPR_cor_Z, V = TPR_cor_Z_var, slab = Ref_APA, d
ata = Data, random = ~ 1 | paper_id/effect_size_id, test = "t", method = "REML", mods =
~ age, tdist=TRUE)
```

```
## Warning: 101 rows with NAs omitted from model fitting.
```

```
model_TPR_multilevel_Sex <- rma.mv(yi = TPR_cor_Z, V = TPR_cor_Z_var, slab = Ref_APA, d
ata = Data, random = ~ 1 | paper_id/effect_size_id, test = "t", method = "REML", mods =
~ sex_percent_F, tdist=TRUE)
```

```
## Warning: 101 rows with NAs omitted from model fitting.
```

```
model_TPR_multilevel_Rob <- rma.mv(yi = TPR_cor_Z, V = TPR_cor_Z_var, slab = Ref_APA, d
ata = Data, random = ~ 1 | paper_id/effect_size_id, test = "t", method = "REML", mods =
~ factor(overall_RoB)-1, tdist=TRUE)
```

```
## Warning: 102 rows with NAs omitted from model fitting.
## Warning: Redundant predictors dropped from the model.
```

```
model_TPR_multilevel_BD
```

```
##
## Multivariate Meta-Analysis Model (k = 61; method: REML)
##
## Variance Components:
##
##             estim    sqrt  nlvls  fixed                 factor
## sigma^2.1  0.0187  0.1369     24     no               paper_id
## sigma^2.2  0.0000  0.0000     61     no  paper_id/effect_size_id
##
## Test for Residual Heterogeneity:
## QE(df = 59) = 112.3671, p-val < .0001
##
## Test of Moderators (coefficients 1:2):
## F(df1 = 2, df2 = 59) = 3.9994, p-val = 0.0235
##
## Model Results:
##
##                                  estimate      se     tval  df    pval    ci.lb
## factor(Cardio_before0_during1)0   -0.0996  0.0397  -2.5082  59  0.0149  -0.1790
## factor(Cardio_before0_during1)1   -0.0971  0.0642  -1.5136  59  0.1355  -0.2255
##                                    ci.ub
## factor(Cardio_before0_during1)0  -0.0201  *
## factor(Cardio_before0_during1)1   0.0313
##
## ---
## Signif. codes:  0 '***' 0.001 '**' 0.01 '*' 0.05 '.' 0.1 ' ' 1
```

```
model_TPR_multilevel_Age
```

```
##
## Multivariate Meta-Analysis Model (k = 61; method: REML)
##
## Variance Components:
##
##             estim    sqrt  nlvls  fixed                 factor
## sigma^2.1  0.0188  0.1372     24     no               paper_id
## sigma^2.2  0.0000  0.0000     61     no  paper_id/effect_size_id
##
## Test for Residual Heterogeneity:
## QE(df = 59) = 116.3318, p-val < .0001
##
## Test of Moderators (coefficient 2):
## F(df1 = 1, df2 = 59) = 0.0791, p-val = 0.7796
##
## Model Results:
##
##          estimate      se     tval  df    pval    ci.lb   ci.ub
## intrcpt   -0.0108  0.3157  -0.0342  59  0.9728  -0.6424  0.6208
## age       -0.0039  0.0137  -0.2812  59  0.7796  -0.0313  0.0236
##
## ---
## Signif. codes:  0 '***' 0.001 '**' 0.01 '*' 0.05 '.' 0.1 ' ' 1
```

```
model_TPR_multilevel_Sex
```

```
##
## Multivariate Meta-Analysis Model (k = 61; method: REML)
##
## Variance Components:
##
##              estim    sqrt  nlvls  fixed                   factor
## sigma^2.1  0.0190  0.1378     24     no                 paper_id
## sigma^2.2  0.0000  0.0000     61     no  paper_id/effect_size_id
##
## Test for Residual Heterogeneity:
## QE(df = 59) = 104.7456, p-val = 0.0002
##
## Test of Moderators (coefficient 2):
## F(df1 = 1, df2 = 59) = 0.0089, p-val = 0.9253
##
## Model Results:
##
##                 estimate      se     tval  df    pval    ci.lb   ci.ub
## intrcpt          -0.0944  0.0612  -1.5421  59  0.1284  -0.2169  0.0281
## sex_percent_F    -0.0125  0.1326  -0.0942  59  0.9253  -0.2777  0.2528
##
## ---
## Signif. codes:  0 '***' 0.001 '**' 0.01 '*' 0.05 '.' 0.1 ' ' 1
```

```
model_TPR_multilevel_Rob
```

```
##
## Multivariate Meta-Analysis Model (k = 60; method: REML)
##
## Variance Components:
##
##              estim    sqrt  nlvls  fixed                  factor
## sigma^2.1  0.0098  0.0989     23     no                paper_id
## sigma^2.2  0.0000  0.0000     60     no  paper_id/effect_size_id
##
## Test for Residual Heterogeneity:
## QE(df = 58) = 88.9257, p-val = 0.0056
##
## Test of Moderators (coefficients 1:2):
## F(df1 = 2, df2 = 58) = 9.2274, p-val = 0.0003
##
## Model Results:
##
##                        estimate      se     tval  df    pval    ci.lb    ci.ub
## factor(overall_RoB)1   -0.0511  0.0329  -1.5528  58  0.1259  -0.1169   0.0148
## factor(overall_RoB)2   -0.2504  0.0625  -4.0054  58  0.0002  -0.3755  -0.1253
##
## factor(overall_RoB)1
## factor(overall_RoB)2  ***
##
## ---
## Signif. codes:  0 '***' 0.001 '**' 0.01 '*' 0.05 '.' 0.1 ' ' 1
```

```
model_CTI_multilevel_BD <- rma.mv(yi = CTI_cor_Z, V = CTI_cor_Z_var, slab = Ref_APA, da
ta = Data, random = ~ 1 | paper_id/effect_size_id, test = "t", method = "REML", mods =
~ factor(Cardio_before0_during1)-1, tdist=TRUE)
```

```
## Warning: 89 rows with NAs omitted from model fitting.
```

```
model_CTI_multilevel_Age <- rma.mv(yi = CTI_cor_Z, V = CTI_cor_Z_var, slab = Ref_APA, d
ata = Data, random = ~ 1 | paper_id/effect_size_id, test = "t", method = "REML", mods =
~ age, tdist=TRUE)
```

```
## Warning: 89 rows with NAs omitted from model fitting.
```

```
model_CTI_multilevel_Sex <- rma.mv(yi = CTI_cor_Z, V = CTI_cor_Z_var, slab = Ref_APA, d
ata = Data, random = ~ 1 | paper_id/effect_size_id, test = "t", method = "REML", mods =
~ sex_percent_F, tdist=TRUE)
```

```
## Warning: 89 rows with NAs omitted from model fitting.
```

```
model_CTI_multilevel_Rob <- rma.mv(yi = CTI_cor_Z, V = CTI_cor_Z_var, slab = Ref_APA, d
ata = Data, random = ~ 1 | paper_id/effect_size_id, test = "t", method = "REML", mods =
~ factor(overall_RoB)-1, tdist=TRUE)
```

```
## Warning: 90 rows with NAs omitted from model fitting.
## Warning: Redundant predictors dropped from the model.
```

```
model_CTI_multilevel_BD
```

```
##
## Multivariate Meta-Analysis Model (k = 73; method: REML)
##
## Variance Components:
##
##              estim    sqrt  nlvls  fixed                   factor
## sigma^2.1  0.0000  0.0000     28     no                 paper_id
## sigma^2.2  0.0290  0.1702     73     no  paper_id/effect_size_id
##
## Test for Residual Heterogeneity:
## QE(df = 71) = 181.6539, p-val < .0001
##
## Test of Moderators (coefficients 1:2):
## F(df1 = 2, df2 = 71) = 6.9192, p-val = 0.0018
##
## Model Results:
##
##                                   estimate      se    tval  df    pval   ci.lb
## factor(Cardio_before0_during1)0    0.0828  0.0300  2.7652  71  0.0072  0.0231
## factor(Cardio_before0_during1)1    0.1607  0.0646  2.4883  71  0.0152  0.0319
##                                    ci.ub
## factor(Cardio_before0_during1)0  0.1426  **
## factor(Cardio_before0_during1)1  0.2895   *
##
## ---
## Signif. codes:  0 '***' 0.001 '**' 0.01 '*' 0.05 '.' 0.1 ' ' 1
```

```
model_CTI_multilevel_Age
```

```
##
## Multivariate Meta-Analysis Model (k = 73; method: REML)
##
## Variance Components:
##
##             estim    sqrt  nlvls  fixed                 factor
## sigma^2.1  0.0018  0.0429     28     no               paper_id
## sigma^2.2  0.0286  0.1690     73     no  paper_id/effect_size_id
##
## Test for Residual Heterogeneity:
## QE(df = 71) = 195.3210, p-val < .0001
##
## Test of Moderators (coefficient 2):
## F(df1 = 1, df2 = 71) = 0.0196, p-val = 0.8891
##
## Model Results:
##
##          estimate      se    tval  df    pval    ci.lb   ci.ub
## intrcpt    0.0417  0.4099  0.1016  71  0.9193  -0.7757  0.8590
## age        0.0026  0.0184  0.1399  71  0.8891  -0.0341  0.0392
##
## ---
## Signif. codes:  0 '***' 0.001 '**' 0.01 '*' 0.05 '.' 0.1 ' ' 1
```

```
model_CTI_multilevel_Sex
```

```
##
## Multivariate Meta-Analysis Model (k = 73; method: REML)
##
## Variance Components:
##
##             estim    sqrt  nlvls  fixed                 factor
## sigma^2.1  0.0008  0.0284     28     no               paper_id
## sigma^2.2  0.0289  0.1700     73     no  paper_id/effect_size_id
##
## Test for Residual Heterogeneity:
## QE(df = 71) = 174.5910, p-val < .0001
##
## Test of Moderators (coefficient 2):
## F(df1 = 1, df2 = 71) = 0.3318, p-val = 0.5664
##
## Model Results:
##
##                estimate      se    tval  df    pval    ci.lb   ci.ub
## intrcpt          0.0737  0.0502  1.4665  71  0.1469  -0.0265  0.1738
## sex_percent_F    0.0653  0.1134  0.5760  71  0.5664  -0.1609  0.2916
##
## ---
## Signif. codes:  0 '***' 0.001 '**' 0.01 '*' 0.05 '.' 0.1 ' ' 1
```

```
model_CTI_multilevel_Rob
```

```
##
## Multivariate Meta-Analysis Model (k = 72; method: REML)
##
## Variance Components:
##
##             estim    sqrt  nlvls  fixed                   factor
## sigma^2.1  0.0037  0.0606     27     no                 paper_id
## sigma^2.2  0.0284  0.1684     72     no  paper_id/effect_size_id
##
## Test for Residual Heterogeneity:
## QE(df = 70) = 188.8290, p-val < .0001
##
## Test of Moderators (coefficients 1:2):
## F(df1 = 2, df2 = 70) = 5.3111, p-val = 0.0071
##
## Model Results:
##
##                        estimate      se    tval  df    pval    ci.lb   ci.ub
## factor(overall_RoB)1     0.1013  0.0338  2.9988  70  0.0038   0.0339  0.1687  **
## factor(overall_RoB)2     0.0983  0.0770  1.2764  70  0.2060  -0.0553  0.2518
##
## ---
## Signif. codes:  0 '***' 0.001 '**' 0.01 '*' 0.05 '.' 0.1 ' ' 1
```

```
model_Cogni_multilevel_Year <- rma.mv(yi = Cogni_cor_Z, V = Cogni_cor_Z_var, slab = Ref
_APA, data = Data, random = ~ 1 | paper_id/effect_size_id, test = "t", method = "REML",
mods = ~ year, tdist=TRUE)
```

```
## Warning: 41 rows with NAs omitted from model fitting.
```

```
model_Cogni_multilevel_Age <- rma.mv(yi = Cogni_cor_Z, V = Cogni_cor_Z_var, slab = Ref_
APA, data = Data, random = ~ 1 | paper_id/effect_size_id, test = "t", method = "REML",
mods = ~ age, tdist=TRUE)
```

```
## Warning: 43 rows with NAs omitted from model fitting.
```

```
model_Cogni_multilevel_Sex <- rma.mv(yi = Cogni_cor_Z, V = Cogni_cor_Z_var, slab = Ref_
APA, data = Data, random = ~ 1 | paper_id/effect_size_id, test = "t", method = "REML",
mods = ~ sex_percent_F, tdist=TRUE)
```

```
## Warning: 42 rows with NAs omitted from model fitting.
```

```
model_Cogni_multilevel_Rob <- rma.mv(yi = Cogni_cor_Z, V = Cogni_cor_Z_var, slab = Ref_
APA, data = Data, random = ~ 1 | paper_id/effect_size_id, test = "t", method = "REML",
mods = ~ factor(overall_RoB)-1, tdist=TRUE)
```

```
## Warning: 42 rows with NAs omitted from model fitting.
```

```
model_Cogni_multilevel_Year
```

```
##
## Multivariate Meta-Analysis Model (k = 121; method: REML)
##
## Variance Components:
##
##             estim    sqrt  nlvls  fixed                  factor
## sigma^2.1  0.0114  0.1066     39     no                paper_id
## sigma^2.2  0.0095  0.0974    121     no  paper_id/effect_size_id
##
## Test for Residual Heterogeneity:
## QE(df = 119) = 354.1891, p-val < .0001
##
## Test of Moderators (coefficient 2):
## F(df1 = 1, df2 = 119) = 1.5059, p-val = 0.2222
##
## Model Results:
##
##          estimate       se     tval   df    pval     ci.lb    ci.ub
## intrcpt   16.7599  13.5233   1.2393  119  0.2177  -10.0176  43.5374
## year      -0.0082   0.0067  -1.2272  119  0.2222   -0.0215   0.0050
##
## ---
## Signif. codes:  0 '***' 0.001 '**' 0.01 '*' 0.05 '.' 0.1 ' ' 1
```

```
model_Cogni_multilevel_Age
```

```
##
## Multivariate Meta-Analysis Model (k = 119; method: REML)
##
## Variance Components:
##
##             estim    sqrt  nlvls  fixed                  factor
## sigma^2.1  0.0120  0.1094     38     no                paper_id
## sigma^2.2  0.0096  0.0981    119     no  paper_id/effect_size_id
##
## Test for Residual Heterogeneity:
## QE(df = 117) = 347.1890, p-val < .0001
##
## Test of Moderators (coefficient 2):
## F(df1 = 1, df2 = 117) = 0.5320, p-val = 0.4672
##
## Model Results:
##
##          estimate      se     tval   df    pval    ci.lb   ci.ub
## intrcpt    0.2017  0.0627   3.2169  117  0.0017   0.0775  0.3259  **
## age       -0.0018  0.0025  -0.7294  117  0.4672  -0.0067  0.0031
##
## ---
## Signif. codes:  0 '***' 0.001 '**' 0.01 '*' 0.05 '.' 0.1 ' ' 1
```

```
model_Cogni_multilevel_Sex
```

```
##
## Multivariate Meta-Analysis Model (k = 120; method: REML)
##
## Variance Components:
##
##             estim    sqrt  nlvls  fixed                  factor
## sigma^2.1  0.0130  0.1140     38     no                paper_id
## sigma^2.2  0.0095  0.0976    120     no  paper_id/effect_size_id
##
## Test for Residual Heterogeneity:
## QE(df = 118) = 371.1344, p-val < .0001
##
## Test of Moderators (coefficient 2):
## F(df1 = 1, df2 = 118) = 0.0013, p-val = 0.9714
##
## Model Results:
##
##                 estimate      se    tval   df    pval    ci.lb   ci.ub
## intrcpt           0.1653  0.0373  4.4363  118  <.0001   0.0915  0.2390  ***
## sex_percent_F     0.0021  0.0593  0.0359  118  0.9714  -0.1154  0.1196
##
## ---
## Signif. codes:  0 '***' 0.001 '**' 0.01 '*' 0.05 '.' 0.1 ' ' 1
```

```
model_Cogni_multilevel_Rob
```

```
##
## Multivariate Meta-Analysis Model (k = 120; method: REML)
##
## Variance Components:
##
##             estim    sqrt  nlvls  fixed                  factor
## sigma^2.1  0.0137  0.1169     38     no                paper_id
## sigma^2.2  0.0093  0.0966    120     no  paper_id/effect_size_id
##
## Test for Residual Heterogeneity:
## QE(df = 117) = 372.4900, p-val < .0001
##
## Test of Moderators (coefficients 1:3):
## F(df1 = 3, df2 = 117) = 14.6794, p-val < .0001
##
## Model Results:
##
##                        estimate      se    tval   df    pval    ci.lb   ci.ub
## factor(overall_RoB)1    0.1805  0.0321  5.6301  117  <.0001   0.1170  0.2440
## factor(overall_RoB)2    0.1366  0.0404  3.3765  117  0.0010   0.0565  0.2166
## factor(overall_RoB)3    0.1931  0.1500  1.2869  117  0.2007  -0.1041  0.4902
##
## factor(overall_RoB)1  ***
## factor(overall_RoB)2  ***
## factor(overall_RoB)3
##
## ---
## Signif. codes:  0 '***' 0.001 '**' 0.01 '*' 0.05 '.' 0.1 ' ' 1
```

11.2 Moderator Analysis - Year + Data from Behnke & Kaczmarek, 2019

```
#import and store data from excel file
Data2 <- read_excel("C:/Users/macbe/OneDrive/Behnke Dropbox/MA CHT/Data.xlsx", sheet =
"year_pub_bias")
```

```
## New names:
## • `title` -> `title...5`
## • `title` -> `title...8`
## • `` -> `...22`
## • `` -> `...31`
```

```r
#View(Data2)

Data2 <- as.data.frame(Data2)

#z-transform correlation coefficients
Data2$CO_cor_Z <- .5 * log((1+Data2$CO_cor)/(1-Data2$CO_cor))
Data2$TPR_cor_Z <- .5 * log((1+Data2$TPR_cor)/(1-Data2$TPR_cor))
Data2$CTI_cor_Z <- .5 * log((1+Data2$CTI_cor)/(1-Data2$CTI_cor))


# calculate z variance
Data2$CO_cor_Z_var <- ifelse(is.na(Data2$CO_cor), NA, 1 / (Data2$n_performance - 3))
Data2$TPR_cor_Z_var <- ifelse(is.na(Data2$TPR_cor), NA, 1 / (Data2$n_performance - 3))
Data2$CTI_cor_Z_var <- ifelse(is.na(Data2$CTI_cor), NA, 1 / (Data2$n_performance - 3))


# calculate z variance squared
Data2$CO_cor_Z_var_Sq <- ifelse(is.na(Data2$CO_cor), NA, (1 / (Data2$n_performance -
3))^2)
Data2$TPR_cor_Z_var_Sq <- ifelse(is.na(Data2$TPR_cor), NA, (1 / (Data2$n_performance -
3))^2)
Data2$CTI_cor_Z_var_Sq <- ifelse(is.na(Data2$CTI_cor), NA, (1 / (Data2$n_performance -
3))^2)


model_CO_multilevel_Year <- rma.mv(yi = CO_cor_Z, V = CO_cor_Z_var, slab = Ref_APA, dat
a = Data2, random = ~ 1 | paper_id/effect_size_id, test = "t", method = "REML", mods =
~ year, tdist=TRUE) #did not converge
```

```
## Warning: 95 rows with NAs omitted from model fitting.
```

```r
model_TPR_multilevel_Year <- rma.mv(yi = TPR_cor_Z, V = TPR_cor_Z_var, slab = Ref_APA,
data = Data2, random = ~ 1 | paper_id/effect_size_id, test = "t", method = "REML", mods
= ~ year, tdist=TRUE)
```

```
## Warning: 102 rows with NAs omitted from model fitting.
```

```r
#model_CTI_multilevel_Year <- rma.mv(yi = CTI_cor_Z, V = CTI_cor_Z_var, slab = Ref_APA,
data = Data2, random = ~ 1 | paper_id/effect_size_id, test = "t", method = "REML", mods
= ~ year, tdist=TRUE)


model_CO_multilevel_Year #did not converge
```

```
##
## Multivariate Meta-Analysis Model (k = 86; method: REML)
##
## Variance Components:
##
##             estim    sqrt  nlvls  fixed                factor
## sigma^2.1  0.0156  0.1250     46     no              paper_id
## sigma^2.2  0.0109  0.1042     86     no  paper_id/effect_size_id
##
## Test for Residual Heterogeneity:
## QE(df = 84) = 193.2813, p-val < .0001
##
## Test of Moderators (coefficient 2):
## F(df1 = 1, df2 = 84) = 3.9173, p-val = 0.0511
##
## Model Results:
##
##          estimate       se     tval  df    pval    ci.lb    ci.ub
## intrcpt   23.4955  11.8215   1.9875  84  0.0501  -0.0129  47.0039  .
## year      -0.0116   0.0059  -1.9792  84  0.0511  -0.0232   0.0001  .
##
## ---
## Signif. codes:  0 '***' 0.001 '**' 0.01 '*' 0.05 '.' 0.1 ' ' 1
```

```
model_TPR_multilevel_Year
```

```
##
## Multivariate Meta-Analysis Model (k = 79; method: REML)
##
## Variance Components:
##
##             estim    sqrt  nlvls  fixed                factor
## sigma^2.1  0.0174  0.1320     42     no              paper_id
## sigma^2.2  0.0000  0.0000     79     no  paper_id/effect_size_id
##
## Test for Residual Heterogeneity:
## QE(df = 77) = 144.2935, p-val < .0001
##
## Test of Moderators (coefficient 2):
## F(df1 = 1, df2 = 77) = 1.5997, p-val = 0.2098
##
## Model Results:
##
##          estimate       se     tval  df    pval     ci.lb   ci.ub
## intrcpt  -14.2351  11.1563  -1.2760  77  0.2058  -36.4502  7.9800
## year       0.0070   0.0055   1.2648  77  0.2098   -0.0040  0.0180
##
## ---
## Signif. codes:  0 '***' 0.001 '**' 0.01 '*' 0.05 '.' 0.1 ' ' 1
```

```
#model_CTI_multilevel_Year #did not converge
```

11.3 Table for moderator analysis

```r
# Function to extract the necessary information from the moderator models
extract_mod_results <- function(model) {
  mod_F <- round(model$QM, 2)
  mod_df <- paste(model$QMdf[1], model$QMdf[2], sep = ", ")
  return(list(F = mod_F, df = mod_df))
}

# Creating the summary table
create_summary_table <- function() {
  data <- data.frame(
    Name = c("CO", "TPR", "CTI", "Cogni"),
    ModeratorAge_F = c(
      extract_mod_results(model_CO_multilevel_Age)$F,
      extract_mod_results(model_TPR_multilevel_Age)$F,
      extract_mod_results(model_CTI_multilevel_Age)$F,
      extract_mod_results(model_Cogni_multilevel_Age)$F
    ),
    ModeratorAge_df = c(
      extract_mod_results(model_CO_multilevel_Age)$df,
      extract_mod_results(model_TPR_multilevel_Age)$df,
      extract_mod_results(model_CTI_multilevel_Age)$df,
      extract_mod_results(model_Cogni_multilevel_Age)$df
    ),
    ModeratorSex_F = c(
      extract_mod_results(model_CO_multilevel_Sex)$F,
      extract_mod_results(model_TPR_multilevel_Sex)$F,
      extract_mod_results(model_CTI_multilevel_Sex)$F,
      extract_mod_results(model_Cogni_multilevel_Sex)$F
    ),
    ModeratorSex_df = c(
      extract_mod_results(model_CO_multilevel_Sex)$df,
      extract_mod_results(model_TPR_multilevel_Sex)$df,
      extract_mod_results(model_CTI_multilevel_Sex)$df,
      extract_mod_results(model_Cogni_multilevel_Sex)$df
    ),
    ModeratorBD_F = c(
      extract_mod_results(model_CO_multilevel_BD)$F,
      extract_mod_results(model_TPR_multilevel_BD)$F,
      extract_mod_results(model_CTI_multilevel_BD)$F,
      NA
    ),
    ModeratorBD_df = c(
      extract_mod_results(model_CO_multilevel_BD)$df,
      extract_mod_results(model_TPR_multilevel_BD)$df,
      extract_mod_results(model_CTI_multilevel_BD)$df,
      NA
    ),
    ModeratorYear_F = c(
      extract_mod_results(model_CO_multilevel_Year)$F,
      extract_mod_results(model_TPR_multilevel_Year)$F,
      NA, #did not converge
      extract_mod_results(model_Cogni_multilevel_Year)$F
    ),
```

```
    ModeratorYear_df = c(
      extract_mod_results(model_CO_multilevel_Year)$df,
      extract_mod_results(model_TPR_multilevel_Year)$df,
      NA, #did not converge
      extract_mod_results(model_Cogni_multilevel_Year)$df
    ),
    ModeratorRob_F = c(
      extract_mod_results(model_CO_multilevel_Rob)$F,
      extract_mod_results(model_TPR_multilevel_Rob)$F,
      extract_mod_results(model_CTI_multilevel_Rob)$F,
      extract_mod_results(model_Cogni_multilevel_Rob)$F
    ),
    ModeratorRob_df = c(
      extract_mod_results(model_CO_multilevel_Rob)$df,
      extract_mod_results(model_TPR_multilevel_Rob)$df,
      extract_mod_results(model_CTI_multilevel_Rob)$df,
      extract_mod_results(model_Cogni_multilevel_Rob)$df
    )
  )
  return(data)
}

# Create and print the summary table
summary_table <- create_summary_table()
print(summary_table)
```

```
##    Name ModeratorAge_F ModeratorAge_df ModeratorSex_F ModeratorSex_df
## 1    CO           1.18           1, 66           0.03           1, 66
## 2   TPR           0.08           1, 59           0.01           1, 59
## 3   CTI           0.02           1, 71           0.33           1, 71
## 4 Cogni           0.53          1, 117           0.00          1, 118
##   ModeratorBD_F ModeratorBD_df ModeratorYear_F ModeratorYear_df ModeratorRob_F
## 1          2.07          2, 66            3.92            1, 84           1.54
## 2          4.00          2, 59            1.60            1, 77           9.23
## 3          6.92          2, 71              NA             <NA>           5.31
## 4            NA           <NA>            1.51           1, 119          14.68
##   ModeratorRob_df
## 1           2, 65
## 2           2, 58
## 3           2, 70
## 4          3, 117
```

```
write.csv(summary_table, "moderator_table.csv", row.names = FALSE)
```
